# A Structure‐Guided Switch in the Regioselectivity of a Tryptophan Halogenase

**DOI:** 10.1002/cbic.201600051

**Published:** 2016-03-30

**Authors:** Sarah A. Shepherd, Binuraj R. K. Menon, Heidi Fisk, Anna‐Winona Struck, Colin Levy, David Leys, Jason Micklefield

**Affiliations:** ^1^ School of Chemistry and Manchester Institute of BiotechnologyThe University of Manchester131 Princess StreetManchesterM1 7DNUK

**Keywords:** aryl halides, biocatalysis, halogenation, mutagenesis, regioselectivity

## Abstract

Flavin‐dependent halogenases are potentially useful biocatalysts for the regioselective halogenation of aromatic compounds. Haloaromatic compounds can be utilised in the synthesis and biosynthesis of pharmaceuticals and other valuable products. Here we report the first X‐ray crystal structure of a tryptophan 6‐halogenase (SttH), which enabled key residues that contribute to the regioselectivity in tryptophan halogenases to be identified. Structure‐guided mutagenesis resulted in a triple mutant (L460F/P461E/P462T) that exhibited a complete switch in regioselectivity; with the substrate 3‐indolepropionate 75 % 5‐chlorination was observed with the mutant in comparison to 90 % 6‐chlorination for the wild‐type SttH. This is the first clear example of how regiocomplementary halogenases can be created from a single parent enzyme. The biocatalytic repertoire of SttH was also expanded to include a range of indolic and non‐indolic substrates.

Enzymes that can catalyse the regioselective halogenation of aromatic substrates could provide an attractive alternative to the traditional halogenation methods that are commonly used in synthesis. Halogenated aromatic compounds find extensive synthetic applications, particularly in transition‐metal‐catalysed crosscoupling reactions,^1^ and are important constituents of pharmaceuticals,^2^ agrochemicals^3^ and other valuable materials.^4^ Despite this, the traditional methods of producing haloaromatic compounds utilise harsh reaction conditions and often require harmful reagents, catalysts and solvents. These nonenzymatic methods also lack regiocontrol resulting in unwanted by‐products that can be difficult to separate and are problematic to dispose of owing to their toxicity or persistence in the environment.^5^ Consequently, there has been major interest in harnessing nature's halogenases, which employ benign inorganic halides in aqueous media, to effect cleaner and more regioselective halogenation reactions.

The first halogenating enzyme to be identified was the chloroperoxidase from the fungus, *Caldariomyces fumago*, which lacked regiocontrol owing to the free hypochlorous acid (HOCl) produced as the halogenating agent. Further examples of both haem‐ and vanadium‐dependent haloperoxidases were later identified, which also generally lacked substrate specificity and regioselectivity.^6^ More recently, Fe^2+^/α‐ketoglutarate (αKG)‐dependent and flavin‐dependent halogenases, which effect the regioselective halogenation of precursors in the biosynthesis of a wide range of halogenated natural products, have been identified.^7^ Many of the αKG‐ and flavin‐dependent halogenases utilise substrates that are tethered to the carrier proteins of biosynthetic assembly‐line enzymes thus making their use as biocatalysts limited. However, there are a number of flavin‐dependent tryptophan halogenases that can regioselectively halogenate free tryptophan, and therefore have more potential for synthetic purposes.^8^

Previously, X‐ray structures have been elucidated for the tryptophan 7‐halogenases, PrnA^9^ and RebH,^10^ as well as a tryptophan 5‐halogenase PyrH (Scheme 1).^11^ These structures provided insights into the mechanism and regiocontrol of flavin‐dependent halogenases. It is suggested, that the halogenases utilise O_2_ to oxidise FADH_2_ giving C4a‐hydroperoxyflavin, which then reacts with chloride to produce HOCl. It is then proposed that HOCl reacts with an active site lysine to generate a chloramine, which chlorinates the substrate.^9^, ^12^ Although the position of the active‐site lysine relative to the substrate is likely to be important in determining the regiochemical outcome of the reactions, the factors that effect the regiocontrol of these enzymes are still not fully understood. For example, using the PrnA and PyrH X‐ray crystal structures,^9^, ^11^ Lang et al. attempted to switch the regioselectivity of PrnA to that of PyrH by targeted mutagenesis.^13^ However, of all the mutants tested only one mutation, F103A, had any effect on the regioselectivity of PrnA. The F103A mutant showed a modest change in regioselectivity with bromide giving a 2:1 mixture of 7‐ and 5‐bromotryptophan, whereas the wild‐type PrnA gives exclusively 7‐bromotryptophan. This shift to produce 33 % 5‐bromotryptophan falls some way short of the change that would be required to create a new regiocomplementary enzyme. Here we describe the first structure of a tryptophan 6‐halogenase, SttH, which provides further insights into the factors affecting the regioselectivity of these flavin‐dependent halogenases. Moreover, these structural insights were used to expand the biocatalytic repertoire of this enzyme, and to guide mutagenesis leading to a complete switch in the regioselectivity from 90 % chlorination at the 6‐position to 75 % in favour of chlorination at the 5‐position of 3‐indolepropionic acid.

**Scheme 1:**
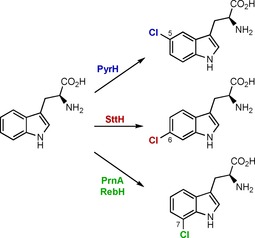
Reactions of flavin‐dependent halogenases with tryptophan, and their respective products.

Given that there is no structure for a tryptophan 6‐halogenase, we explored expression of a number of candidate enzymes for crystallography trials. From this, we found that SttH from *Streptomyces toxytricini* gave good expression in *Escherichia coli* and catalysed the halogenation of tryptophan to give exclusively 6‐chlorotryptophan, as reported previously.^14^ The X‐ray structure of SttH was then determined at 2.7 Å (Figure 1) to reveal a dimer, with each monomer exhibiting a box and triangular pyramid structure, as previously observed with PrnA, RebH and PyrH.^9^, ^10^, ^11^ Within the box structure are the conserved flavin‐dependent tryptophan halogenase sequences GxGxxG and WxWxIP. At the interface with the triangular pyramid, are the catalytic residues K79 and E363, which align with the active‐site lysine and glutamate of PrnA and PyrH (Figure 1 C).^14^, ^15^ In addition, SttH residues H96 and F98 are positioned for π‐stacking with the indole moiety of the substrate (Figure S2), whilst the P97 carbonyl and Y463 hydroxy groups can potentially hydrogen bond with the indole NH (Figure S3).

**Figure 1:**
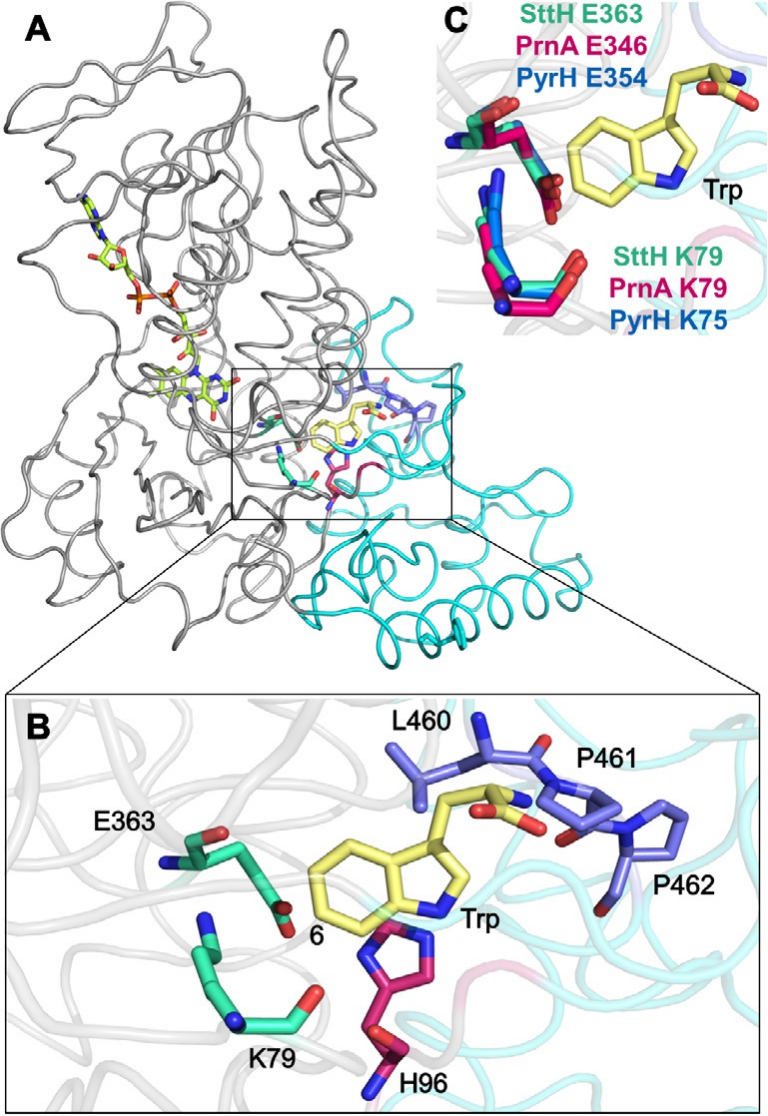
A) Crystal structure of SttH (PDB ID: 5HY5) showing typical box (grey) and triangular pyramid (cyan) of flavin‐dependent tryptophan halogenases. B) Selected active‐site residues of SttH with tryptophan modelled. C) Overlay of catalytic lysine and glutamate in PyrH, SttH and PrnA.

From sequence alignments it is evident that SttH is more like PyrH than PrnA, with insertions present in PyrH and SttH between residues SttH 155 and 167 and a deletion between SttH 457 and 464 compared with PrnA (Figure S1). These subtle differences around the active site of the enzymes lead to alterations in the binding mode of tryptophan and effect the regiocontrol observed with these enzymes. By comparing the apo structures of PyrH and SttH, it can also be noted that many of the other active‐site residues are very closely aligned, including sequences of residues such as QFPYAYHF (SttH residues 171–178) and PYYHGxxxYS (SttH residues 455–464). Other than residues between SttH G148 and G167, which lack electron density in the SttH structure, the only differences evident in the active‐site region between the structures of PyrH and SttH are those of PyrH residues F451, E452 and T453 and SttH L460, P461 and P462. These residues are of particular interest because they are in close proximity to the active site in PyrH and SttH, and are positioned directly above the α‐amino acid moiety of the substrate, tryptophan (**1**). Moreover, there is a loop insertion in PrnA in this region that is suggested to contribute to its regioselectivity.^11^ Each of these residues was mutated in SttH to the corresponding residue in PyrH, that is, L460F, P461E and P462T. Individually, each mutation reduced the relative activity of the enzyme with **1**, but did not have a significant effect on the observed regioselectivity, with 6‐chlorotryptophan (**1 a**) remaining the major product (Figure 2). Interestingly however, the triple mutant SttH L460F/P461E/P462T exhibited similar activity to the wild‐type SttH, with tryptophan as a substrate, but produced 32 % 5‐chlorotryptophan (**1 b**) and 68 % 6‐chlorotryptophan, whereas the wild‐type SttH only produces the 6‐chlorinated product.

**Figure 2:**
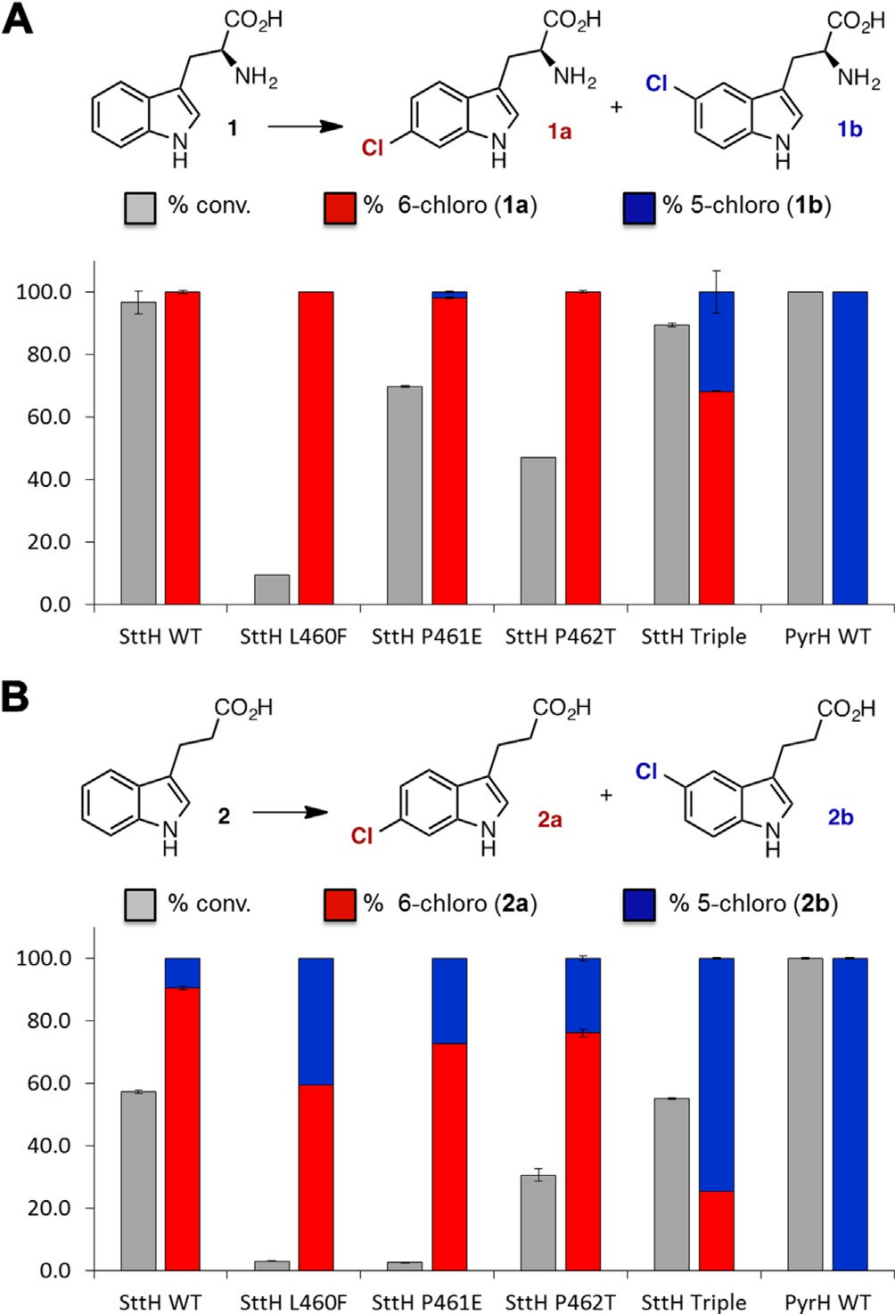
Percentage conversion of A) tryptophan or B) 3‐indolepropionic acid with SttH wild type, single and triple mutants, as well as PyrH wild type. Purified halogenase (10 μm) was incubated with agitation at 30 °C for 1 h with Fre (1 μm), GDH2 (6 μm), FAD (7.5 μm), NADH (200 μm), MgCl_2_ (50 mm), glucose (20 mm) and substrate (0.5 mm) in a total volume of 100 μL in potassium phosphate buffer (10 mm, pH 7.0).

A second substrate 3‐indolepropionic acid (**2**), which lacks the amino group of tryptophan and therefore has more flexibility in the active site owing to the absence of a potential interaction with the backbone carbonyl of SttH G459 (Figure S4), causes a more significant shift in regioselectivity than that of the tryptophan (Figure 2). With wild‐type SttH 10 % 5‐chloro‐3‐indolepropionic acid (**2 b**) is produced and 90 % 6‐chloro‐3‐indolepropionic acid (**2 a**), whereas the SttH triple mutant produces 75 % **2 b** and 25 % **2 a**. Notably, the relative activity of the SttH triple mutant was similar to that of the wild‐type enzyme, with 3‐indolepropionic acid (**2**) as substrate, thus indicating that the complete switch in regioselectivity can be achieved without impacting on catalytic efficiency. The wild‐type PyrH enzyme, with either **1** or **2** as a substrate, produced exclusively 5‐chlorinated products. In an effort to switch the regioselectivity of PyrH from 5‐ to 6‐halogenation, the corresponding PyrH triple mutant (F451L/E452P/T453P) was generated with the corresponding residues observed in SttH. However, this triple mutant was found to be inactive with both substrates **1** and **2**.

Previous studies have indicated that PrnA^16^ and RebH^17^ can halogenate indolic substrates, as well as tryptophan. Here we investigate the substrate specificity of SttH with *N*‐methyltryptophan (**3**) in addition to non‐indolic aromatic substrates such as kynurenine (**4**), anthranilamide (**5**) and other anilines (Table 1). As with the natural substrate **1**, halogenation occurs solely at the 6‐position of **3** resulting in 6‐chloro product (**3 a**). However, upon moving to the non‐indolic substrate **4**, chlorination did not occur at the 4‐position, *meta* to the amino group, as might be expected. Instead kynurenine is chlorinated at the intrinsically more reactive 5‐position by SttH. This suggests the greater flexibility of the kynurenine side chain, and perhaps also reduced π‐stacking interactions with H96 and F98 (Figure S2) compared with tryptophan, enables the active‐site lysine residue, K79, to deliver the electrophilic chloroamine to the most reactive 5‐position, *para* to the amino group. Presumably, the subtle differences in the active‐site architecture of SttH compared with PyrH are not sufficient to prevent the movement of kynurenine aryl group, so the more electronically favoured *para*‐chlorination reaction predominates. The same regioselectivity is also evident with the smaller aromatic substrates **5** and anthranilic acid (**6**), which are both solely chlorinated at the 5‐position. Finally, the biaryl compound *N‐*phenylanthranilic acid (**7**) is also halogenated by SttH, thus showing the potential for the halogenation of larger aromatic compounds with this enzyme.

**Table 1:** Conversion of substrates after 1 hour with SttH wild type.

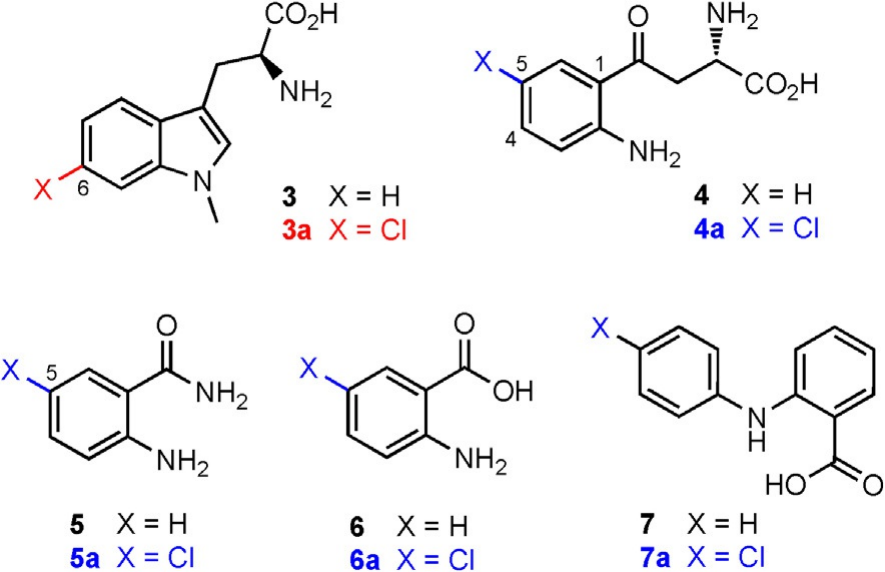		
Substrate	Conversion [%]	Product
tryptophan (**1**)	97±4	**1 a**
3‐indolepropionic acid (**2**)	57±1.5	**2 a**:**2 b** (9:1)
*N‐*methyltryptophan (**3**)	68±3	**3 a**
kynurenine (**4**)	79±4	**4 a**
anthranilamide (**5**)	43±2	**5 a**
anthranilic acid (**6**)	1.1±0.1	**6 a**
*N*‐phenylanthranilic acid (**7**)	20±2	**7 a**

Red indicates conversion to expected product. Blue indicates conversion to chemically favoured products. Assay conditions shown in Figure 2.

When comparing the activity of SttH with various substrates (Table 1), it is unsurprising that tryptophan was the best substrate. The addition of a methyl group to the nitrogen of the tryptophan indole ring (**3**) reduced activity. The activity was further decreased by the loss of the α‐amino group (**2**) and this also led to reduced regiocontrol, about 90 % 6‐chlorination observed (Figure 2). With non‐indolic substrates, the most electronically favoured products were produced with **4** (with its similar side chain to tryptophan) displaying close to 80 % conversion. Anilines **5** and **7** also displayed good conversion; however, upon switching the amide of **5** to the acid of **6**, activity is severely reduced.

From the kinetics of the selected substrates **1**, **4** and **5** (Table 2), it is evident that substrate binding has a significant effect on the overall catalytic efficiency of the enzyme. Generally, the turnover varies between 0.6 and 1.2 min^−1^; however, the *K*_m_ varied between 0.8 μm for tryptophan and 1 mm for anthranilamide. Presumably kynurenine has lower affinity for the active site, compared with tryptophan, owing to greater side chain flexibility and reduced π‐stacking interactions; this is consistent with the observed regioselectivity. In addition to this, anthranilamide also loses contacts with residues S54 and Q171, which are likely to bind the α‐amino acid moiety of **1** and **4**; this leads to even lower binding affinity (Figure S4).

**Table 2:** Kinetics of selected substrates with SttH wild type.

Substrate	*K*_m_ [μm]	*k*_cat_ [min^−1^]	*k*_cat_/*K*_m_×10^−3^ [min^−1^ μm ^−1^]
tryptophan (**1**)	0.8±0.1	0.65±0.02	825±95
kynurenine (**4**)	241±32	0.51±0.02	2.1±0.30
anthranilamide (**5**)	1075±154	1.21±0.04	1.1±0.17

Previously Frese et al. demonstrated that crosslinked enzyme aggregates (CLEAs) incorporating the 7‐chlorotrytophan halogenase RebH can be used to chlorinate the natural substrate tryptophan on a gram scale.^18^ By applying this method, CLEAs of SttH were produced and used to halogenate the unnatural substrate anthranilamide on a 100 mg scale (isolated yield 25 %). This could be improved by optimisation and catalyst recycling.

In summary, the first crystal structure of a tryptophan 6‐halogenase (SttH) has been determined. By comparing the structure of SttH with those of other halogenases, including PyrH, it is clear how subtle differences in the active site, π‐stacking interactions and contacts to the α‐amino acid moiety can alter the position of the aromatic moiety relative to the catalytic Lys residue thereby affecting the orientation of the subsequent electrophilic substitution reaction. The observed structural differences between the halogenases were exploited to create a SttH triple mutant, L460F/P461E/P462T, which showed the first complete switch in regioselectivity of this class of enzymes: with 3‐indolepropionate as substrate, wild‐type SttH gives 6‐chloro‐3‐indolepropionate, whereas 5‐chloro‐3‐indolepropionate was predominately produced by the triple mutant. The new regiocomplementary SttH variant displayed similar activity to the wild‐type enzyme. Further assays revealed an additional five substrates that can be regioselectively halogenated by SttH, and with CLEAs, the halogenase can be stabilised for use on a preparative scale. Taken together, these results provide guidance for future efforts to engineer regiocomplementary halogenases for a wider range of aromatic substrates of synthetic utility.^17^, ^18^, ^19^

## Supporting information

As a service to our authors and readers, this journal provides supporting information supplied by the authors. Such materials are peer reviewed and may be re‐organized for online delivery, but are not copy‐edited or typeset. Technical support issues arising from supporting information (other than missing files) should be addressed to the authors.

Supplementary
