# Supplementary material for: A Structure‐Guided Switch in the Regioselectivity of a Tryptophan Halogenase
Source: Chembiochem. 2016 Mar 30;17(9):821–4. doi: 10.1002/cbic.201600051 (PMC5071727; doi:10.1002/cbic.201600051)
Supplement: Supplementary file 1 — Supplementary [file CBIC-17-821-s001.pdf]

CHEMBIOCHEM

Supporting Information

A Structure-Guided Switch in the Regioselectivity of a Tryptophan Halogenase

Sarah A. Shepherd, Binuraj R. K. Menon, Heidi Fisk, Anna-Winona Struck, Colin Levy, David Leys, and Jason Micklefield*[a]

cbic_201600051_sm_miscellaneous_information.pdf

Content

Page S2-S9 Experimental

Page S10 Supplementary References

Page S11-S16 Supplementary Figures & Tables

Page S17-S39 HPLC, NMR, UV and MS data for halogenase reaction products.

Experimental Details

General All chemical reagents and solvents were purchased from Sigma-Aldrich Company Ltd, Fisher Scientific UK Ltd, or Alfa Aesar, a Johnson Matthey Company. All chemicals were used without further purification.

Cloning and expression of SttH A synthetic gene for the halogenase from *Streptomyces toxytricini* (SttH) was codon optimised using GeneArt® and obtained from Invitrogen (UK). The *sttH* gene was sub-cloned into the pET28a vector containing an N-terminal His-tag using the restriction enzymes *Nde*I and *NotI*. *E. coli* Arctic Express cells were subsequently transformed with the resulting SttH- containing vectors for overexpression of the recombinant halogenase. LB medium containing kanamycin (50 µg/mL) was inoculated with the transformant and incubated at 37 °C overnight. The cells were then diluted 1:100 in fresh TB medium and incubated shaking at 30 °C until an optical density (OD_{600nm}) of 0.6. The cells were subsequently incubated at 4 °C for 30 minutes for cold-shock, and protein expression was induced with addition of IPTG (0.1 mM), before growing for 24 hours at 15 °C, followed by harvesting of cells (4 °C, 20 min, 4000 x *g*,).

Cloning and expression of the flavin reductase (Fre) and glucose dehydrogenase (GDH2) The gene coding for the flavin reductase (Fre) from *E. col*[S1]*i* was amplified from *E. coli* BL21 genomic DNA using the oligonucleotides *fre* F and *fre* R (Table S1), then digested using the restriction enzymes *Kpn*I and *Xho*I, before ligating into the pET45b expression vector containing an N-terminal His-tag. *E. coli* BL21 (DE3) cells transformed with the recombinant Fre plasmid were initially grown overnight at 37°C in LB medium containing ampicillin (50 µg/mL) before diluting 1:100 in fresh LB medium the following day. Cultures were subsequently incubated at 37°C with shaking until an OD_{600} of 0.6. Recombinant protein overexpression was induced with IPTG (1 mM). Cultures were incubated for a further 5 h at 30°C before harvesting cells (4 °C, 20 min, 4000 x *g*,) and purification of recombinant N-terminal His tagged Fre.

A pET 21b vector containing a gene encoding GDH2 from *Bacillus megaterium*[S2] was kindly provided by Prof. Nigel Scrutton (University of Manchester). *E. coli* BL21 (DE3) cells transformed with the recombinant GDH2 plasmid were cultivated using the same method as described previously with induction of overexpression using 0.2 mM IPTG.

Cultures were incubated overnight at 18 °C before harvesting cells and purification of recombinant His tagged GDH2.

Protein purification Cell pellets derived from the *E. coli* protein expression, were resuspended in 25 mL lysis buffer (50 mM phosphate, 500 mM NaCl, and 10 mM imidazole, pH 7.0). Lysozyme (1 mg/mL) was added to the cell resuspension which was then incubated at 30 °C for 1 h. Cells were disrupted by sonication and the lysate was clarified by centrifugation (4 °C, 40 min, 10,000 x *g*). The soluble cell extract was loaded onto a HisTrap^{TM} FF crude column and purified by FPLC. The column was washed with 20 mM phosphate buffer (pH 7.0) containing 20 and 60 mM imidazole and 500 mM NaCl. Purified SttH was eluted in phosphate buffer containing 500 mM imidazole, Fre and GDH2 at 250 mM imidazole. Protein samples were subjected to buffer exchange with phosphate buffer containing 10 % glycerol using a Vivaspin 20 centricon (10,000 MWCO) before subsequently storing at −20 °C.

Mutagenesis The primers detailed in Table S1 were used to produce SttH and PyrH mutants using standard methods.

Biotransformations, and characterisation of activity and regioselectivity

Enzyme assays The following conditions were used for assays to determine regioselectivity and % conversions of halogenase reactions. Purified halogenase enzyme (10 µM) was incubated at 30 °C with shaking for 1 h with Fre (1 µM), GDH2 (6 µM), FAD (7.5 µM), NADH (200 µM), $MgCl_2$ (50 mM), glucose (20 mM) and substrate (0.5 mM) in a total volume of 100 µL in 10 mM potassium phosphate buffer, pH 7.0. Reactions were stopped by incubating at 95˚C for 5 min and precipitated protein was removed by centrifugation before analysis *via* HPLC on an Agilent Technologies 1260 system using an Agilent Zorbax Eclipse Plus C18 4.6 x 100 mm x 3.5 µm column. For tryptophan, 3-indolepropionic acid (33% isocratic gradient H_2O/acetonitrile + 0.1% formic acid) and *N*-phenylanthranilic acid (gradient 5 - 95% H_2O/acetonitrile + 0.1% formic acid), absorbance was measured at 280 nm. *N*-methyltryptophan, kynurenine, anthranilamide and anthranilic acid absorbance were measured at 254 nm, with a 5 min gradient 5 - 75% H_2O/acetonitrile + 0.1% formic acid.

Enzyme Kinetics To obtain kinetic parameters for selected reactions, the concentration of the assay components were varied according to conditions required to provide the best fit for the Michaelis-Menten curve. However in each case the total assay volume was 150 μL and the Fre concentration was always in excess in order to ensure the production of reduced flavin was not a rate limiting factor. Assays were performed at 30˚C with shaking at 800 rpm. Plates and assay components were pre-incubated at 30˚C. Assays were started by the addition of substrate using a multi-channel pipette. Substrate was added at 15 second intervals and the reaction terminated with the addition of formic acid. All assays were performed in triplicate.

Preparative halogenase reactions Larger scale assays were carried out to obtain chlorinated products for characterisation using halogenase (10 μM), Fre (1 μM), GDH2 (6 μM), substrate (2 mM), $MgCl_2$ (50 mM), FAD (7.5 μM), NADH (200 μM), glucose (20 mM) in 10 mM potassium phosphate buffer, pH 7.0 (10 mL). Assays were run at 30˚C with shaking. Reactions were stopped by incubating at 95 °C for 5 min and precipitated protein was removed by centrifugation (4 °C, 10 min, 12000 x *g*) before analysis by HPLC. For reactions with low yield, multiple 10 mL reactions were performed and combined prior to purification by semi-prep HPLC. For tryptophan, 3-indolepropionic acid (33% isocratic gradient H_2O/acetonitrile + 0.1% formic acid) and *N*-phenylanthranilic acid (5 - 95% H_2O/acetonitrile + 0.1% formic acid), absorbance was measured at 280 nm. All other substrates and products absorbance were measured at 254 nm, with a 5 min gradient 5 - 75% H_2O/acetonitrile + 0.1% formic acid. Products were subsequently characterised using 1D and 2D NMR and HRMS and are in good agreement with the literature data.

Preparation and assay with SttH combi CLEAs[18] pET28a-SttH was transformed into chemically competent *E. coli* BL21 (DE3) with the pGro7 plasmid encoding GroEL/ES chaperones. LB medium containing kanamycin (50 μg/mL) and chloramphenicol (35 μg/mL) was inoculated with the transformant and incubated at 37 °C overnight. The cells were then diluted 1:100 in fresh LB medium and incubated shaking at 37 °C until an optical density (OD_{600nm}) of 0.6. The cells were subsequently induced with addition of IPTG (0.2 mM) and L-arabinose (0.5 mg/mL), before growing for 18 hours at 18 °C, followed by harvesting of cells (4 °C, 20 min, 4000 x *g*,). Cells from 1.5 L of culture was resuspended in 25 mL 100 mM Na_2HPO_4 pH 7.4 and lysed by sonication. Following centrifugation (10,000 x *g*, 30 min, 4 °C), 2.5 U·mL flavin reductase Fre and 1 U·mL alcohol dehydrogenase was added to the lysate and mixed thoroughly. Ammonium sulphate (16.2 g) was added and the mixture cooled

and incubated at 4 °C for 2 hours with rocking to precipitate the proteins. Glutaraldehyde (0.5% w/v) was then added and the mixture incubated for a further 2 hours at 4 °C. The combi CLEAs were then spun down and washed (3 x 50 mL, 100 mM, Na_2HPO_4, pH7.4). The assay was carried out using the combi CLEAs, with 100 mg anthranilamide, 30 mM NaCl, 100 μM NAD^+, 10 μM FAD, 5% (v/v) isopropanol in 15 mM Na_2HPO_4 buffer pH 7.4 (150 mL) at 25°C overnight with shaking. 5-chloroanthranilamide was isolated (33 mg, 25%) using semi-prep HPLC.

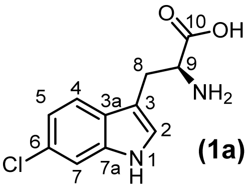

(1a)

6-chlorotryptophan (1a)[14] was prepared according to the general procedure described above with tryptophan (0.5 mM) and SttH to give 6-chlorotryptophan (91 % yield).
^{1}H NMR (400 MHz, D_2O) δ 7.51 (1H, d, $J_{4,5}$ = 8.7 Hz, ArH4), 7.42 (1H, s, ArH7), 7.18 (1H, s, ArH2), 7.04 (1H, d, $J_{5,4}$ = 8.7 Hz, ArH5), 4.06 (1H, dd, $J_{9,8A}$ = 7.8 Hz, $J_{9,8B}$ = 4.9 Hz, H9), 3.35 (1H, dd, $J_{8A,8B}$ = 15.7 Hz, $J_{8A,9}$ = 4.9 Hz, $H8_A$), 3.23 (1H, dd, $J_{8B,8A}$ = 15.7 Hz, $J_{8B,9}$ = 7.8 Hz, $H8_B$); LRMS-ESI (m/z) 239.0 (^{35}Cl), 241.0 (^{37}Cl) [M+H]; HRMS-ESI (m/z): calcd for [M+H] $C_{11}H_{12}N_2O_2{}^{35}Cl$ 239.0587 and $C_{11}H_{12}N_2O_2{}^{37}Cl$ 241.0558, found 239.0568 and 241.0539.

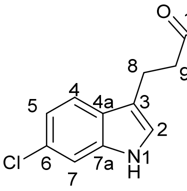

(2a)

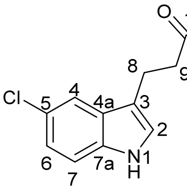

(2b)

Chlorination of 3-indolepropionic acid was carried out according to the general procedure described above with starting material 3-indolepropionic acid and enzyme SttH to give a mixture of 5 and 6-chlorinated products. 6-chloro-3-indolepropionic acid product (11% yield) was isolated by HPLC. 5-Chloro-3-indolepropionic acid (65% yield) was acquired using PyrH (10 μM) and was carried out in accordance with the general procedure stated above. **5-chloro-3-indolepropionic acid (2b)** ^{1}H NMR (800 MHz, MeOD) δ 7.51 (1H, d, $J_{4,6}$ = 2.0 Hz, ArH4), 7.28 (1H, d, $J_{7,6}$ = 8.6 Hz ArH7), 7.10 (1H, s, ArH2), 7.04 (1H, dd, $J_{6,7}$ = 8.6 Hz, $J_{6,4}$ = 2.0 Hz ArH6), 3.01 (2H, dd, $J_{8,9A}$ = 7.7 Hz, $J_{8,9B}$ = 7.5 Hz, H8), 2.65 (2H, dd, $J_{9,8A}$ = 7.7 Hz, $J_{9,8B}$ = 7.5 Hz, H9) ^{13}C NMR (201 MHz, MeOD) δ 175.9 (C10), 135.1 (C5) 128.2 (C7a), 123.9 (C3a), 123.3 (C2), 121.0 (C6), 117.2 (C4), 113.6 (C3), 111.9 (C7), 34.6 (C9), 20.2

(C8); HRMS-ESI (m/z): calcd for $C_{11}H_9NO_2{}^{35}Cl$ 222.0327 and $C_{11}H_9NO_2{}^{37}Cl$ 224.0297, found 222.0317 and 224.0285

6-chloro-3-indolepropionic acid (2a) ^{1}H NMR (800 MHz, MeOD) δ 7.49 (1H, d, $J_{4,5}$= 8.7 Hz, ArH4), 7.31 (1H, d, $J_{7,5}$= 1.8 Hz ArH7), 7.07 (1H, s, ArH2), 6.97 (1H, dd, $J_{5,4}$ = 8.7 Hz, $J_{5,7}$= 1.8 Hz ArH5), 3.03 (2H, dd, $J_{8,9A}$ = 7.8 Hz, $J_{8,9B}$= 7.5 Hz, H8), 2.65 (2H, dd, $J_{9,8A}$ = 7.8 Hz, $J_{9,8B}$= 7.5 Hz, H9) ^{13}C NMR (201 MHz, MeOD) δ 176.3 (C10), 137.0 (C6) 126.8 (C7a), 125.8 (C3a), 122.5 (C2), 118.9 (C4), 118.6 (C5), 114.1 (C3), 110.5 (C7), 34.8 (C9), 20.3 (C8); HRMS-ESI (m/z): calcd for $C_{11}H_9NO_2{}^{35}Cl$ 222.0327 and $C_{11}H_9NO_2{}^{37}Cl$ 224.0297, found 222.0317 and 224.0286

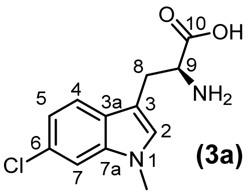

(3a)

6-chloro-*N*-methyltryptophan (3a) was prepared according to the general procedure described above with *N*-methyltryptophan and SttH to give 6-chloro-*N*-methyltryptophan (40 % yield). ^{1}H NMR (800 MHz, MeOD) δ 7.57 (1H, d, $J_{4,5}$= 8.4 Hz, ArH4), 7.43 (1H, d, $J_{7,5}$= 1.7 Hz ArH7), 7.14 (1H, s, ArH2), 7.07 (1H, dd, $J_{5,4}$ = 8.4 Hz, $J_{5,7}$= 1.7 Hz ArH5), 4.19 (1H, dd, $J_{9,8A}$ = 7.9 Hz, $J_{9,8B}$= 5.1 Hz, H9), 3.77 (3H, s, CH_3), 3.34 (1H, dd, $J_{8A,8B}$ = 15.4 Hz, $J_{8A,9}$ = 5.1 Hz, $H8_A$), 3.30 (1H, dd, $J_{8B,8A}$ = 15.4 Hz, $J_{8B,9}$ = 7.9 Hz, $H8_B$); ^{13}C NMR (201 MHz, MeOD) δ 171.8 (C10), 139.3 (C7a) 130.8 (C2), 129.1 (C3a), 127.5 (C6), 120.9 (C5), 120.6 (C4), 110.7 (C7), 107.9 (C3), 54.6 (C9), 33.0 (CH_3), 27.3 (C8); HRMS-ESI (m/z): calcd for $C_{12}H_{14}N_2O_2{}^{35}Cl$ 253.0744 and $C_{12}H_{14}N_2O_2{}^{37}Cl$ 255.0714, found 253.0727 and 255.0695

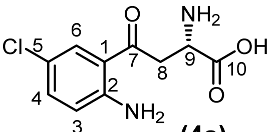

(4a)

5-chlorokynurenine (4a)[18b, S3] was prepared according to the general procedure described above with kynurenine and SttH to give 5-chlorokynurenine (46% yield). ^{1}H NMR (800 MHz, MeOD) δ 7.72 (1H, d, $J_{6,4}$ = 2.5 Hz, ArH6), 7.25 (1H, dd, $J_{4,3}$ = 9.0 Hz, $J_{4,6}$ = 2.5 Hz, ArH4), 6.78 (1H, d, $J_{3,4}$ = 9.0 Hz, ArH3), 4.37 (1H, dd, $J_{9,8A}$ = 7.1 Hz, $J_{9,8B}$= 3.6 Hz, H9), 3.68 (1H, dd, $J_{8A,8B}$ = 18.6 Hz, $J_{8A,9}$ = 3.6 Hz, $H8_A$), 3.64 (1H, dd, $J_{8B,8A}$ = 18.6 Hz, $J_{8B,9}$ = 7.1 Hz, $H8_B$); ^{13}C NMR (201 MHz, MeOD) δ 198.1 (C7), 171.9 (C10), 151.9 (C5) 135.9 (C4), 130.5 (C6), 120.0 (C3) 118.8 (C1) 117.5 (C2) 50.3 (C9) 39.6 (C8); LRMS-ESI m/z

243.0, 245.0 [M+H] HRMS-ESI (m/z): calcd for [M+H] $C_{10}H_{12}N_2O_3{}^{35}Cl$ 243.0536 and $C_{10}H_{12}N_2O_3{}^{37}Cl$ 245.0507, found 243.0517 and 245.0487.

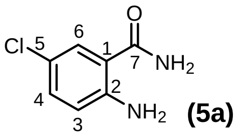

(5a)

5-chloroanthranilamide (5a)[18b, S4] was prepared according to the general procedure described above with anthranilamide and SttH to give 5-chloroanthranilamide (30% yield). ^1H NMR (400 MHz, MeOD) δ 7.53 (1H, d, $J_{6,4}$ = 2.4 Hz, ArH6), 7.15 (1H, dd, $J_{4,3}$ = 8.6 Hz, $J_{4,6}$ = 2.4 Hz, ArH4), 6.72 (1H, d, $J_{3,4}$ = 8.6 Hz, ArH3); LRMS-ESI (m/z) 171.0, 173.0 [M+H]; HRMS-ESI (m/z): calcd for [M+H] $C_7H_8N_2O^{35}Cl$ 171.0325 and $C_7H_8N_2O\ {}^{37}Cl$ 173.0296, found 171.0309 and 173.0278.

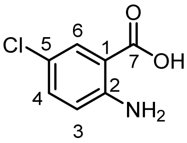

(6a)

5-chloroanthranilic acid (6a)[18b, S4] was prepared according to the general procedure described above with anthranilic acid and SttH to give 5-chloroanthranilic acid (12% yield) ^1H NMR (800 MHz, MeOD) δ 7.73 (1H, d, $J_{6,4}$ = 2.6 Hz, ArH6), 7.17 (1H, dd, $J_{4,6}$ =, 2.6 Hz, $J_{4,3}$ = 8.9 Hz, ArH4), 6.72 (1H, d, $J_{3,4}$ = 8.9 Hz, ArH3); ^{13}C NMR (800MHz/201 MHz, MeOD, HSQC,) δ 134.4 (C4), 131.3 (C6), 119.1 (C3); LRMS-ESI m/z 172.0, 173.9 [M+H]; HRMS-ESI (m/z): calcd for [M+H] $C_7H_7NO_2{}^{35}Cl$ 172.0165 and $C_7H_7NO_2{}^{37}Cl$ 174.0136, found 172.0141 and 174.0110.

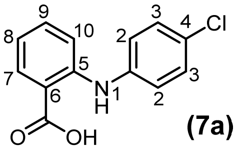

(7a)

***N*-(4-chlorophenyl)anthranilic acid (7a)**[18b, S5] was prepared according to the general procedure described above with *N*-phenylanthranilic acid and SttH to give *N*-(4-chlorophenyl)anthranilic acid (9% yield) ^1H NMR (800 MHz, MeOD) δ 7.99 (1H, d4, $J_{7,8}$ = 8.0 Hz, ArH7), 7.38 – 7.30 (1H, m, ArH9) 7.32 (2H, d, $J_{3,2}$ = 8.8 Hz, ArH3), 7.26 – 7.20 (1H, m, ArH10) 7.22 (2H, d, $J_{2,3}$ = 8.8 Hz, ArH2), 6.76 (1H, t, $J_{8,7}$ = 8.0 Hz, ArH8); ^{13}C NMR (800 MHz) δ 171.7 (C=O), 148.4 (C6) 141.1 (C4) 134.9 (C9), 133.1 (C7), 130.1(C3), 128.7 (C1) 123.8 (C2), 118.6 (C8), 114.9 (C10) LRMS-ESI m/z 248.0, 250.0 [M+H]; HRMS-ESI (m/z): calcd for [M+H] $C_{13}H_{11}NO_2{}^{35}Cl$ 248.0478 and $C_{13}H_{11}NO_2{}^{37}Cl$ 250.0449, found 248.0459 and 250.0428.

Enzyme Preparation, Crystallization, and Soaking For crystallographic trials, SttH from *Streptomyces toxytricini* was prepared by overexpressing in Origami™ 2(DE3) Singles™ competent cells from Novagen. Bacterial cells containing the pET28b SttH plasmid (Hexa His tagged at C and N terminal) were grown in 2YT medium at 37°C for 2 h, before decreasing the temperature to 24°C until OD_{600} 0.6. Recombinant protein overexpression was induced with IPTG (0.1 mM) and the cells incubated at 18°C for a further 24 h prior to harvesting by centrifugation (4°C, 20 min, 4000 x *g*,). Proteins were purified initially using an Ni^{2+} sepharose affinity tag procedure (as described previously), followed by loading onto a 140 ml Sephadex 200 size exclusion chromatography column (pre-equilibrated with 50 mM potassium phosphate, 500 mM NaCl, pH 7.2). Monomeric homogeneous enzyme solution was subsequently diluted (12.5 mg/ml) in crystallization buffer (20 mM HEPES, 100 mM NaCl, pH 7.2 buffer) and mixed in a 1:1 ratio with the precipitant solution. The composition of the precipitant solution was as follows: 0.1 M Bis Tris propane pH 6.5, 0.2 M sodium bromide, 20 % w/v PEG 3350). Crystals were grown by vapour diffusion at 4°C overnight.

Data Collection, Model Building, and Refinement Data were collected at Diamond Light Source from single cryofrozen crystals of SttH to a resolution of 2.7Å. (Table below). All data were processed and scaled using XDS and the structures solved by molecular replacement in Phaser (Search model 2AQJ). Iterative cycles of rebuilding and refinement were carried out in COOT and Phenix, validation with MOLPROBITY was integrated into the iterative rebuild process. Further validation of the final model was carried out using PDB_REDO. The crystal structure of SttH was deposited in the protein data bank (PDB file 5HY5).

Data Refinement Parameters

	STTH
Wavelength	0.9686
Resolution range	32.16 - 2.68 (2.776 - 2.68)
Space group	C 1 2 1
Unit cell	171.66 101.57 83.12 90 99.08 90
Total reflections	150393 (14554)
Unique reflections	39583 (3918)
Multiplicity	3.8 (3.7)
Completeness (%)	100 (100)
Mean I/sigma(I)	9.38 (1.91)
Wilson B-factor	34.98
R-merge	0.1496 (0.738)
R-meas	0.1744 (0.864)
CC1/2	0.984 (0.636)
CC*	0.996 (0.882)
Reflections used in refinement	39583 (3918)
Reflections used for R-free	2000 (198)
R-work	0.1979 (0.2982)
R-free	0.2370 (0.3871)
CC(work)	0.940 (0.754)
CC(free)	0.913 (0.709)
Number of non-hydrogen atoms	8139
macromolecules	7822
ligands	108
Protein residues	985
RMS(bonds)	0.695
RMS(angles)	4.65
Ramachandran favored (%)	94
Ramachandran allowed (%)	4.9
Ramachandran outliers (%)	0.72
Rotamer outliers (%)	3.1
Clashscore	5.42
Average B-factor	33.96
macromolecules	34.08
ligands	32.78
solvent	30.14

Statistics for the highest-resolution shell are shown in parentheses

Supplementary References

[S1] G. Spyrou, E. Haggård-Ljungquist, M. Krook, H. Jörnvall, E. Nilsson, P. Reichard, *J. Bacteriol.* **1991**, *173*, 3673–9.

[S2] T. Nagao, T. Mitamura, X. Wang, *J. Bacteriol.* **1992**, *174*, 5013–5020.

[S3] C. Maitrani, R. S. Phillips, *Bioorganic Med. Chem.* **2013**, *21*, 4670–4677.

[S4] H. Zhao, H. Fu, R. Qiao, *J. Org. Chem.* **2010**, *75*, 3311–3316.

[S5] A. O. Adeniji, B. M. Twenter, M. C. Byrns, Y. Jin, M. Chen, J. D. Winkler, T. M. Penning, *J. Med. Chem.* **2012**, *55*, 2311–23.

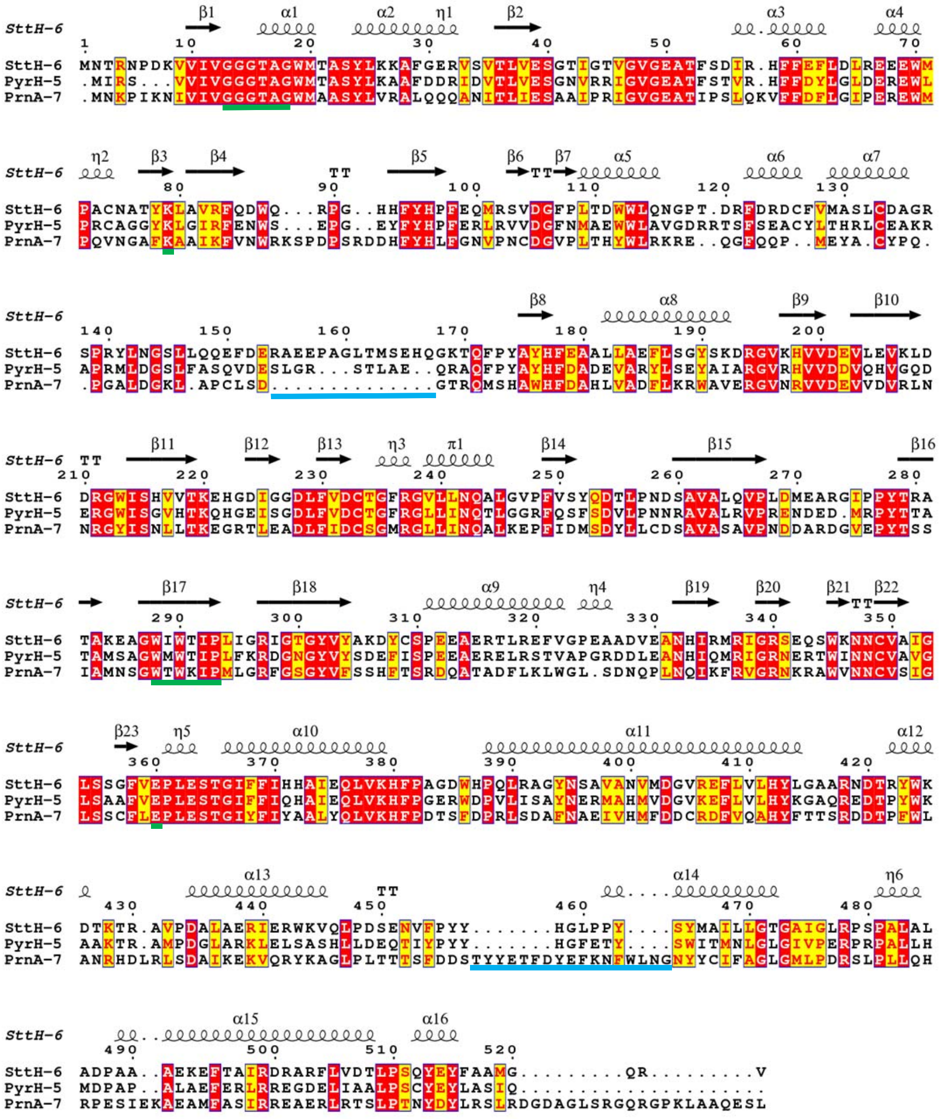

Figure S1. Sequence alignment of PyrH, SttH and PrnA, tryptophan 5, 6, 7-halogenases respectively. Identical residues shown in red. Key conserved motifs underlined in green. Insertions and deletions underlined in blue. Secondary structure assigned from the SttH crystal structure obtained during this study. Diagram produced using ESPript.

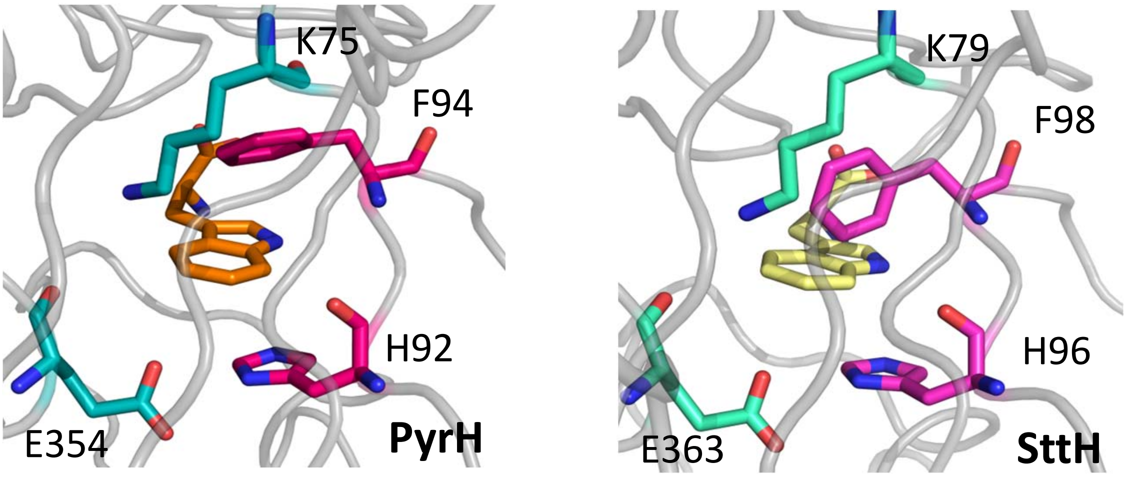

Figure S2. PyrH crystal structure (PDB 2WET) and SttH crystal structure (PDB 5HY5) showing substrate tryptophan, catalytic residues and π-stacking residues. Substrate is modelled in the case of SttH and therefore π-stacking residue SttH F98 like to rotate on binding.

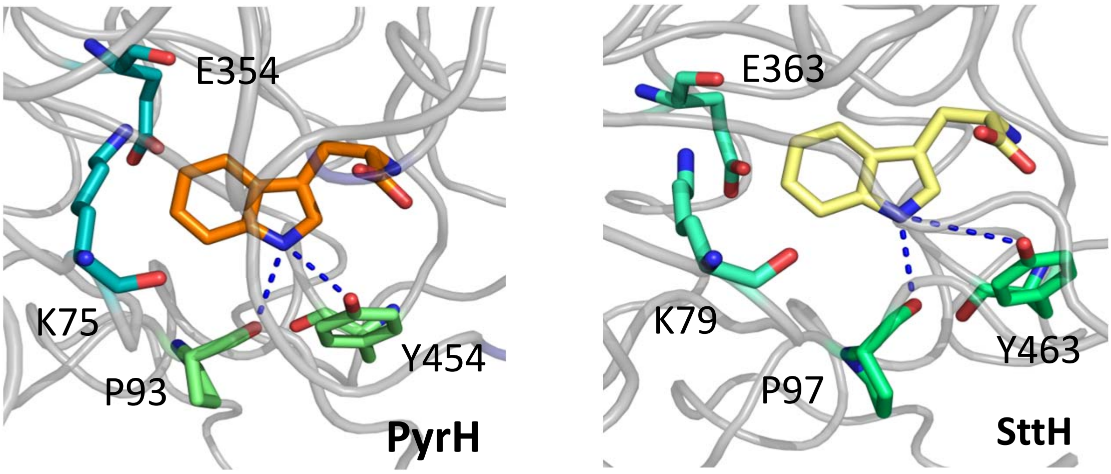

Figure S3. PyrH crystal structure (PDB 2WET) and SttH crystal structure (PDB 5HY5) showing substrate tryptophan, catalytic residues and residues interacting with indole NH.

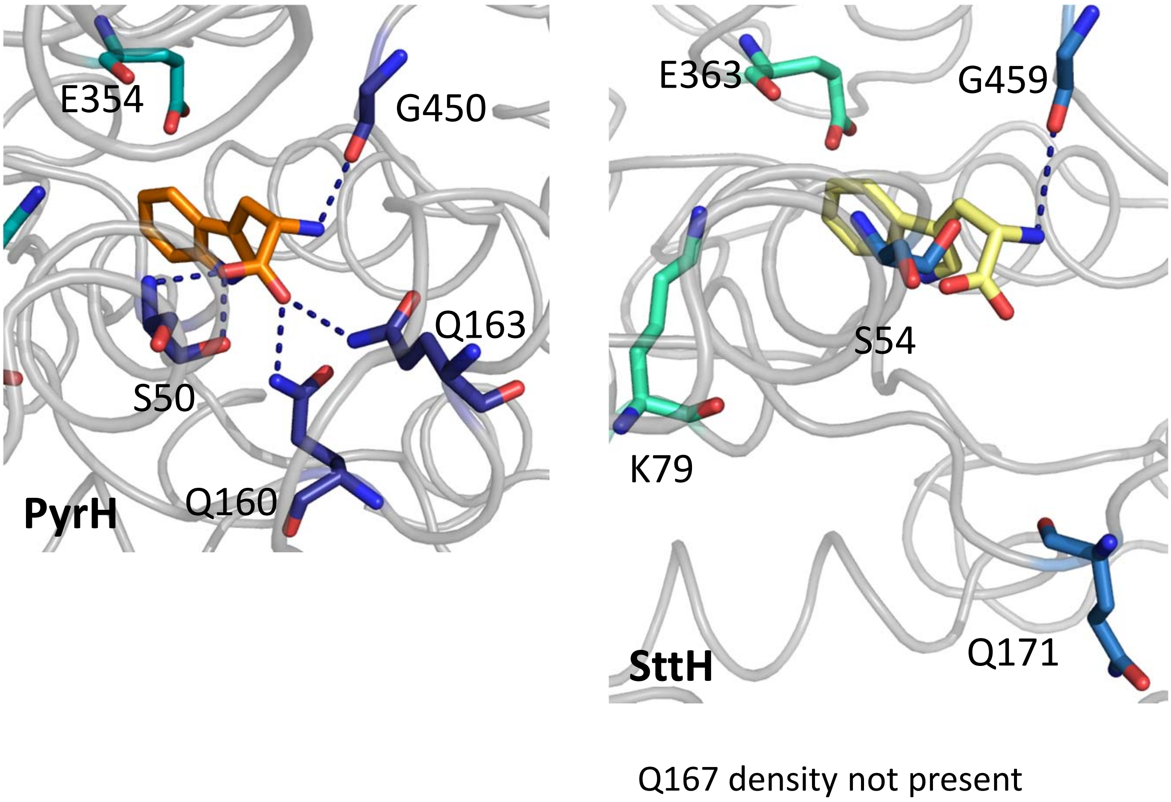

Figure S4. PyrH crystal structure (PDB 2WET) and SttH crystal structure (PDB 5HY5) showing substrate tryptophan, catalytic residues and side chain hydrogen bonding residues. Substrate is modelled in the case of SttH and therefore residue SttH Q171 is likely to flip into bind in a similar manner to PyrH Q163. Electron density is not present for SttH Q167 which is likely to interact in a similar manner to PyrH Q160

Primer	Sequence
fre F	5'-AAAAAAGGTACCATGACAACCTTAAGCTGTAAA-3’
fre R	3’-AAAAAACTCGAGTCAGATAAATGCAAACGCATC-5’
sttH L460F F	CCGTATTATCATGGTTTTCCTCCGTATAGTTAT
sttH L460F R	ACTATAACTATACGGAGGAAAACCATGATAATA
sttH P461E F	CCGTATTATCATGGTCTGGAACCGTATAGTTAT
sttH P461E R	ACTATAACTATACGGTTCCAGACCATGATAATA
sttH P462T F	CCGTATTATCATGGTCTGCCTACGTATAGTTAT
sttH P462T R	ACTATAACTATACGTAGGCAGACCATGATAATA
sttH LPP460FET F	TTCCCGTATTATCATGGTTTTGAAACGTATAGTTATATGGCAATT
sttH LPP460FET R	AATTGCCATATAACTATACGTTTCAAAACCATGATAATACGGGAA
pyrH FET450LPP F	TATCCGTATTATCATGGCCTTCCACCCTACAGCTGGATTACCATG
pyrH FET450LPP R	CATGGTAATCCAGCTGTAGGGTGGAAGGCCATGATAATACGGATA

Table S1. Primers used in this study

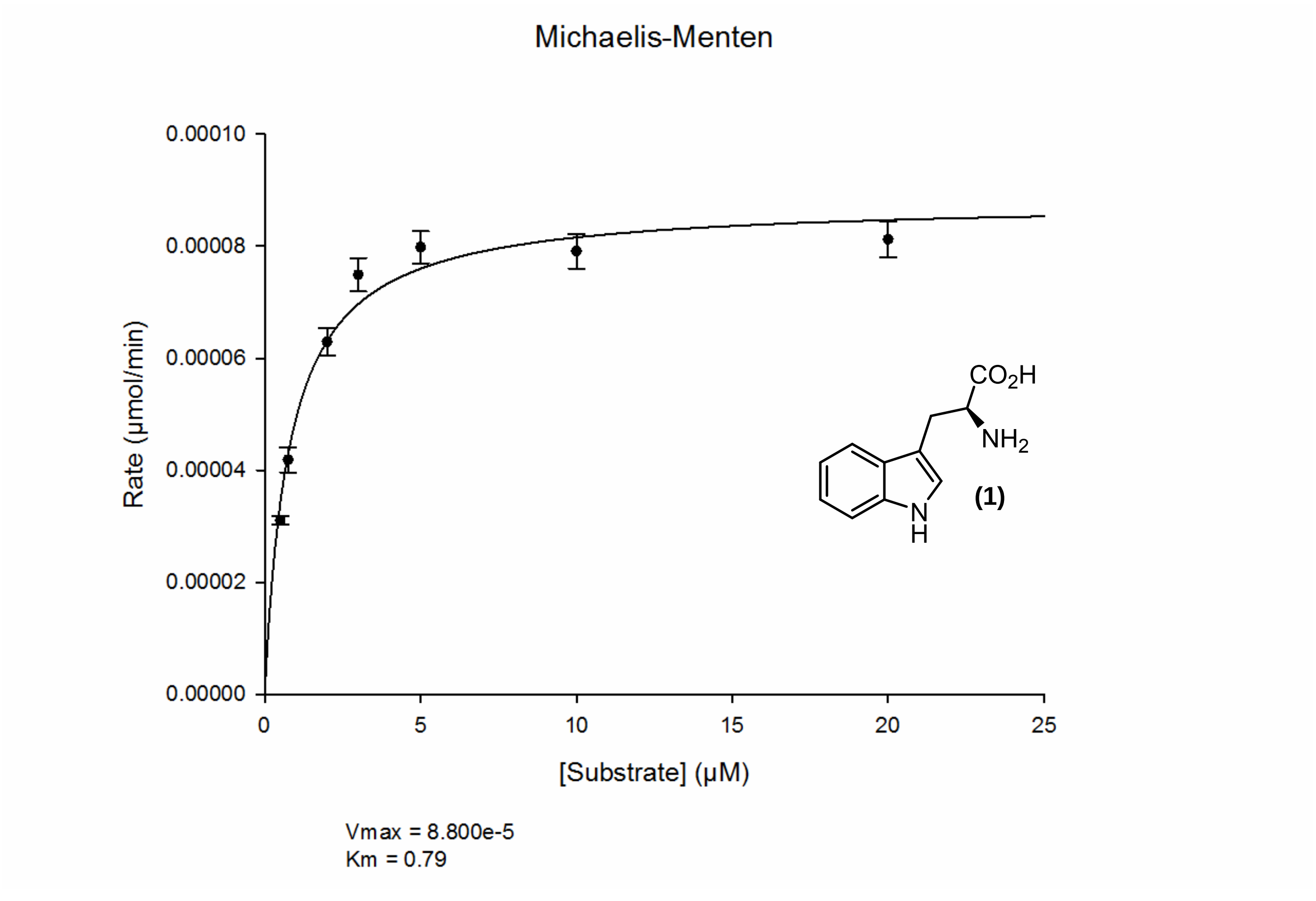

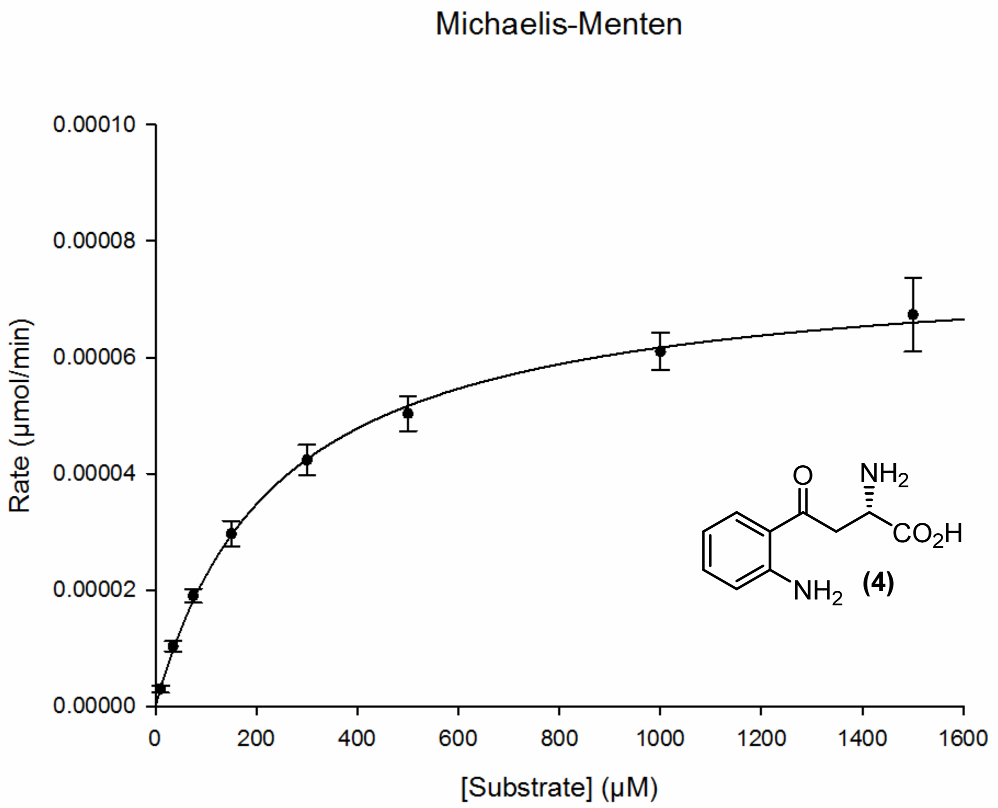

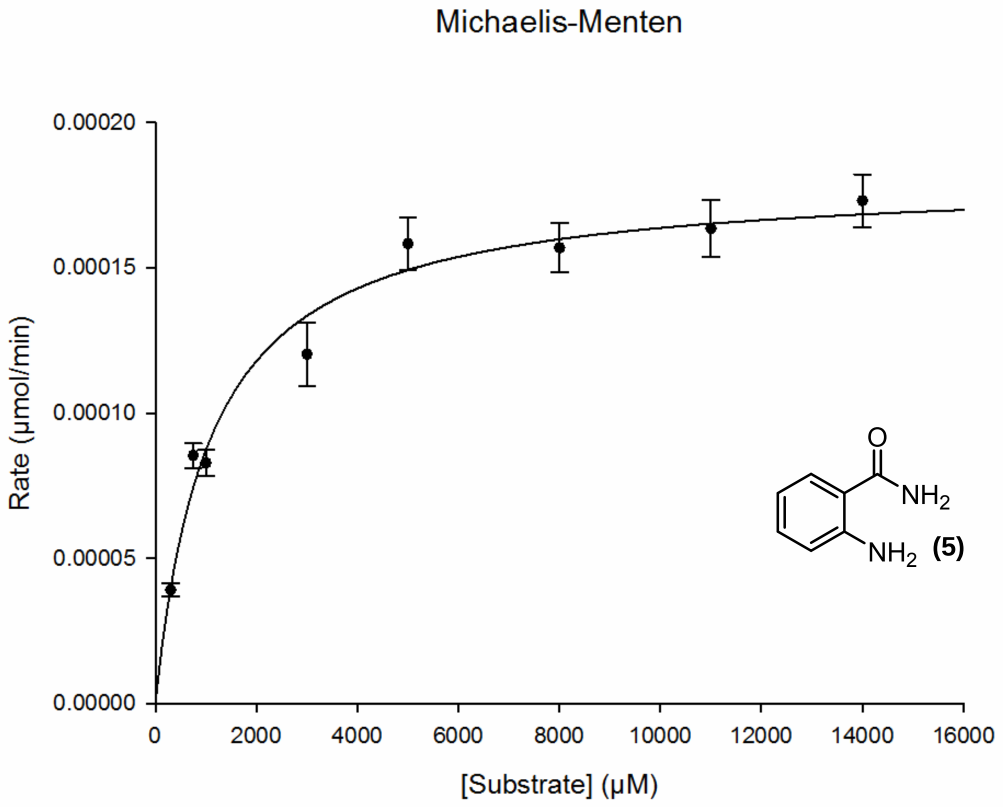

Vmax = 0.0001813
Km = 1075.3

Original data

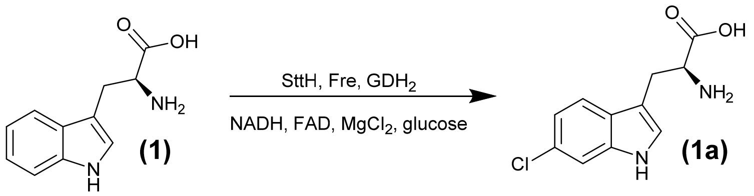

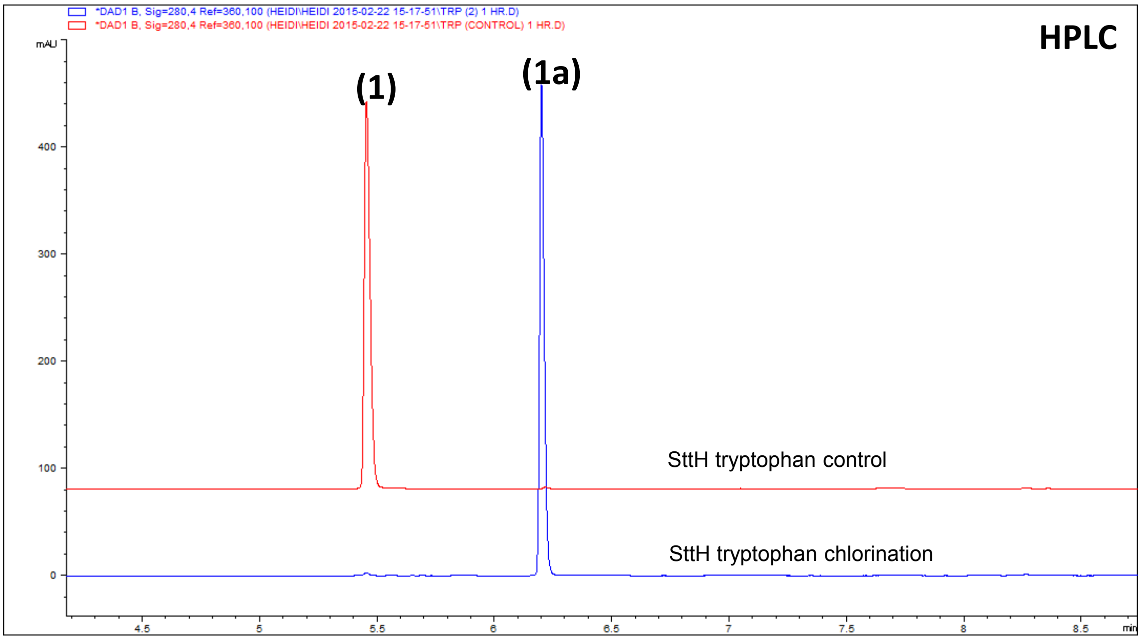

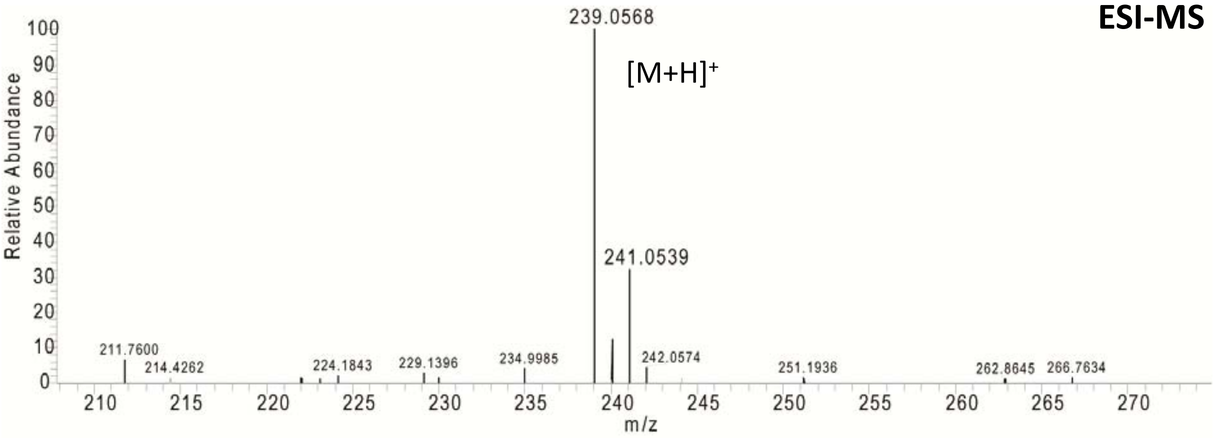

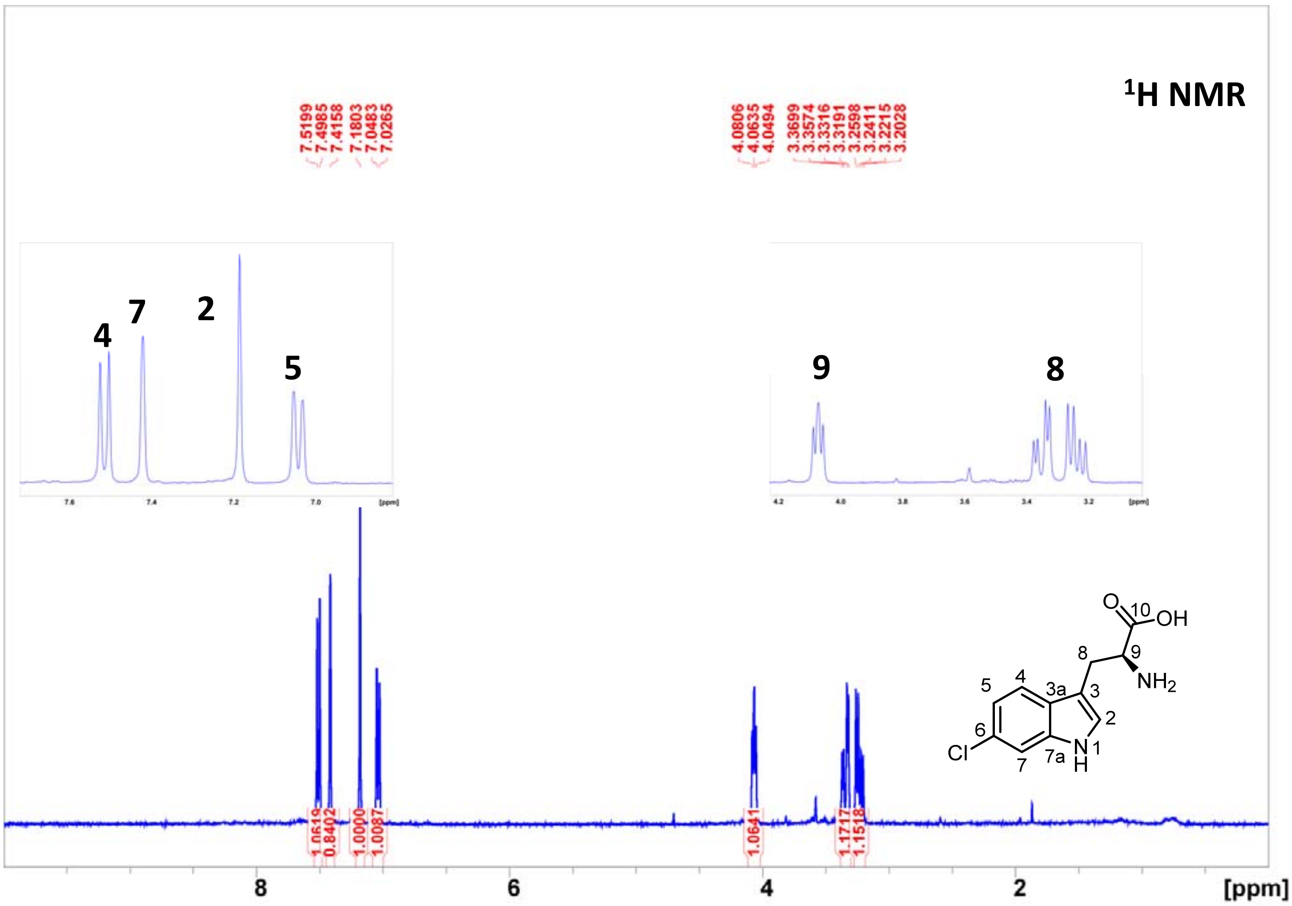

^{1}H NMR
7.5199
7.4985
7.4158
7.1803
7.0483
7.0265
4.0806
4.0635
4.0494
3.3699
3.3574
3.3316
3.3191
3.2598
3.2411
3.2215
3.2028
4
7
2
5
9
8
1.0619
0.8402
1.0000
1.0087
1.0641
1.1717
1.1518
8
6
4
2
[ppm]

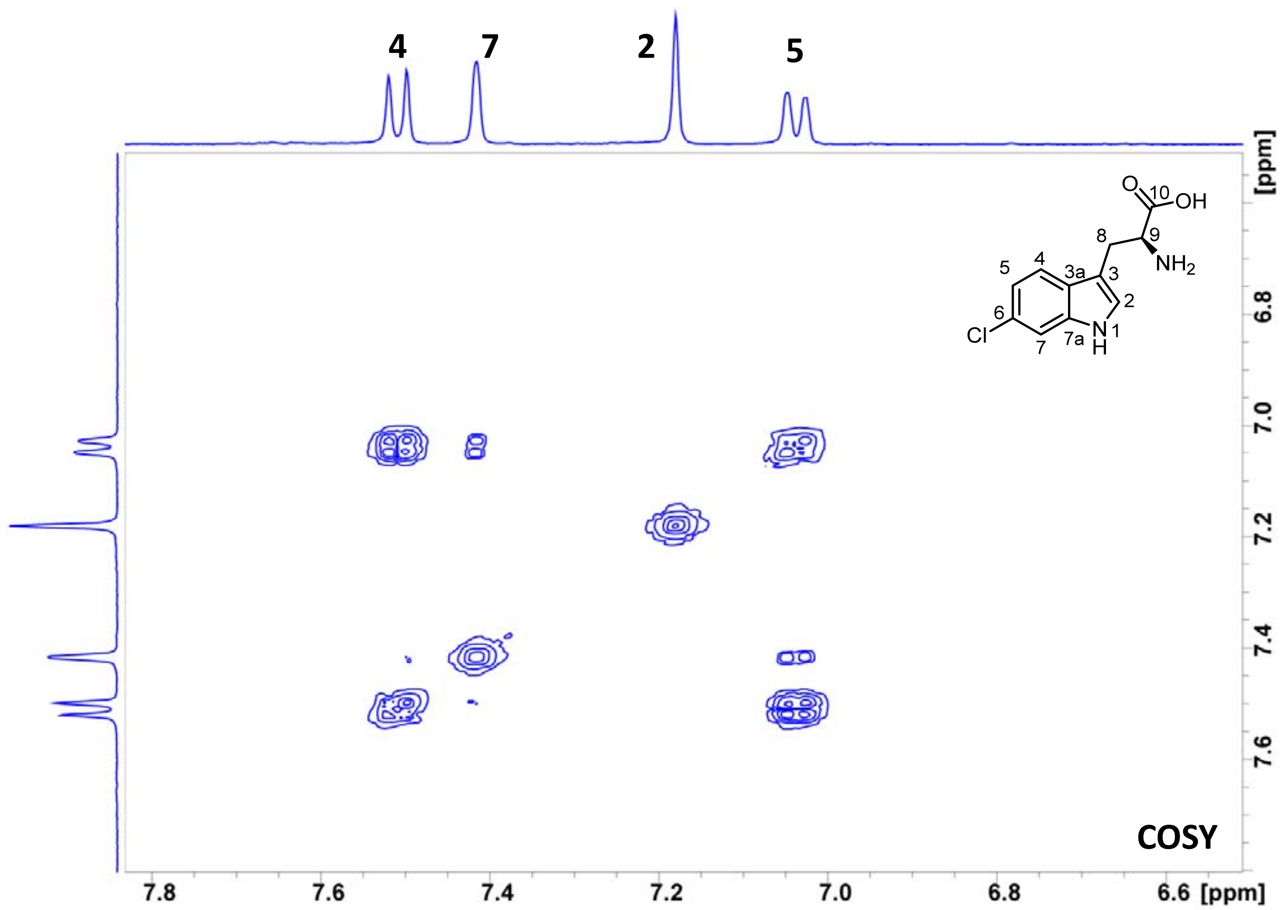

4
7
2
5
COSY
7.8
7.6
7.4
7.2
7.0
6.8
6.6 [ppm]

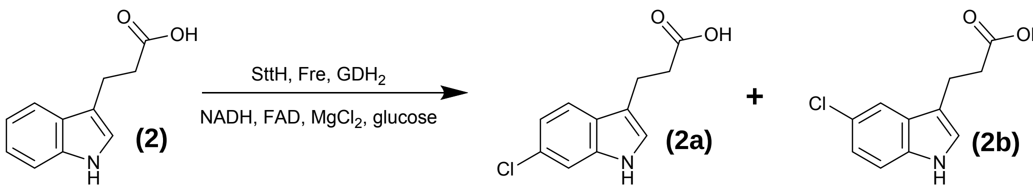

(2)
SttH, Fre, GDH$_2$
NADH, FAD, $MgCl_2$, glucose
(2a)
+
(2b)

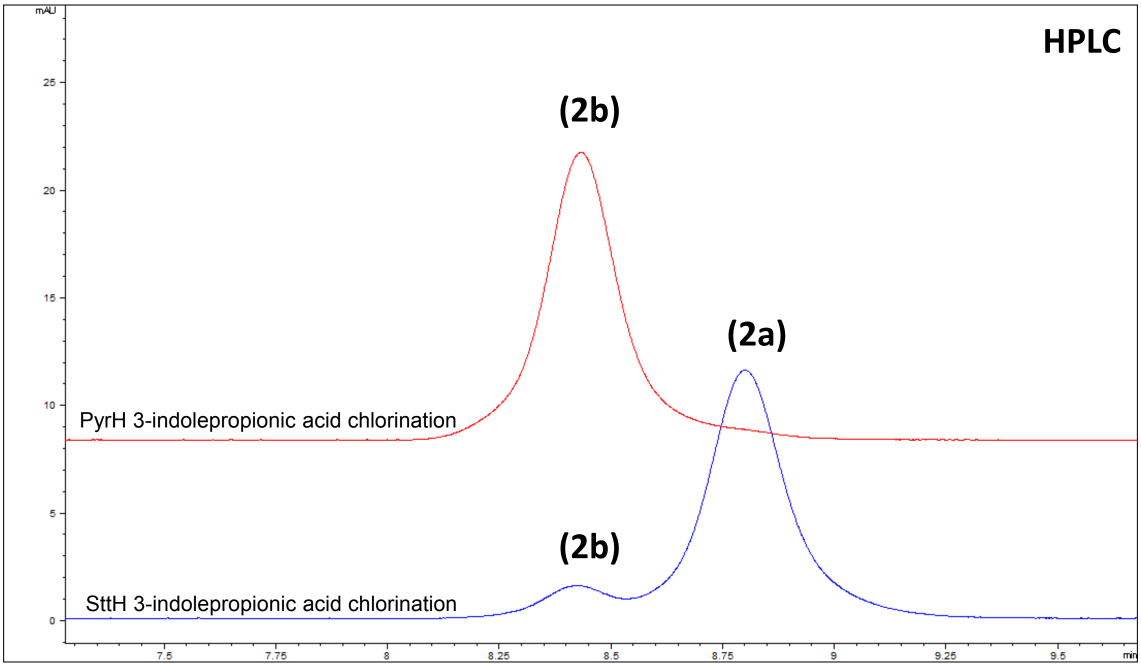

HPLC
mAU
(2b)
(2a)
PyrH 3-indolepropionic acid chlorination
(2b)
SttH 3-indolepropionic acid chlorination
min

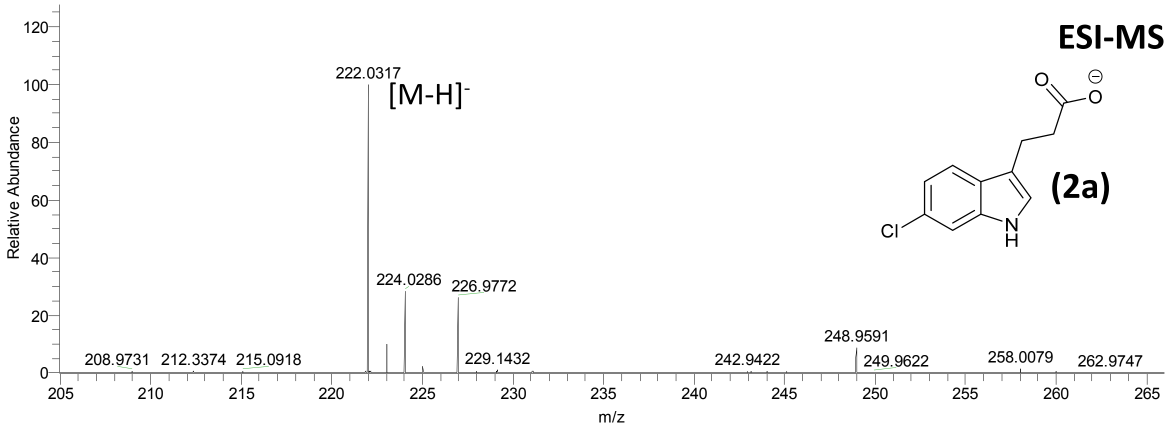

ESI-MS
Relative Abundance
222.0317
[M-H]$^-$
224.0286
226.9772
208.9731
212.3374
215.0918
229.1432
242.9422
248.9591
249.9622
258.0079
262.9747
(2a)
m/z

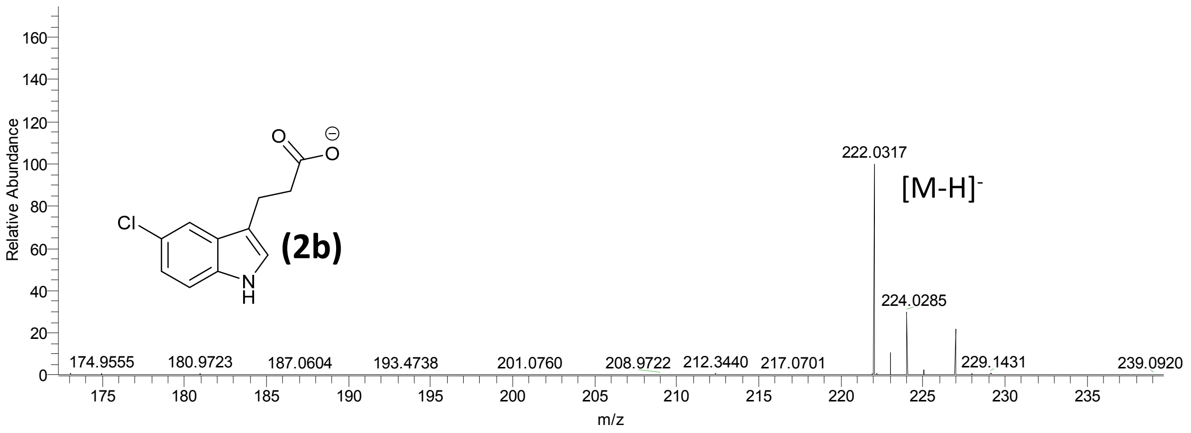

Relative Abundance
(2b)
222.0317
[M-H]$^-$
224.0285
174.9555
180.9723
187.0604
193.4738
201.0760
208.9722
212.3440
217.0701
229.1431
239.0920
m/z

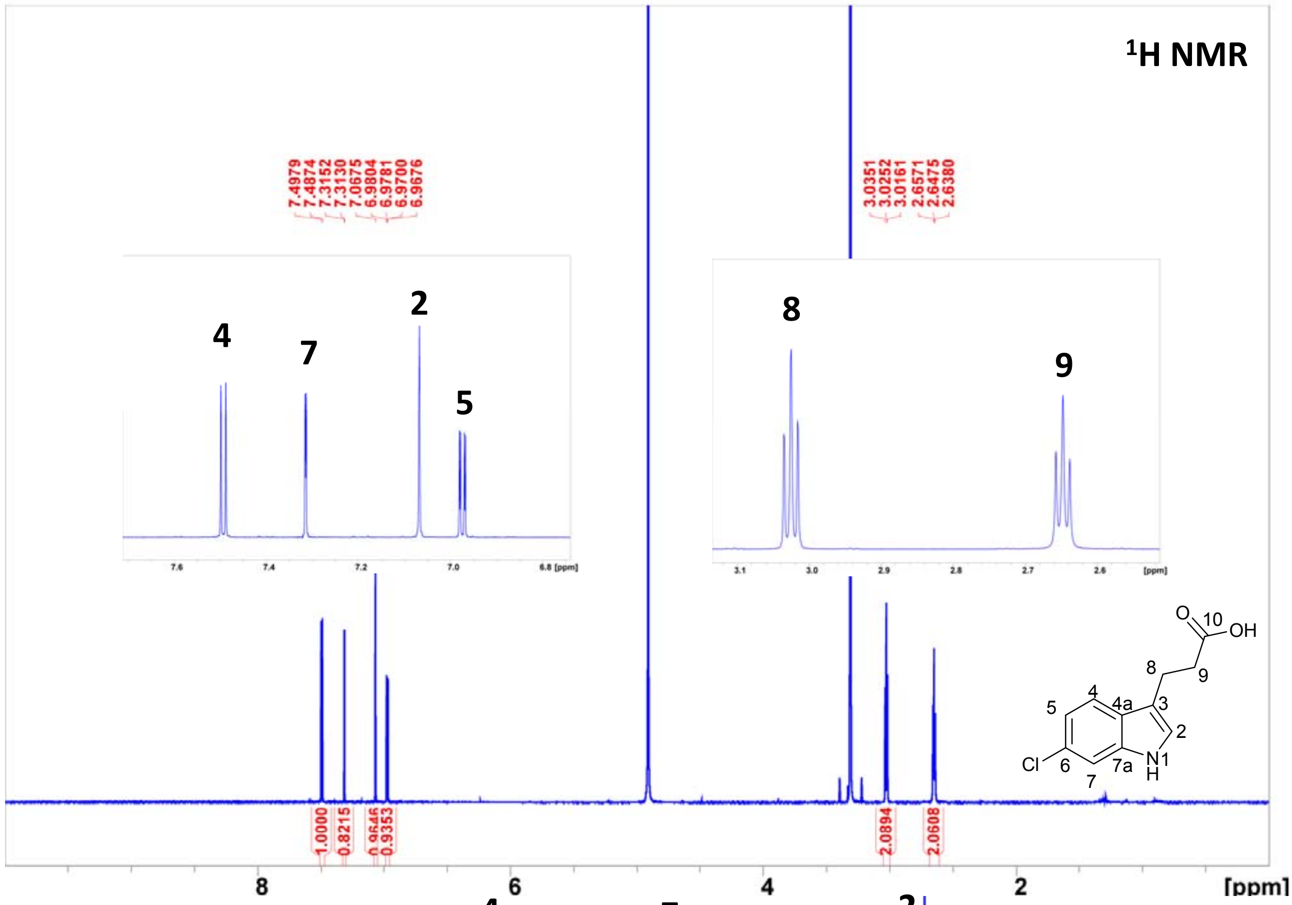

1H NMR
7.4979
7.4874
7.3152
7.3130
7.0675
6.9804
6.9781
6.9700
6.9676
3.0351
3.0252
3.0161
2.6571
2.6475
2.6380
4
7
2
5
8
9
1.0000
0.8215
0.9646
0.9353
2.0894
2.0608
8
6
4
2
[ppm]
O
10
OH
8
9
5
4
4a
3
2
Cl
6
7
7a
N1
H

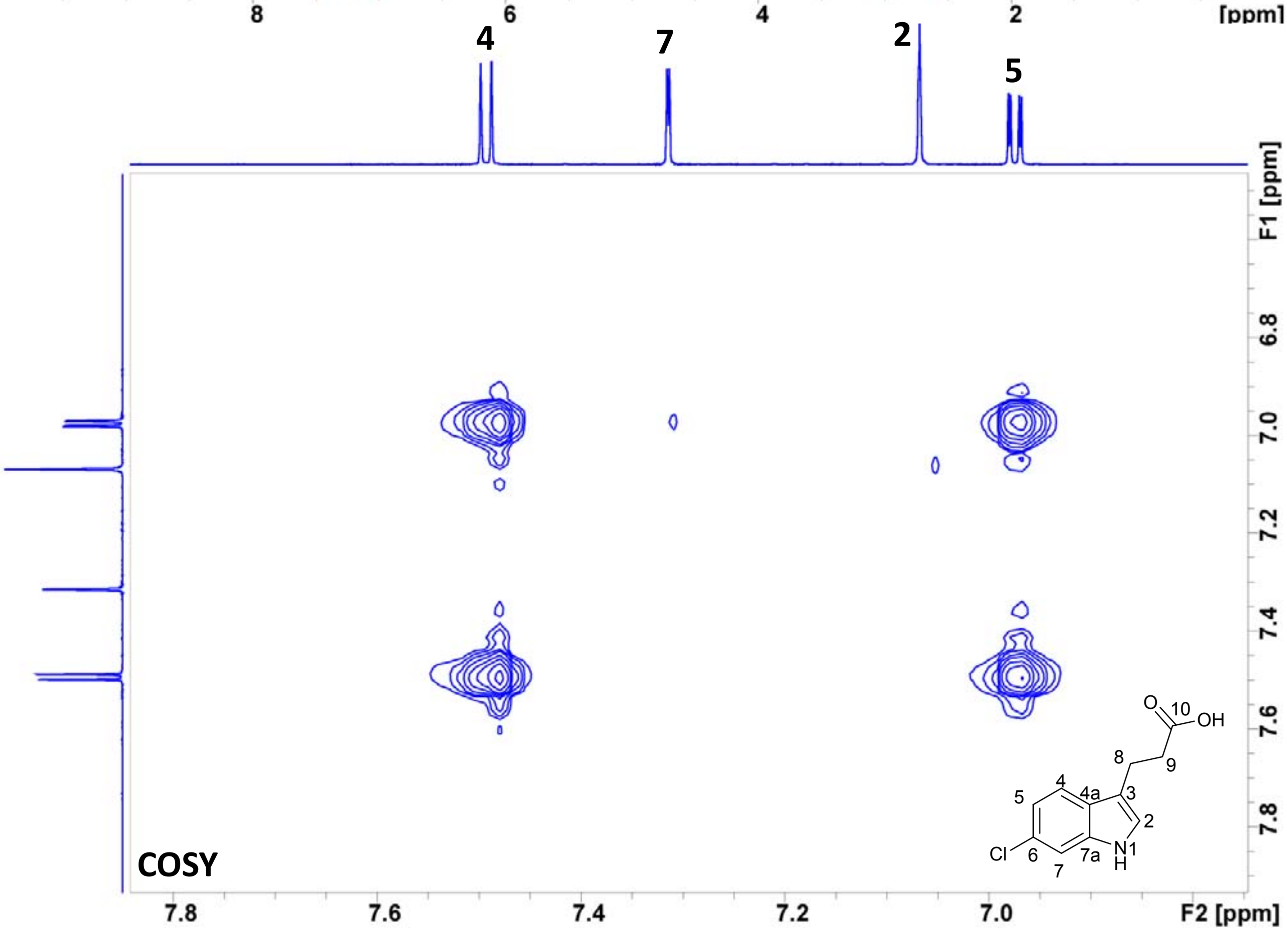

4
7
2
5
F1 [ppm]
6.8
7.0
7.2
7.4
7.6
7.8
COSY
7.8
7.6
7.4
7.2
7.0
F2 [ppm]
O
10
OH
8
9
5
4
4a
3
2
Cl
6
7
7a
N1
H

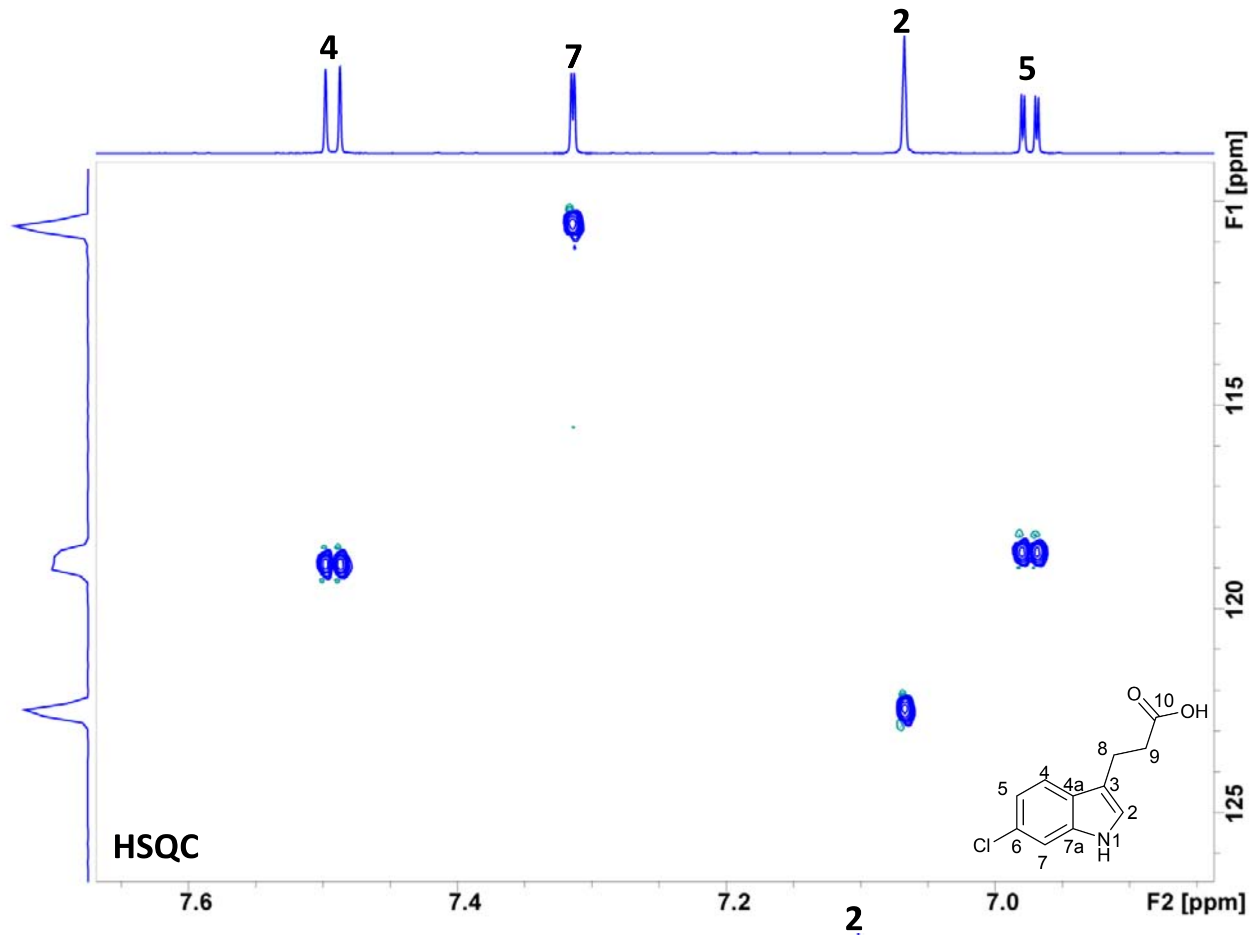
4
7
2
5
F1 [ppm]
115
120
125
HSQC
O
10
OH
8
9
4
5
4a
3
2
Cl
6
7a
N1
H
7
7.6
7.4
7.2
7.0
F2 [ppm]

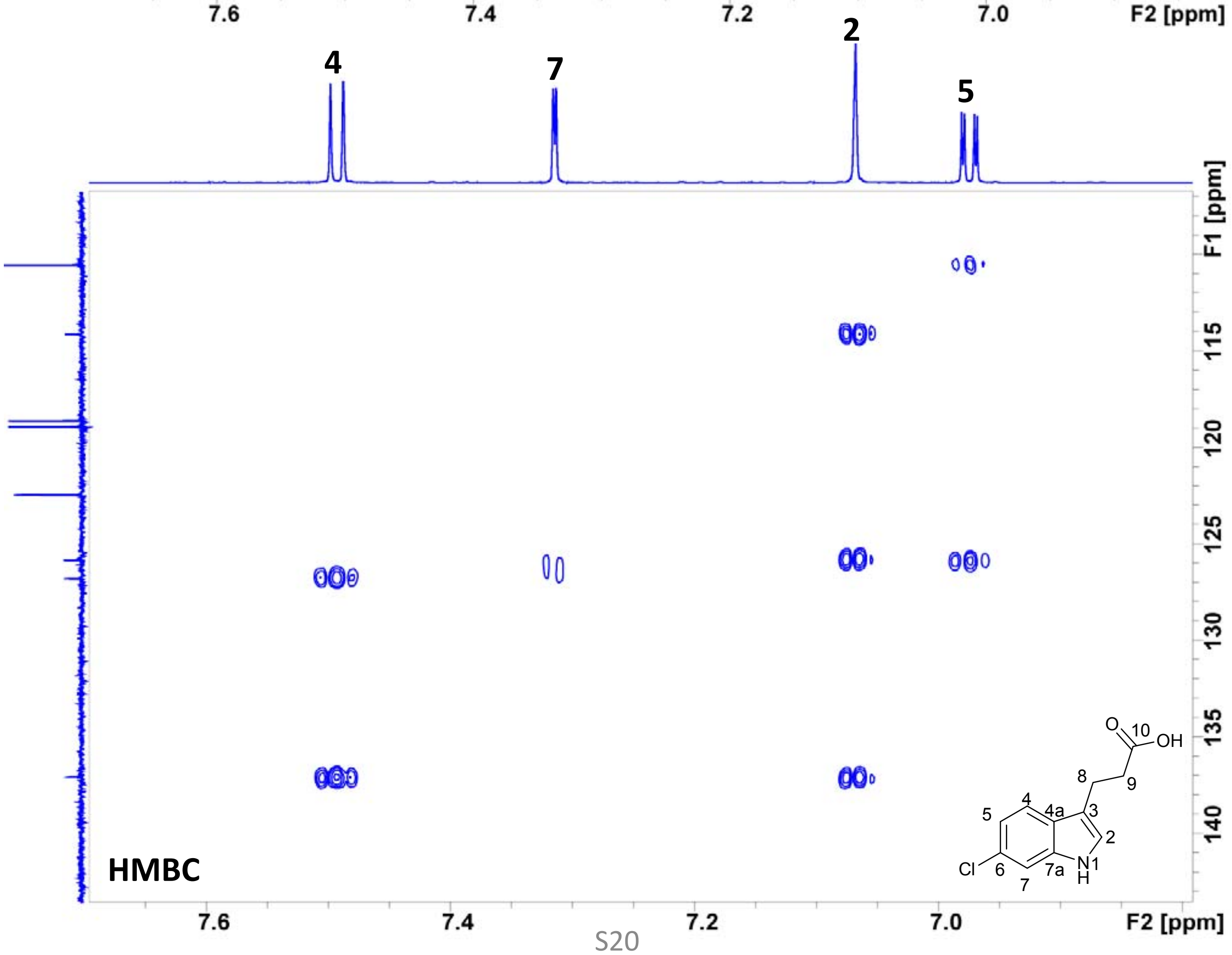
4
7
2
5
F1 [ppm]
115
120
125
130
135
140
HMBC
O
10
OH
8
9
4
5
4a
3
2
Cl
6
7a
N1
H
7
7.6
7.4
7.2
7.0
F2 [ppm]

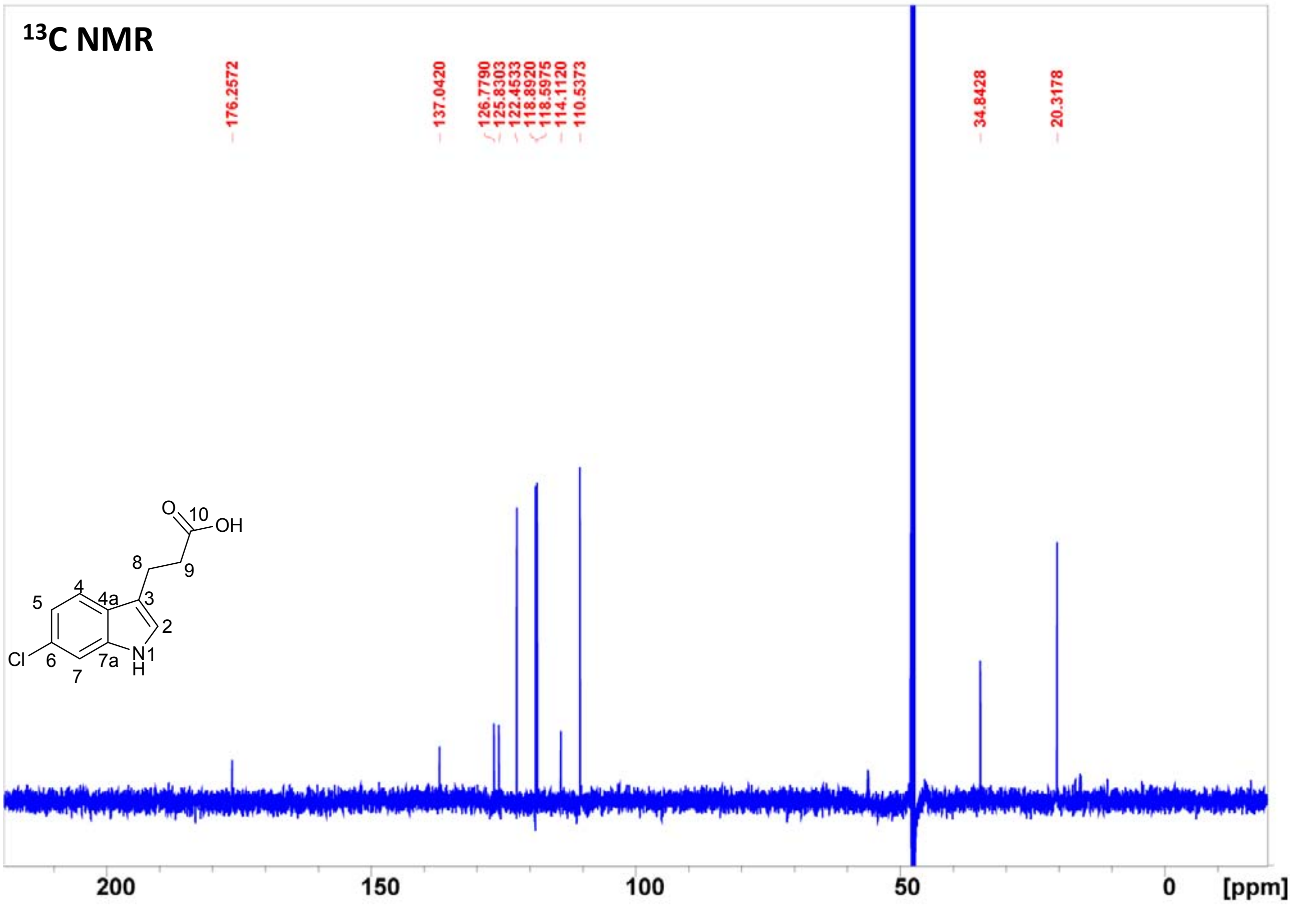

13C NMR
176.2572
137.0420
126.7790
125.8303
122.4533
118.8920
118.5975
114.1120
110.5373
34.8428
20.3178
O
10
OH
8
9
5
4
4a
3
2
Cl
6
7a
N
1
H
7
200
150
100
50
0
[ppm]

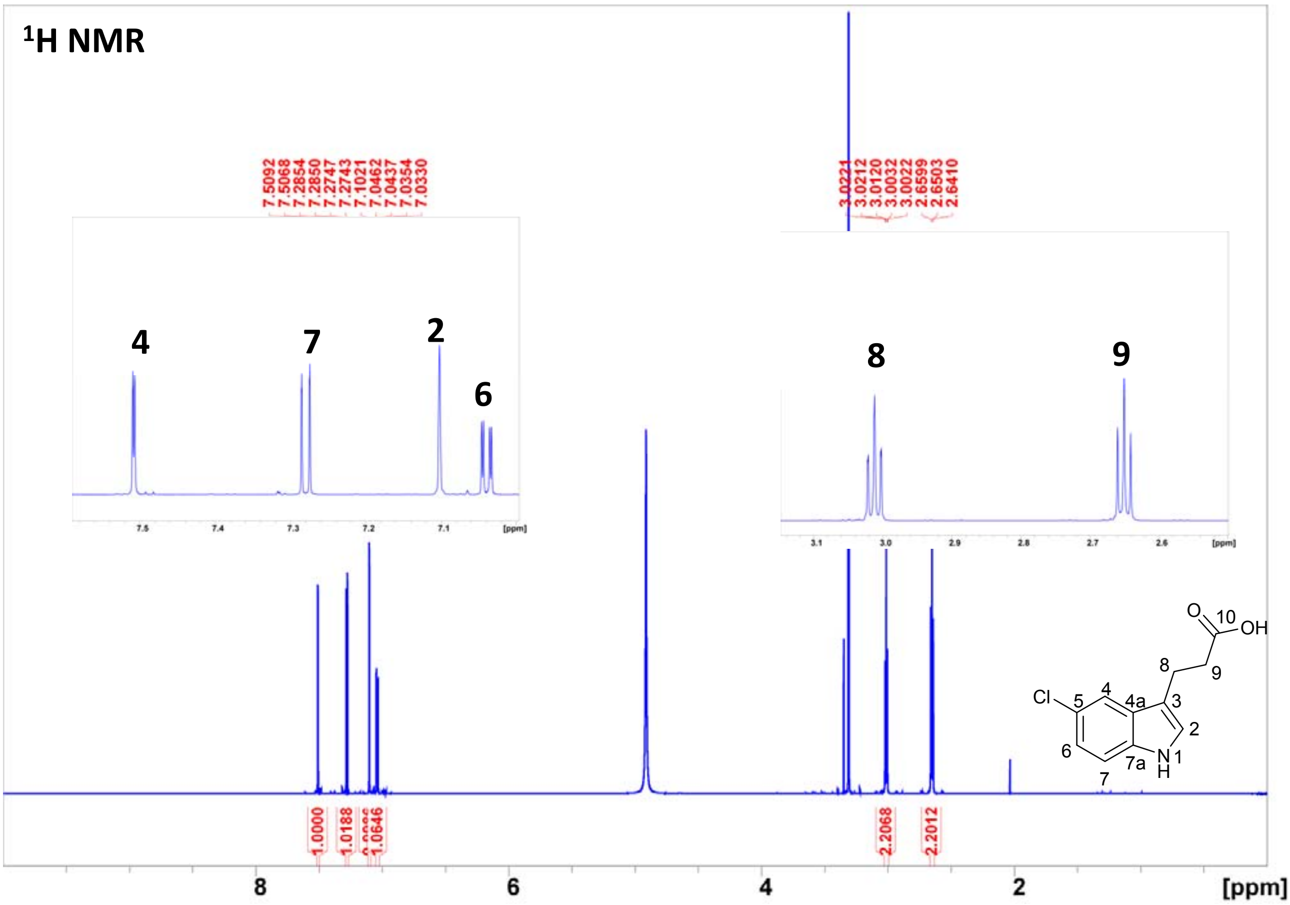

1H NMR
7.5092
7.5068
7.2854
7.2850
7.2747
7.2743
7.1021
7.0462
7.0437
7.0354
7.0330
3.0212
3.0120
3.0032
3.0022
2.6599
2.6503
2.6410
4
7
2
6
8
9
1.0000
1.0188
1.0646
2.2068
2.2012
O
10
OH
8
9
Cl
5
4
4a
3
2
6
7a
N
1
H
7
8
6
4
2
[ppm]

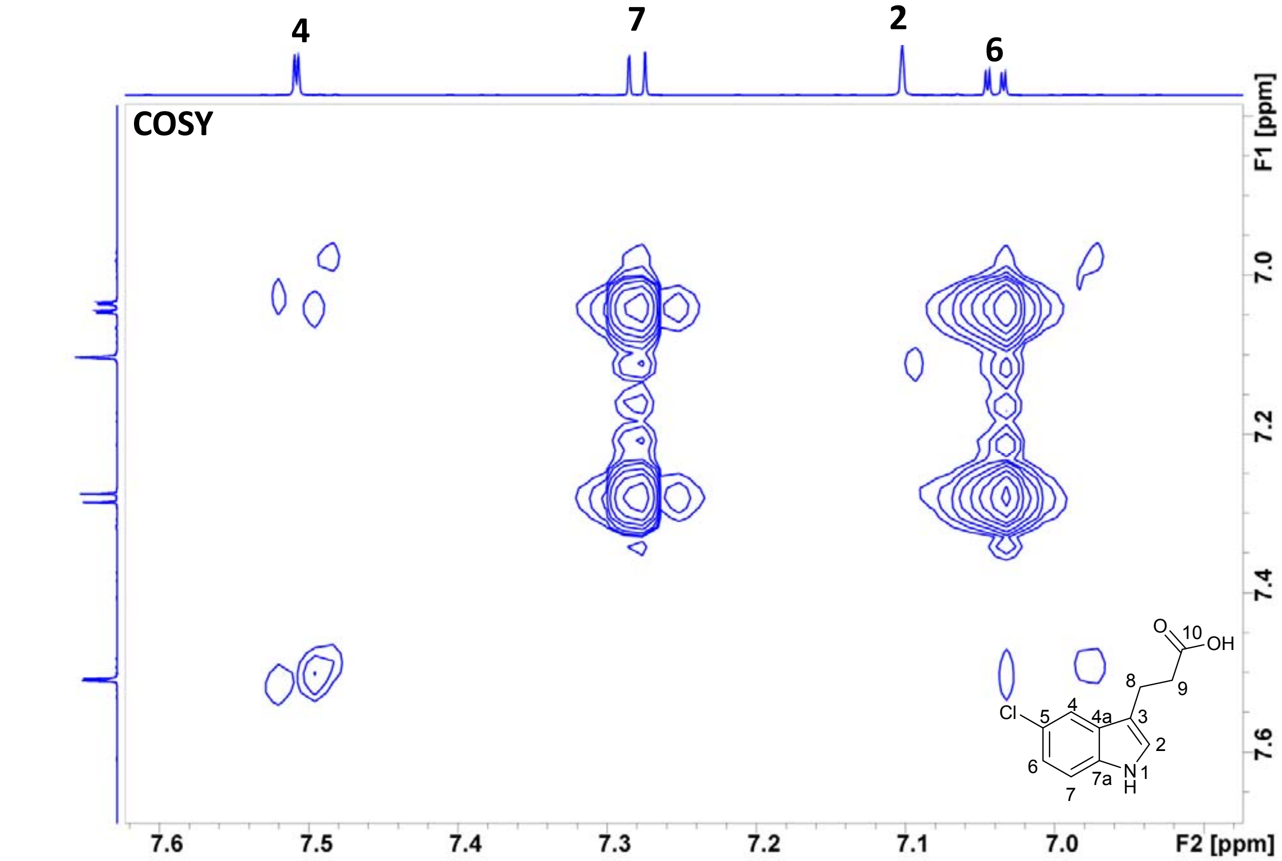
COSY
4
7
2
6
F1 [ppm]
F2 [ppm]
7.6
7.5
7.4
7.3
7.2
7.1
7.0
Cl
OH
O

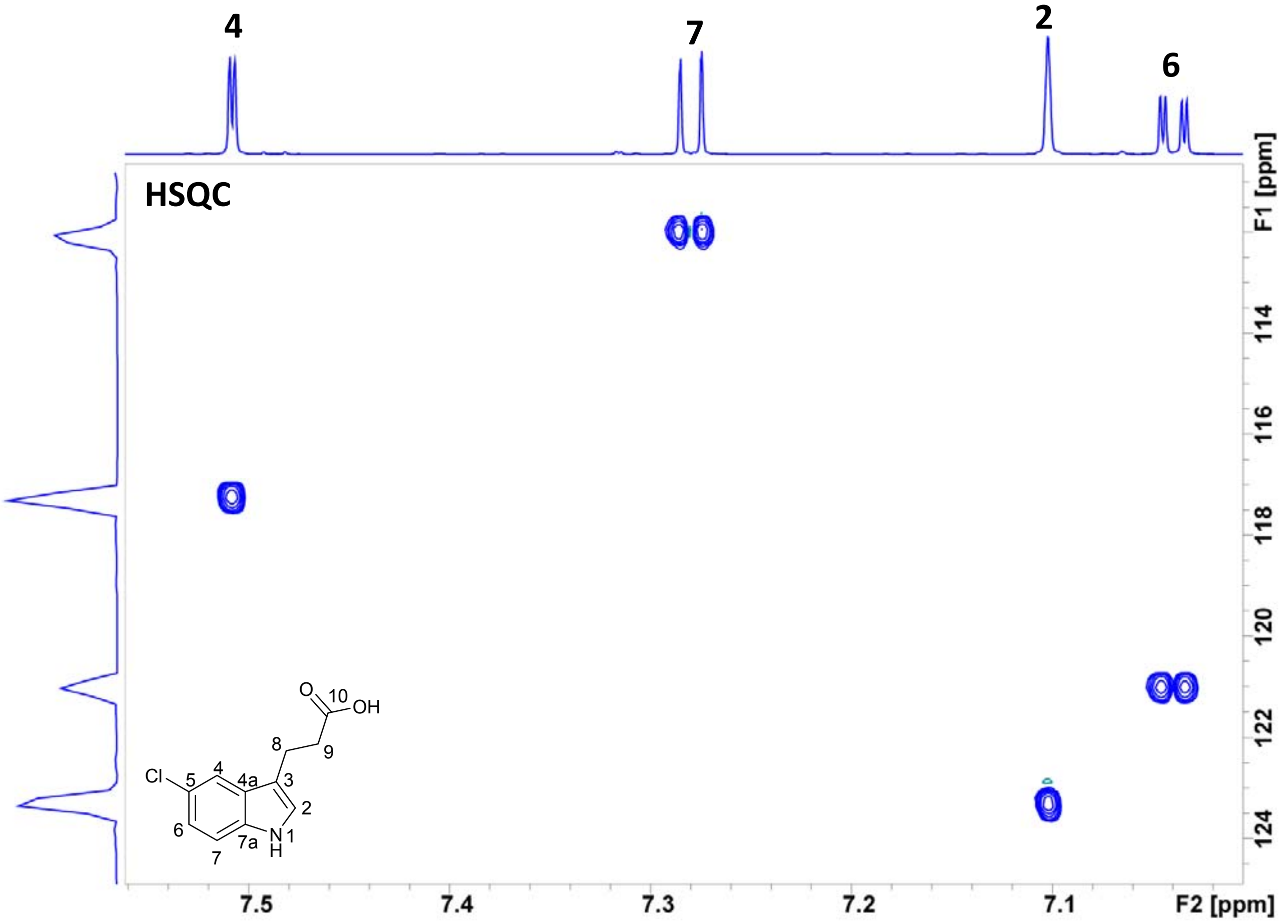
HSQC
4
7
2
6
F1 [ppm]
F2 [ppm]
114
116
118
120
122
124
7.5
7.4
7.3
7.2
7.1
Cl
OH
O

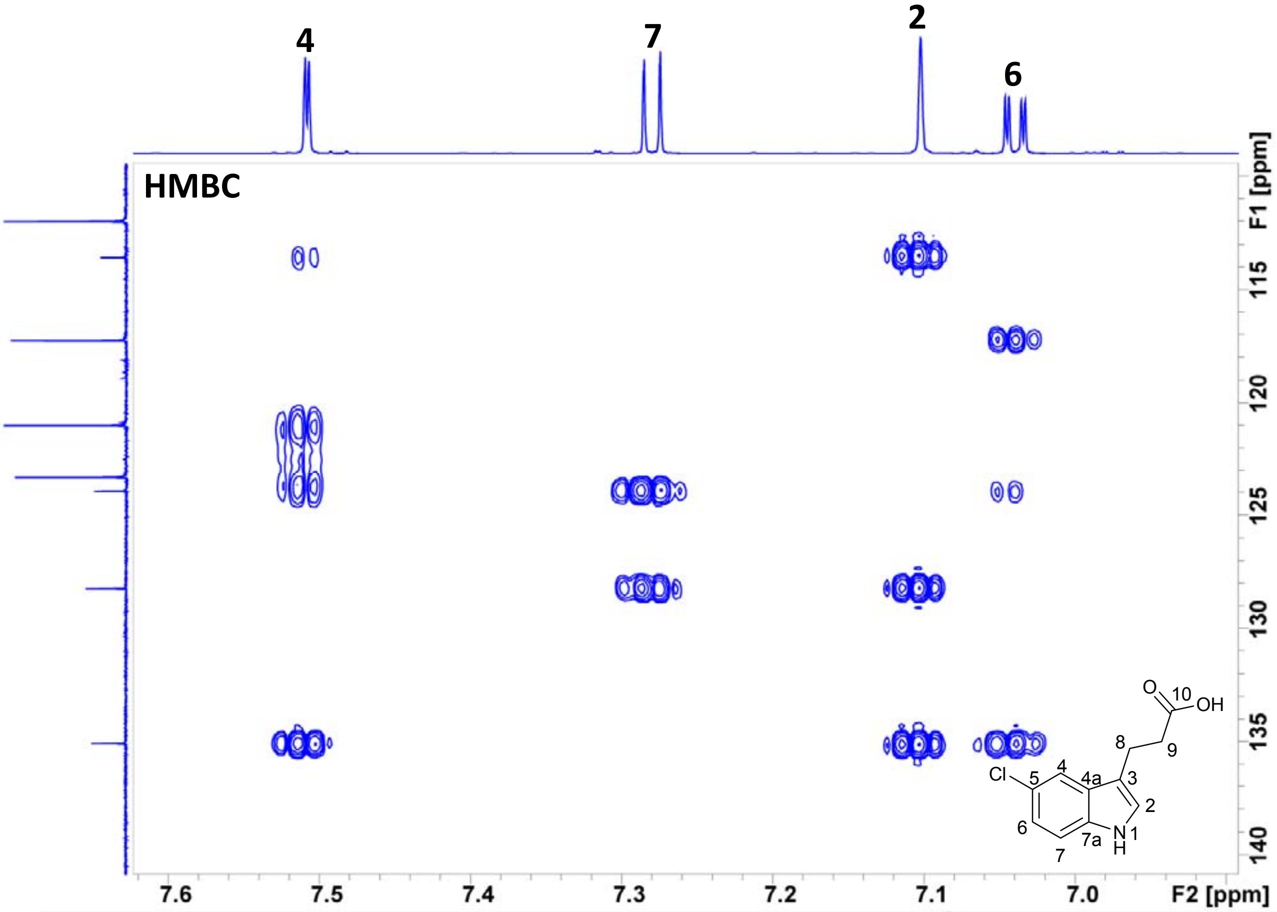
4
7
2
6
HMBC
F1 [ppm]
115
120
125
130
135
140
7.6
7.5
7.4
7.3
7.2
7.1
7.0
F2 [ppm]

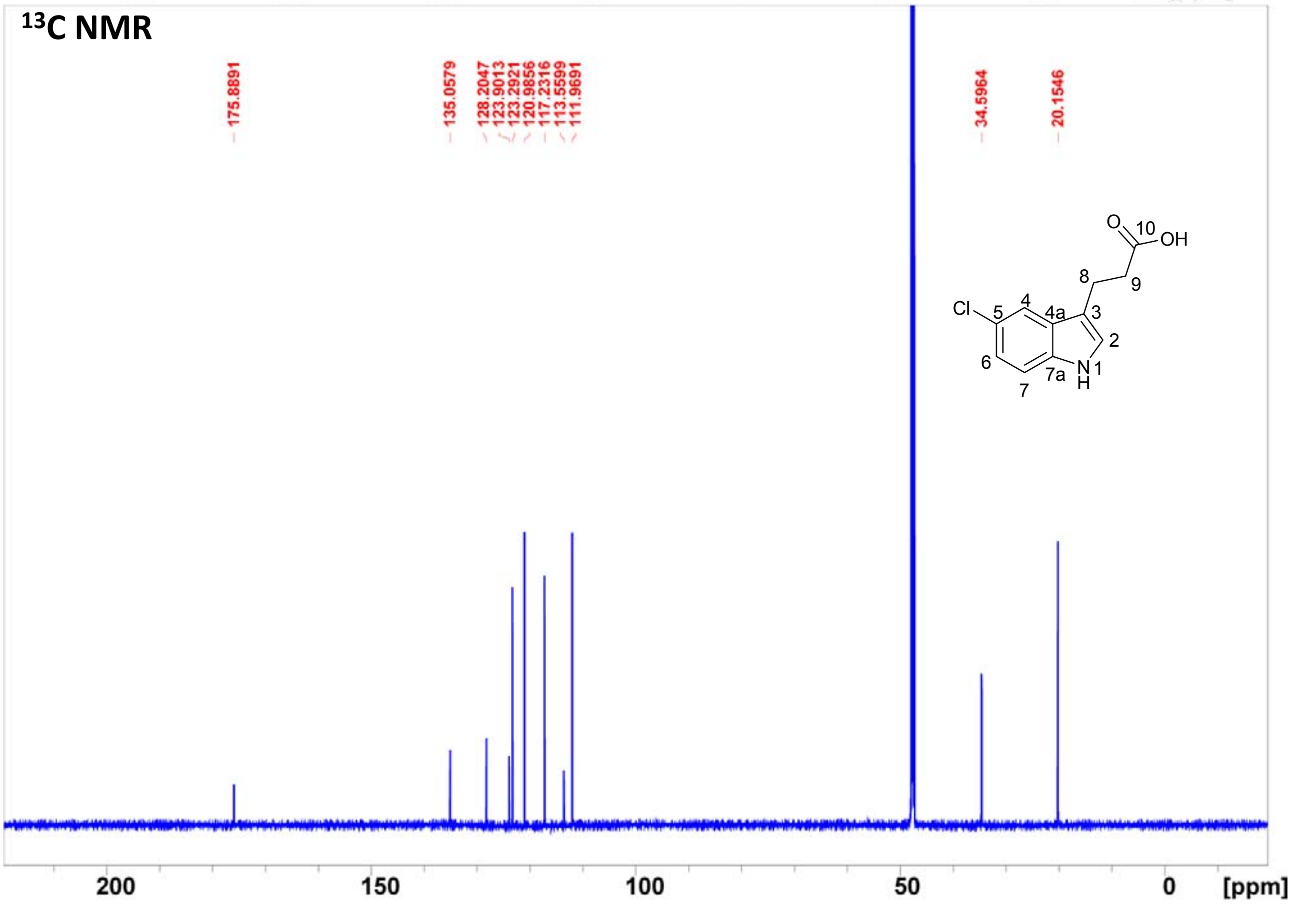
13C NMR
175.8891
135.0579
128.2047
123.9013
123.2921
120.9856
117.2316
113.5599
111.9691
34.5964
20.1546
200
150
100
50
0
[ppm]

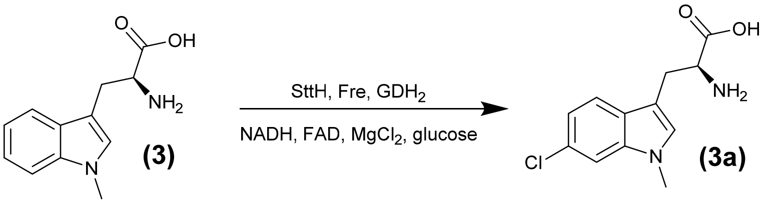

SttH, Fre, GDH$_2$
NADH, FAD, MgCl$_2$, glucose
(3)
(3a)

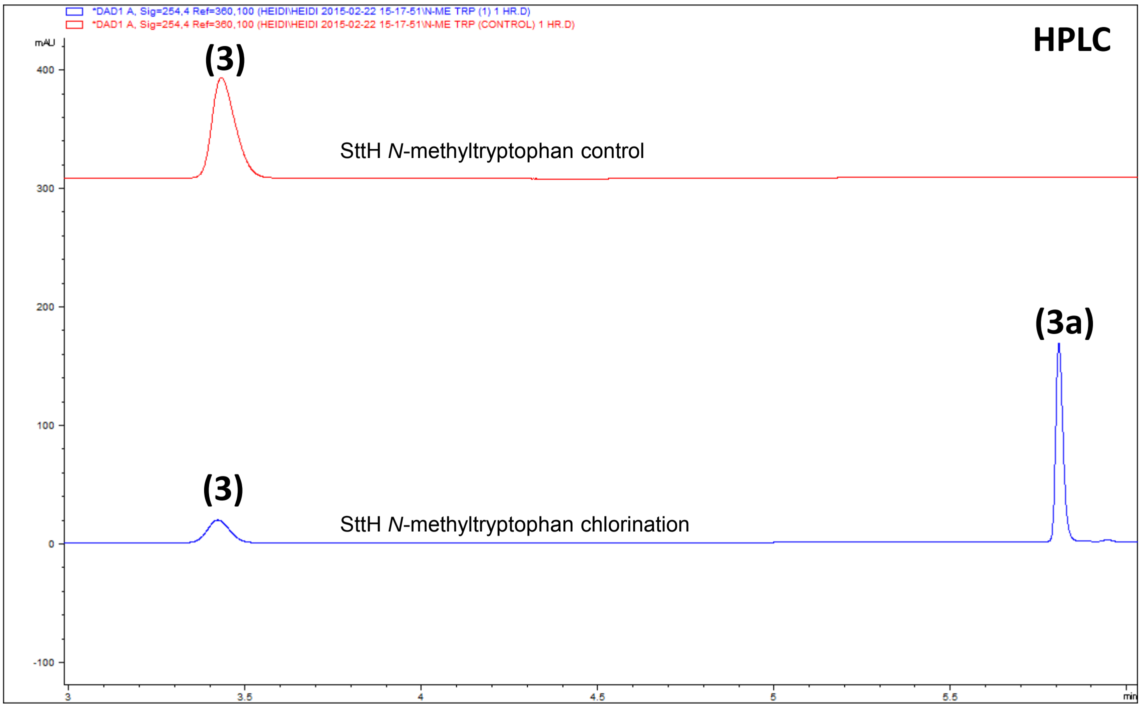

HPLC
(3)
SttH N-methyltryptophan control
(3a)
(3)
SttH N-methyltryptophan chlorination
mAU
min

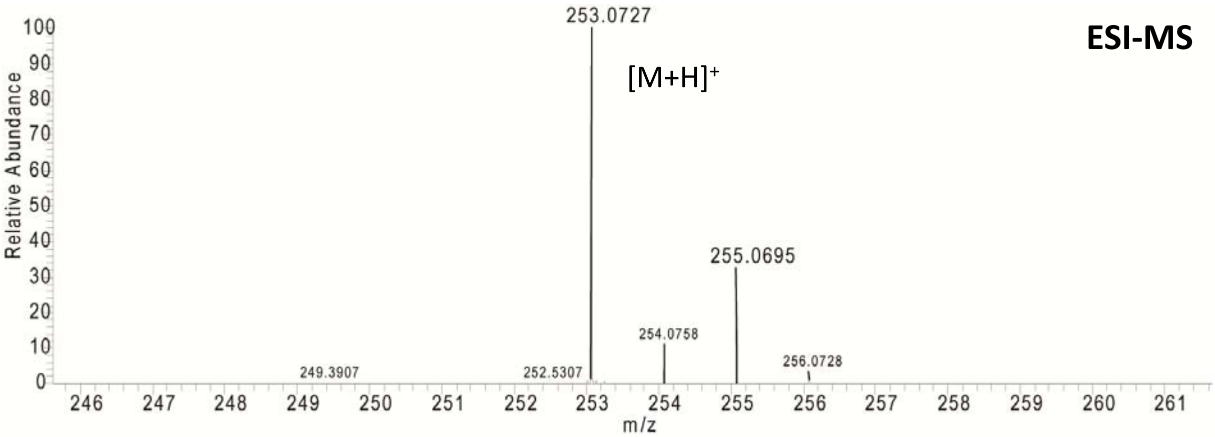

ESI-MS
253.0727
[M+H]+
255.0695
254.0758
256.0728
249.3907
252.5307
Relative Abundance
m/z

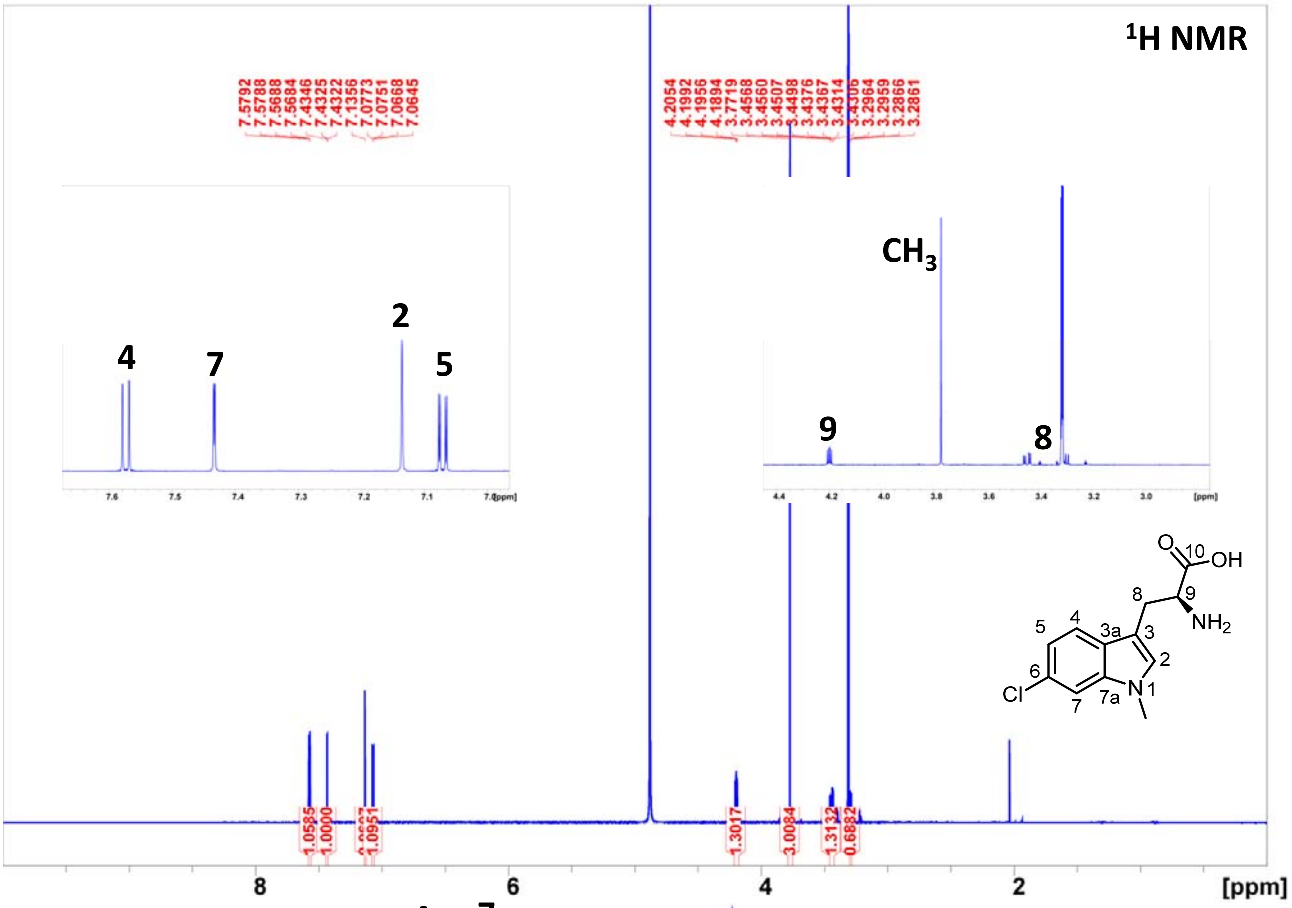

1H NMR
7.5792
7.5788
7.5688
7.5684
7.4346
7.4325
7.4322
7.1356
7.0773
7.0751
7.0668
7.0645
4.2054
4.1992
4.1956
4.1894
3.7719
3.4568
3.4560
3.4507
3.4498
3.4376
3.4367
3.4314
3.4306
3.2964
3.2959
3.2866
3.2861
4
7
2
5
CH3
9
8
1.0535
1.0000
1.0951
1.3017
3.0084
1.3132
0.6832
8
6
4
2
[ppm]
O
10
OH
8
9
NH2
5
4
3a
3
2
6
Cl
7
7a
N
1

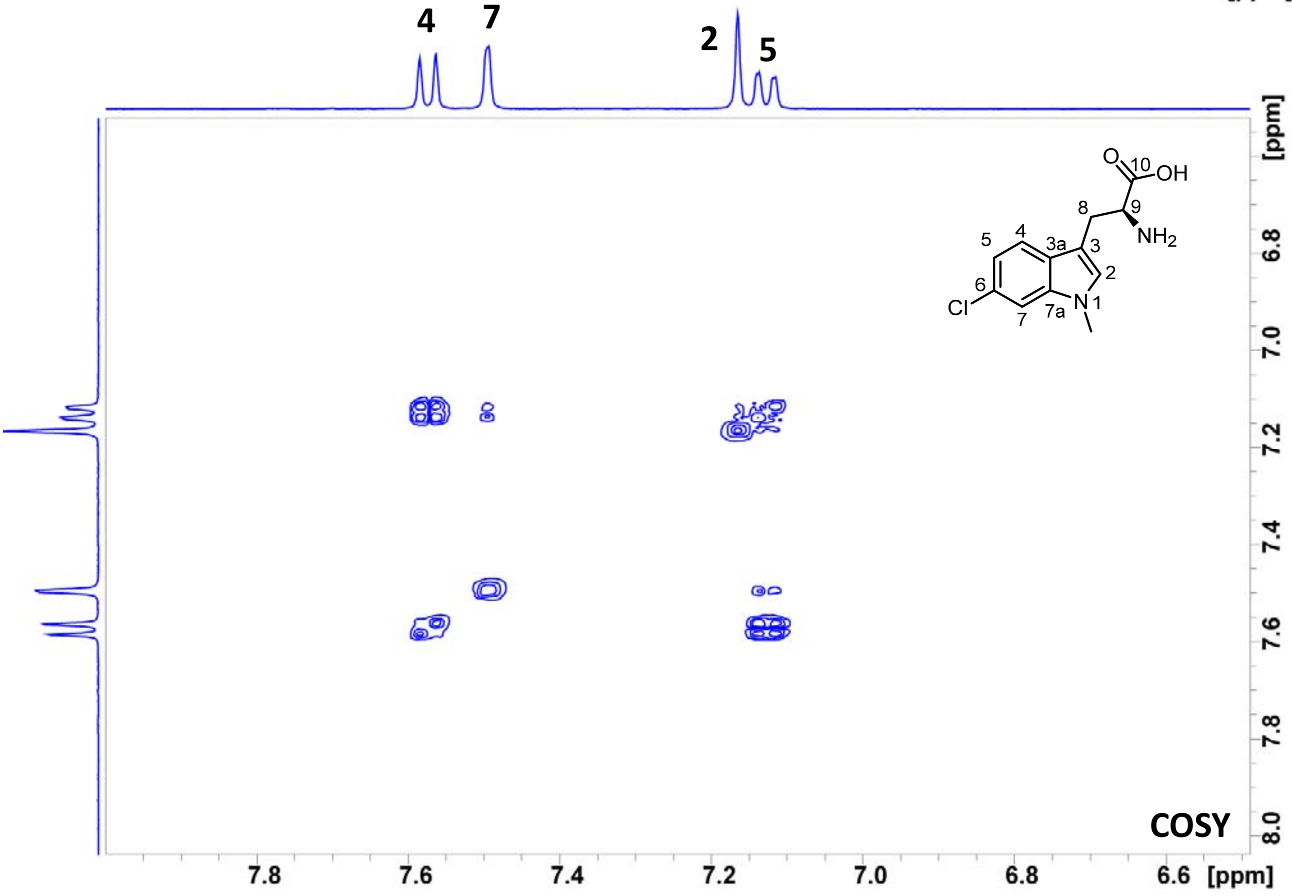

4
7
2
5
O
10
OH
8
9
NH2
5
4
3a
3
2
6
Cl
7
7a
N
1
COSY
7.8
7.6
7.4
7.2
7.0
6.8
6.6
[ppm]

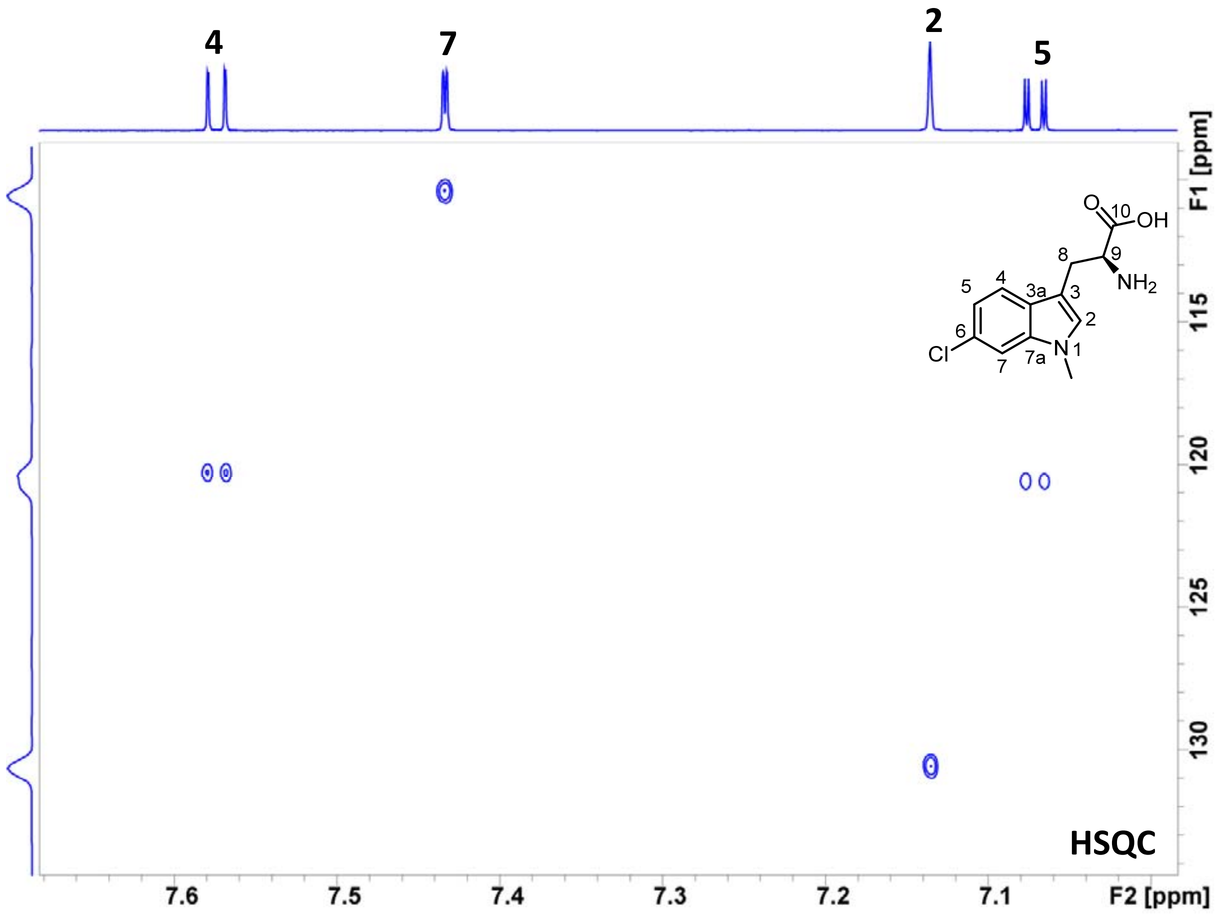
4
7
2
5
F1 [ppm]
115
120
125
130
HSQC
7.6
7.5
7.4
7.3
7.2
7.1
F2 [ppm]

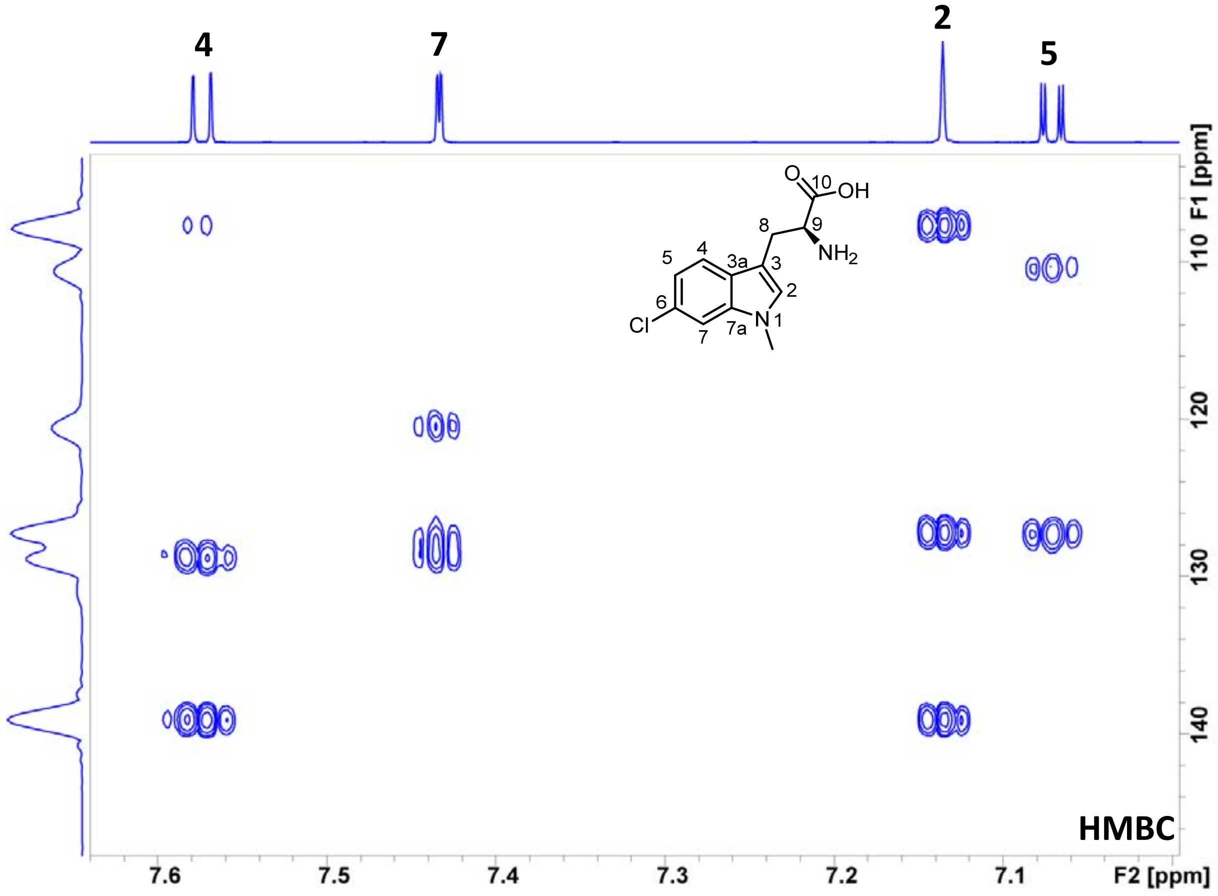
4
7
2
5
F1 [ppm]
110
120
130
140
HMBC
7.6
7.5
7.4
7.3
7.2
7.1
F2 [ppm]

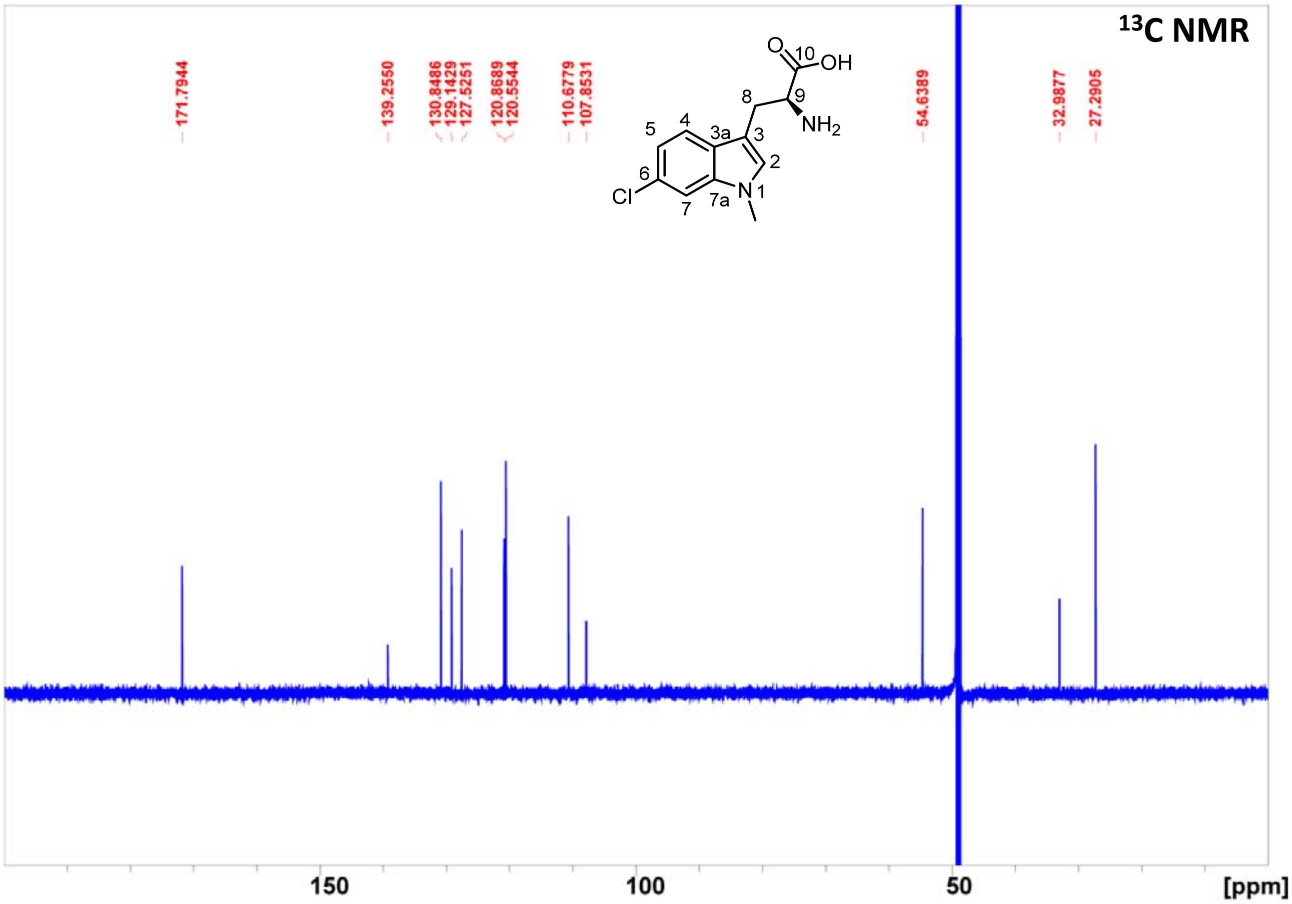

13C NMR
171.7944
139.2550
130.8486
129.1429
127.5251
120.8689
120.5544
110.6779
107.8531
54.6389
32.9877
27.2905
150
100
50
[ppm]

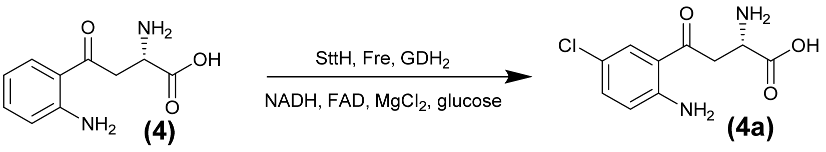
(4)
SttH, Fre, GDH2
NADH, FAD, MgCl2, glucose
(4a)

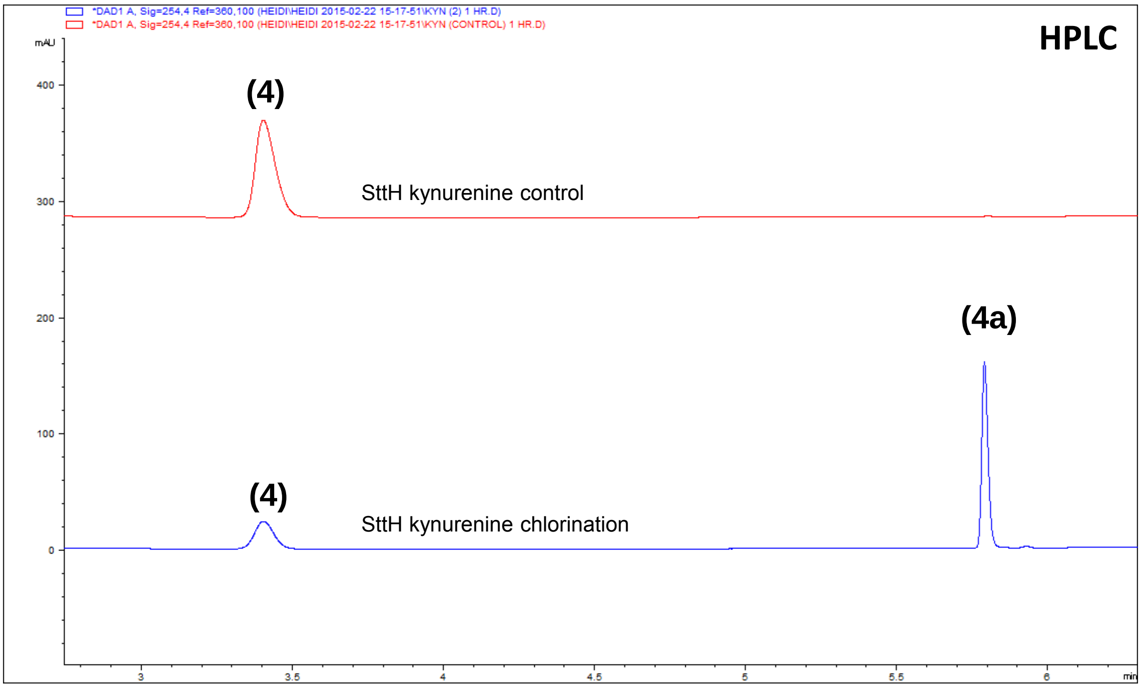
*DAD1 A, Sig=254,4 Ref=360,100 (HEIDI\HEIDI 2015-02-22 15-17-51\KYN (2) 1 HR.D)
*DAD1 A, Sig=254,4 Ref=360,100 (HEIDI\HEIDI 2015-02-22 15-17-51\KYN (CONTROL) 1 HR.D)
HPLC
mAU
(4)
SttH kynurenine control
(4a)
(4)
SttH kynurenine chlorination
min

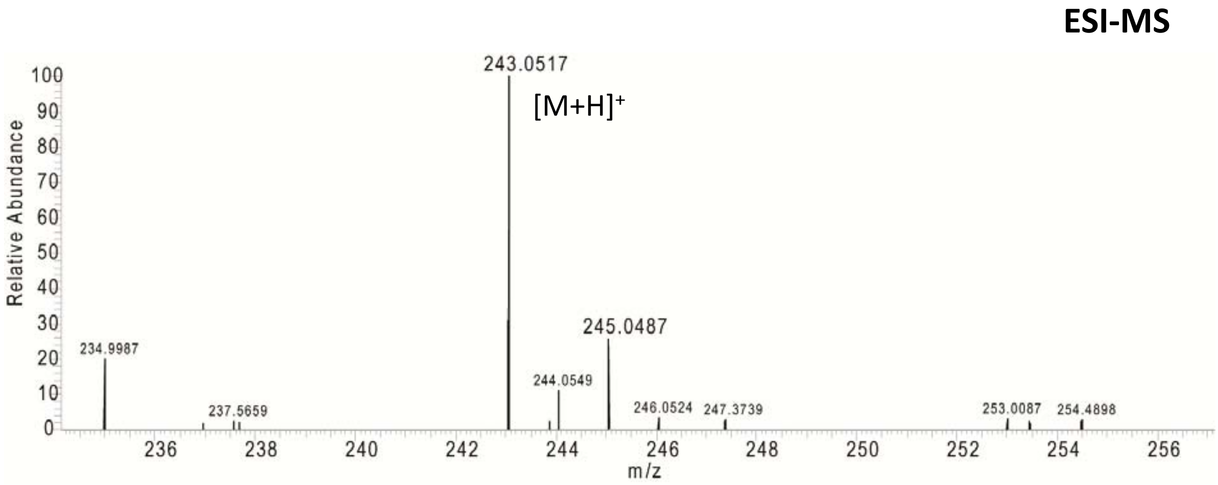
ESI-MS
243.0517
[M+H]+
Relative Abundance
234.9987
237.5659
244.0549
245.0487
246.0524
247.3739
253.0087
254.4898
m/z

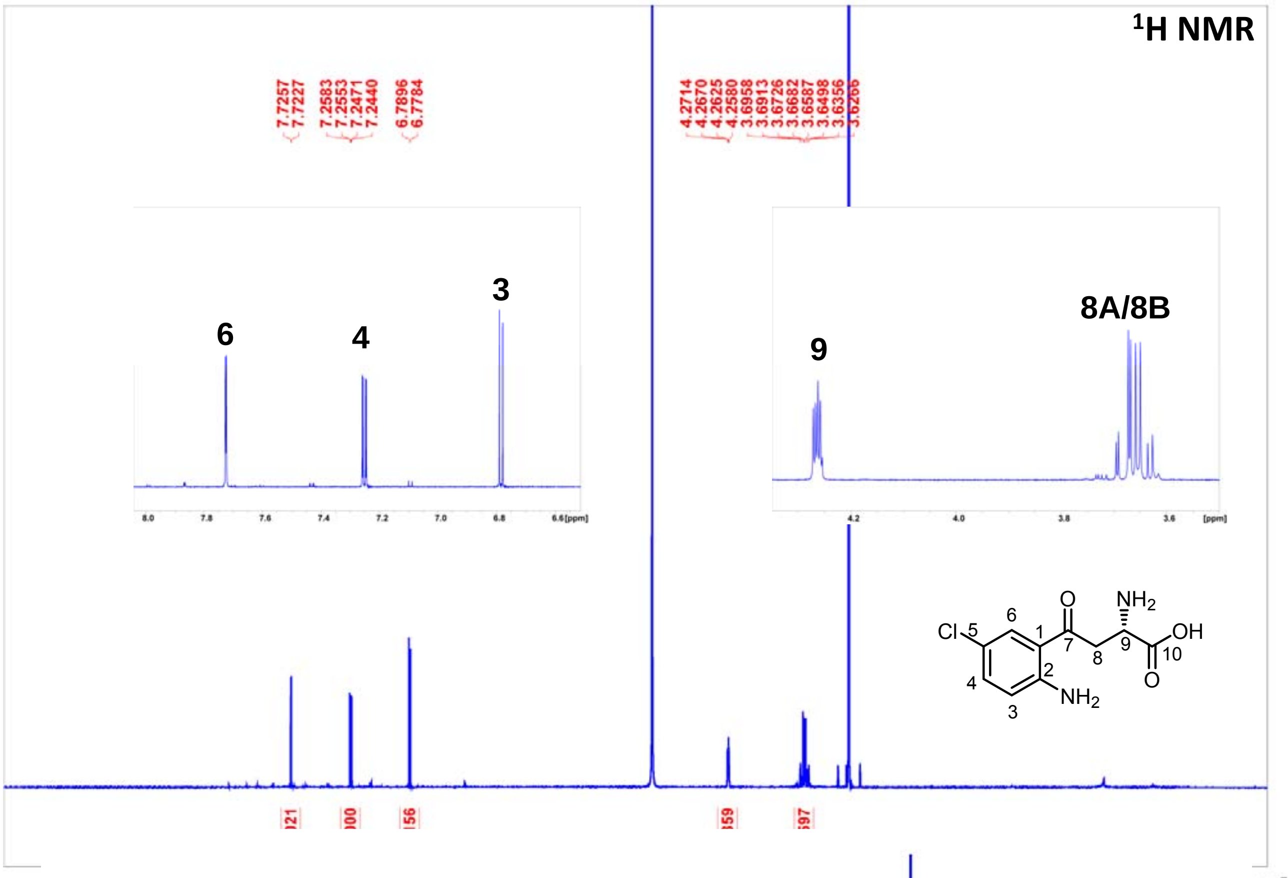

1H NMR
7.7257
7.7227
7.2583
7.2553
7.2471
7.2440
6.7896
6.7784
4.2714
4.2670
4.2625
4.2580
3.6958
3.6913
3.6726
3.6682
3.6587
3.6498
3.6356
6
4
3
9
8A/8B
Cl
5
6
1
7
O
NH2
9
8
OH
10
O
2
4
3
NH2

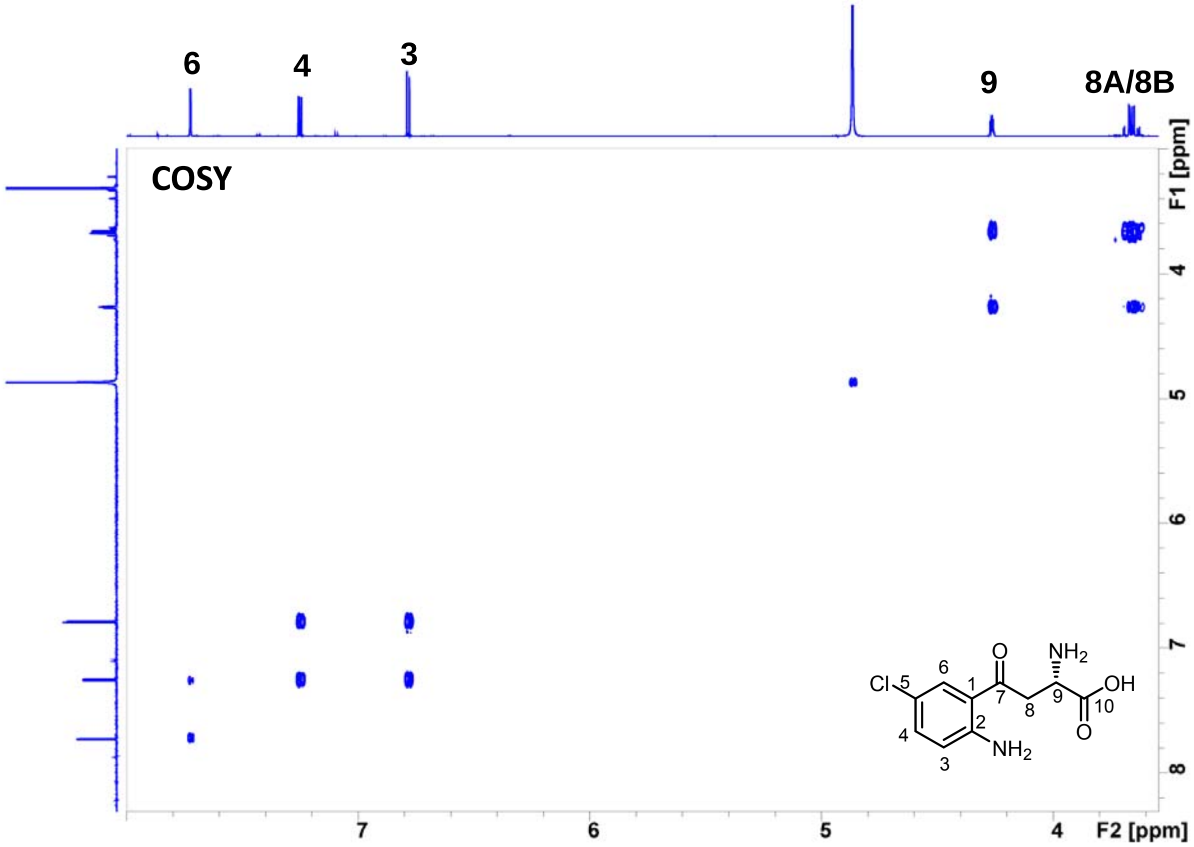

6
4
3
9
8A/8B
COSY
F1 [ppm]
F2 [ppm]
Cl
5
6
1
7
O
NH2
9
8
OH
10
O
2
4
3
NH2

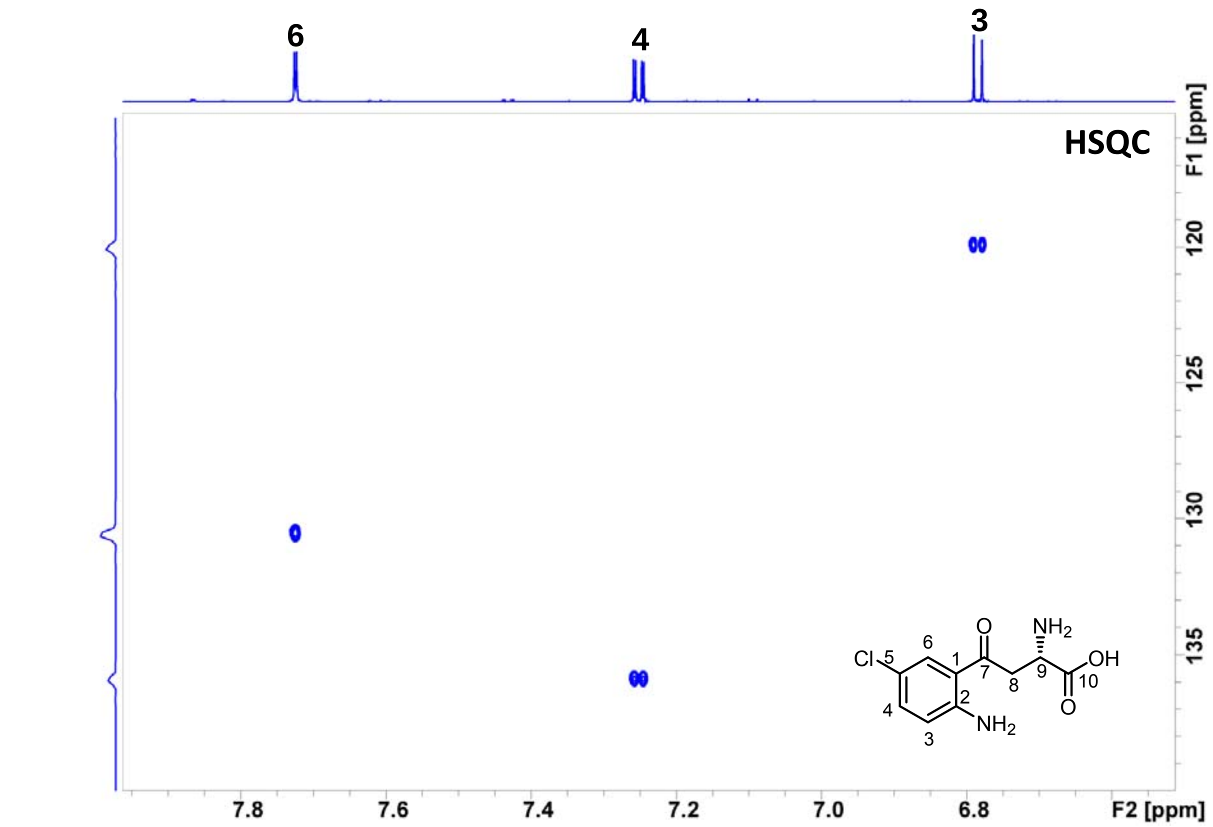
6
4
3
HSQC
F1 [ppm]
120
125
130
135
7.8
7.6
7.4
7.2
7.0
6.8
F2 [ppm]
Cl
5
6
1
O
7
8
9
NH2
OH
10
O
2
NH2
4
3

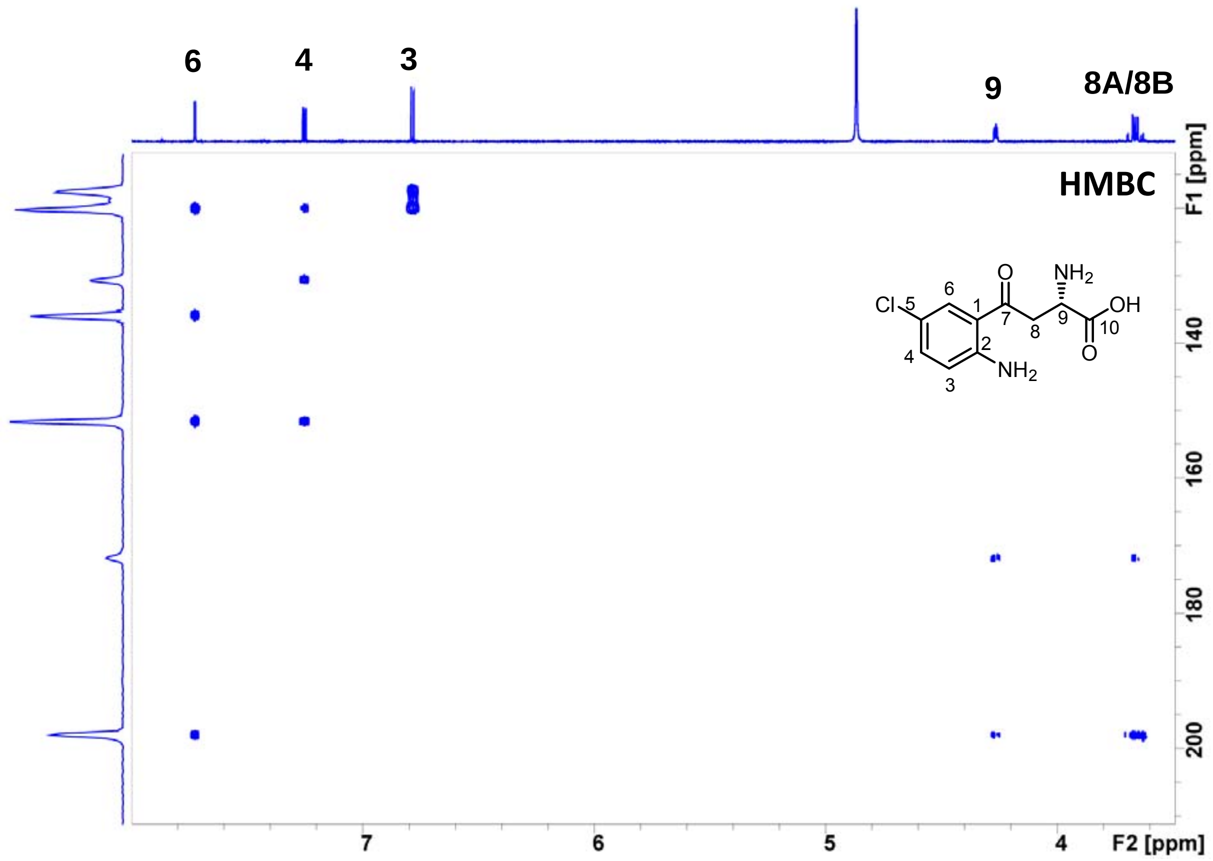
6
4
3
9
8A/8B
HMBC
F1 [ppm]
140
160
180
200
7
6
5
4
F2 [ppm]
Cl
5
6
1
O
7
8
9
NH2
OH
10
O
2
NH2
4
3

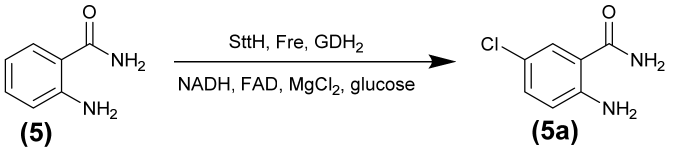
O
NH2
NH2
(5)
SttH, Fre, GDH2
NADH, FAD, MgCl2, glucose
Cl
O
NH2
NH2
(5a)

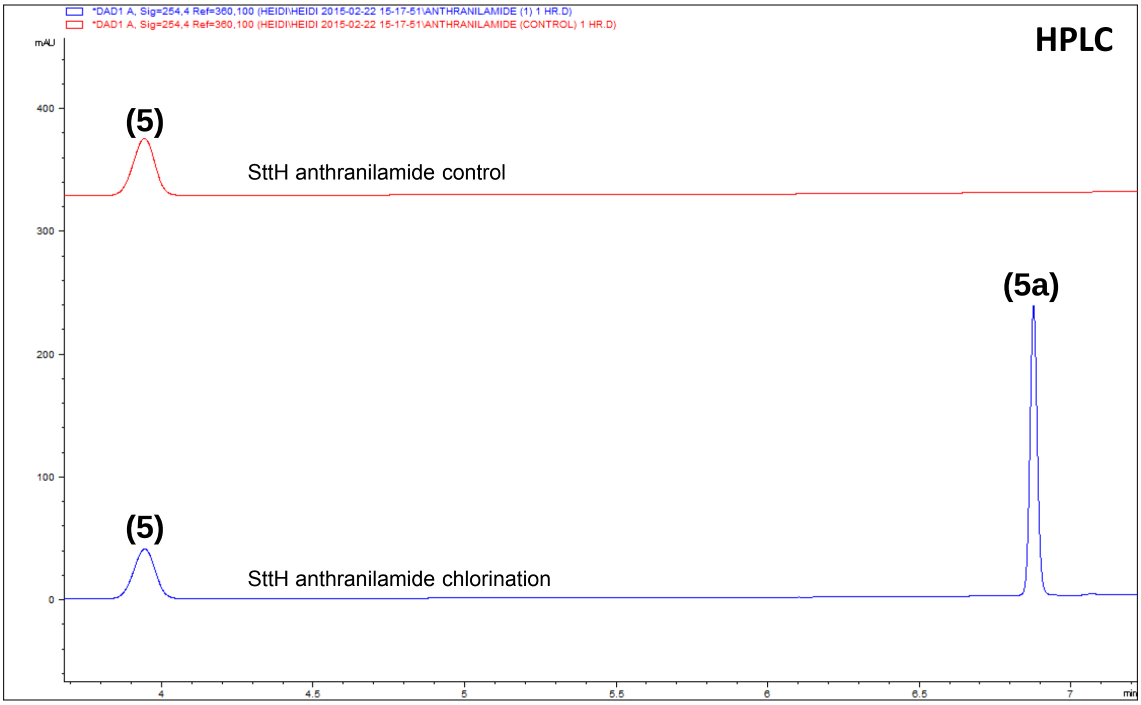
*DAD1 A, Sig=254,4 Ref=360,100 (HEIDI\HEIDI 2015-02-22 15-17-51\ANTHRANILAMIDE (1) 1 HR.D)
*DAD1 A, Sig=254,4 Ref=360,100 (HEIDI\HEIDI 2015-02-22 15-17-51\ANTHRANILAMIDE (CONTROL) 1 HR.D)
HPLC
mAU
400
300
200
100
0
(5)
SttH anthranilamide control
(5a)
(5)
SttH anthranilamide chlorination
4
4.5
5
5.5
6
6.5
7
min

ESI-MS

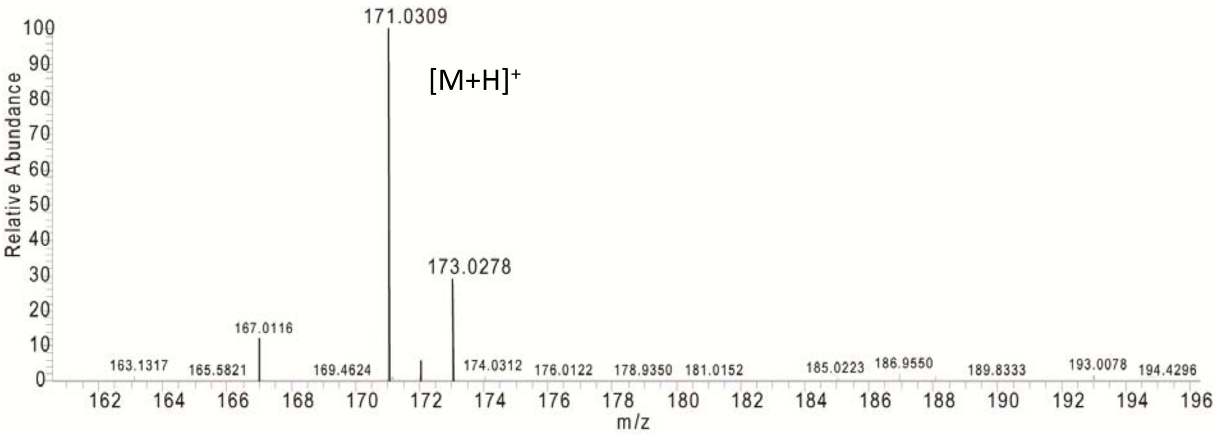
Relative Abundance
171.0309
[M+H]+
173.0278
167.0116
163.1317
165.5821
169.4624
174.0312
176.0122
178.9350
181.0152
185.0223
186.9550
189.8333
193.0078
194.4296
m/z

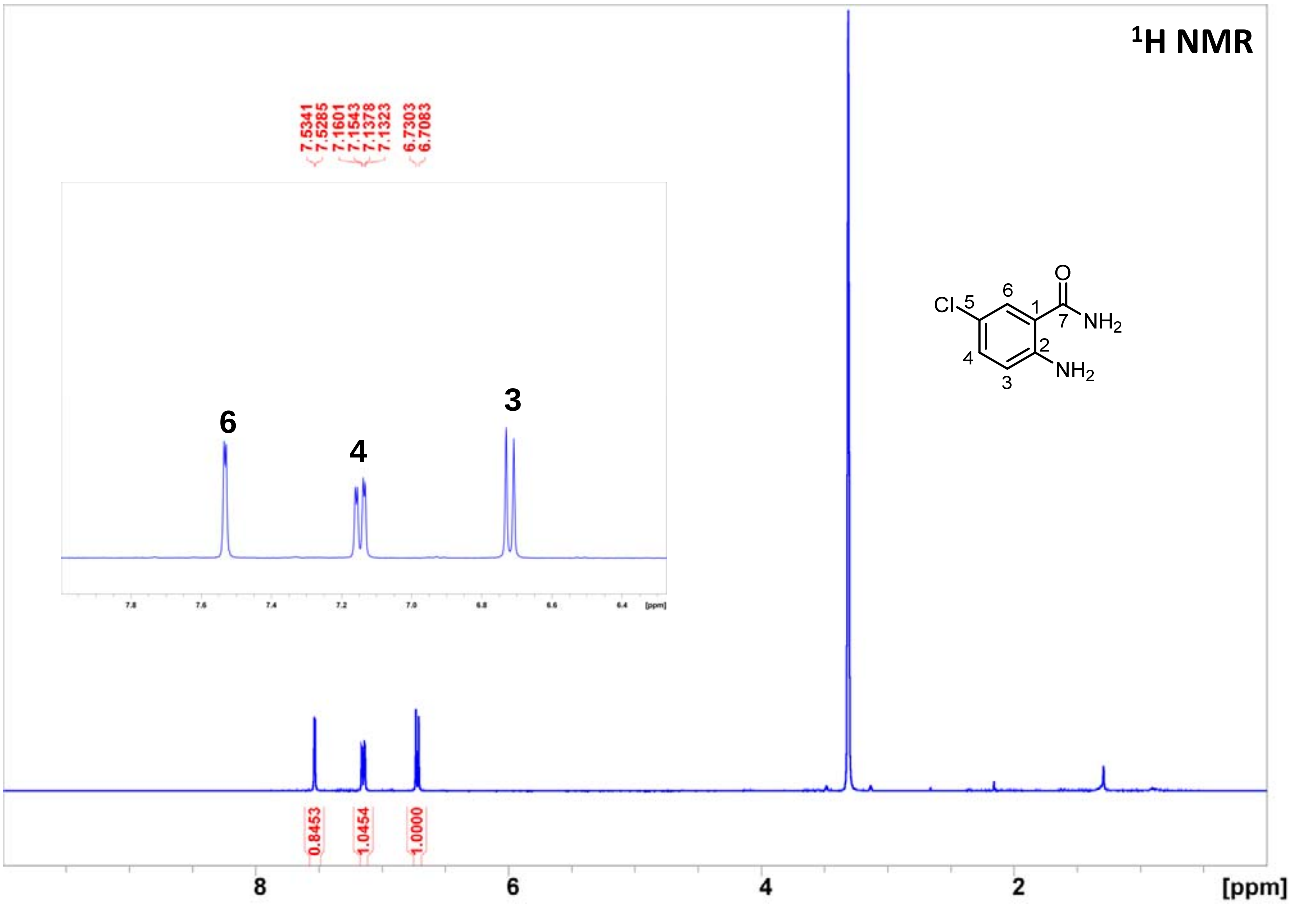

^{1}H NMR
7.5341
7.5285
7.1601
7.1543
7.1378
7.1323
6.7303
6.7083
6
4
3
Cl
5
6
1
7
O
NH2
2
4
3
NH2
0.8453
1.0454
1.0000
8
6
4
2
[ppm]

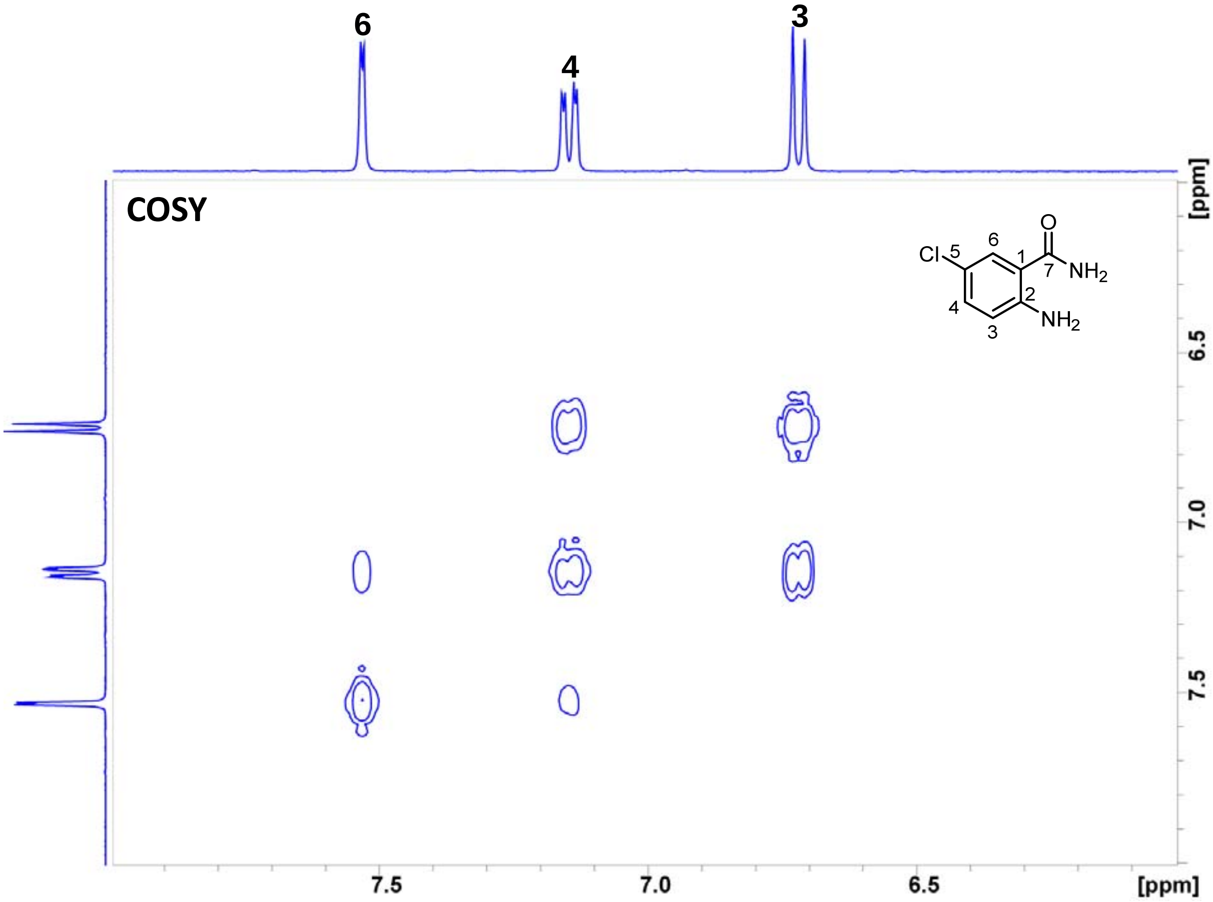

6
4
3
COSY
Cl
5
6
1
7
O
NH2
2
4
3
NH2
[ppm]
6.5
7.0
7.5
7.5
7.0
6.5
[ppm]

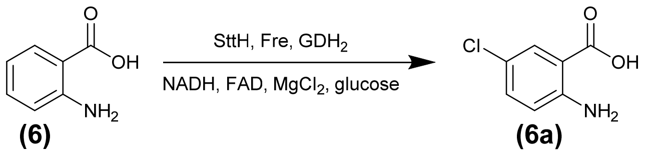
SttH, Fre, GDH2
NADH, FAD, MgCl2, glucose
OH
NH2
(6)
Cl
(6a)

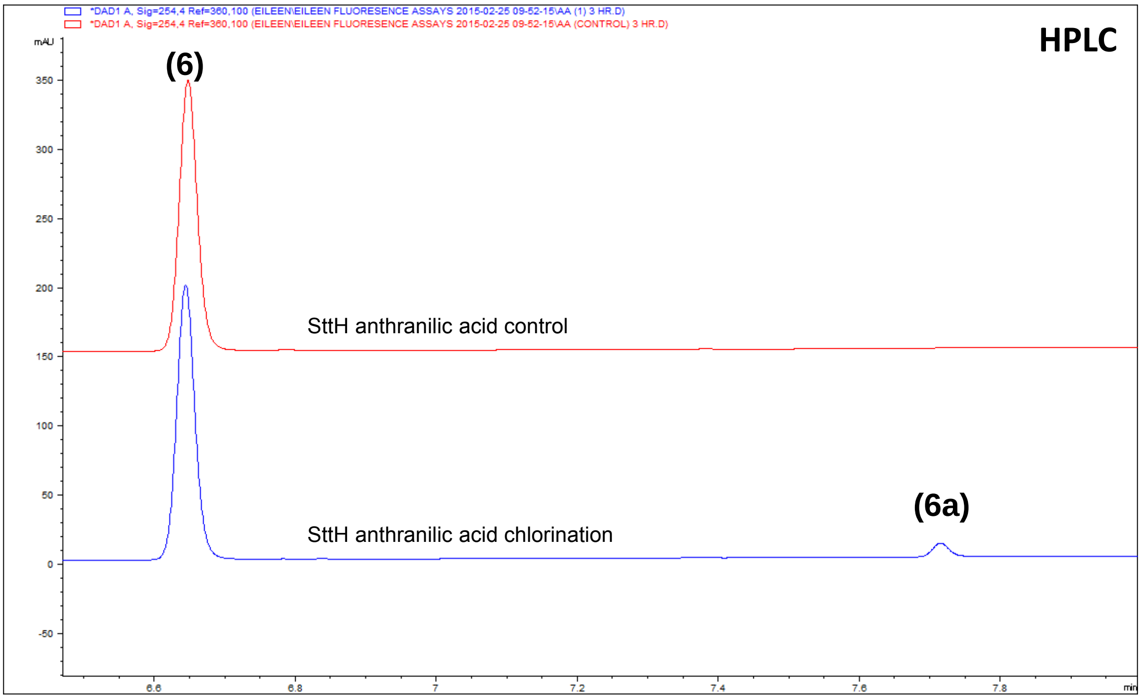
HPLC
(6)
SttH anthranilic acid control
SttH anthranilic acid chlorination
(6a)
mAU
min

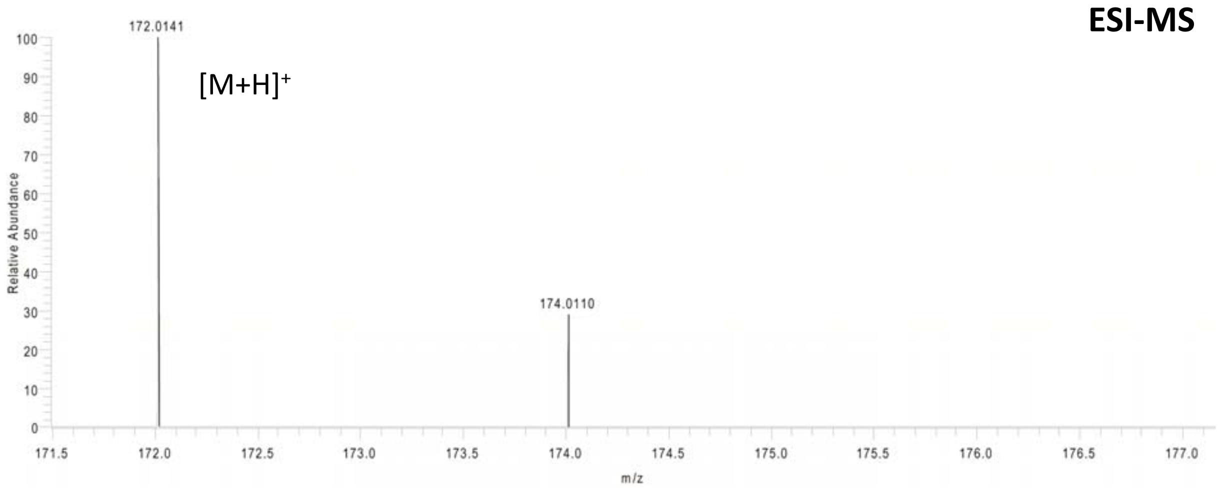
ESI-MS
172.0141
[M+H]+
174.0110
Relative Abundance
m/z

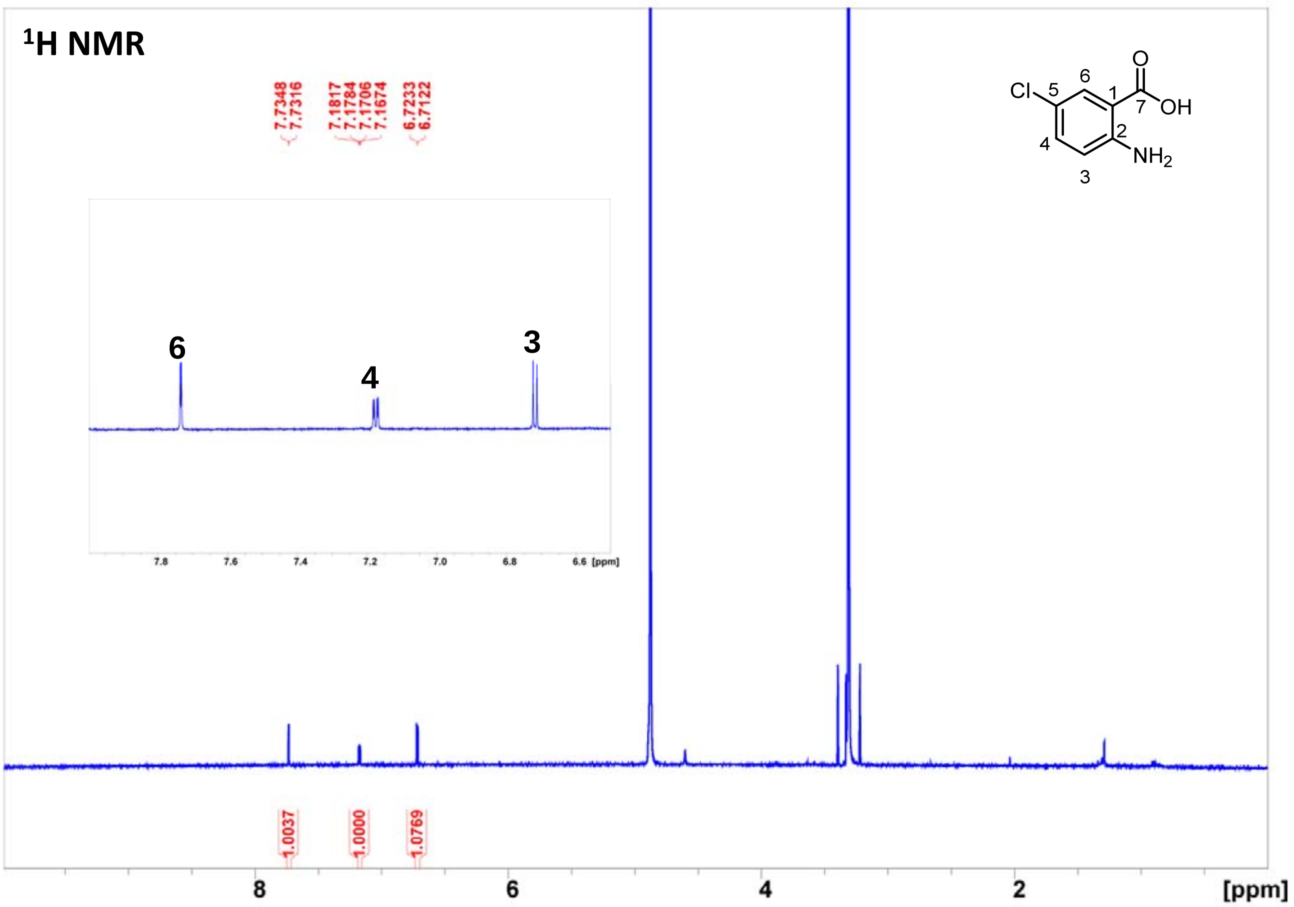

^{1}H NMR
7.7348
7.7316
7.1817
7.1784
7.1706
7.1674
6.7233
6.7122
6
4
3
1.0037
1.0000
1.0769
8
6
4
2
[ppm]
Cl
5
6
1
7
O
OH
2
NH2
4
3

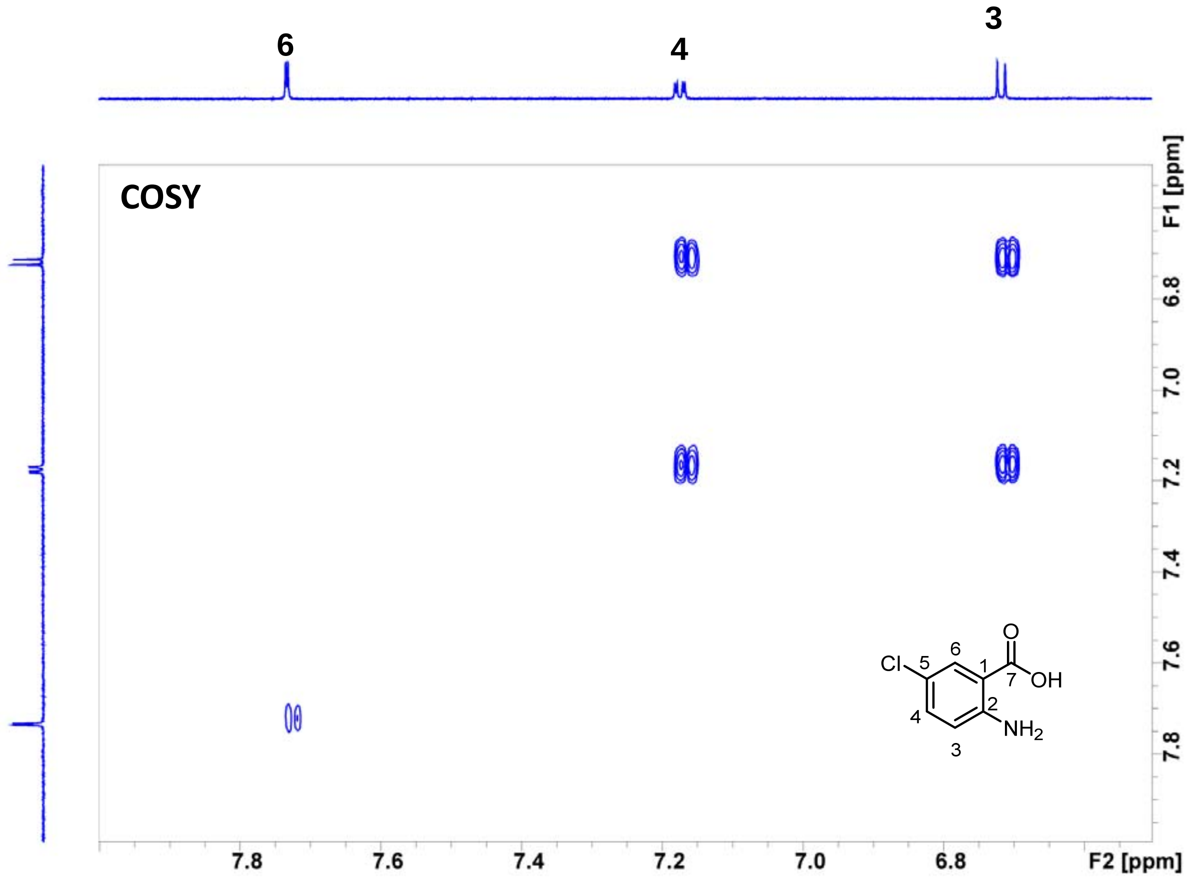

6
4
3
COSY
F1 [ppm]
6.8
7.0
7.2
7.4
7.6
7.8
F2 [ppm]
Cl
5
6
1
7
O
OH
2
NH2
4
3

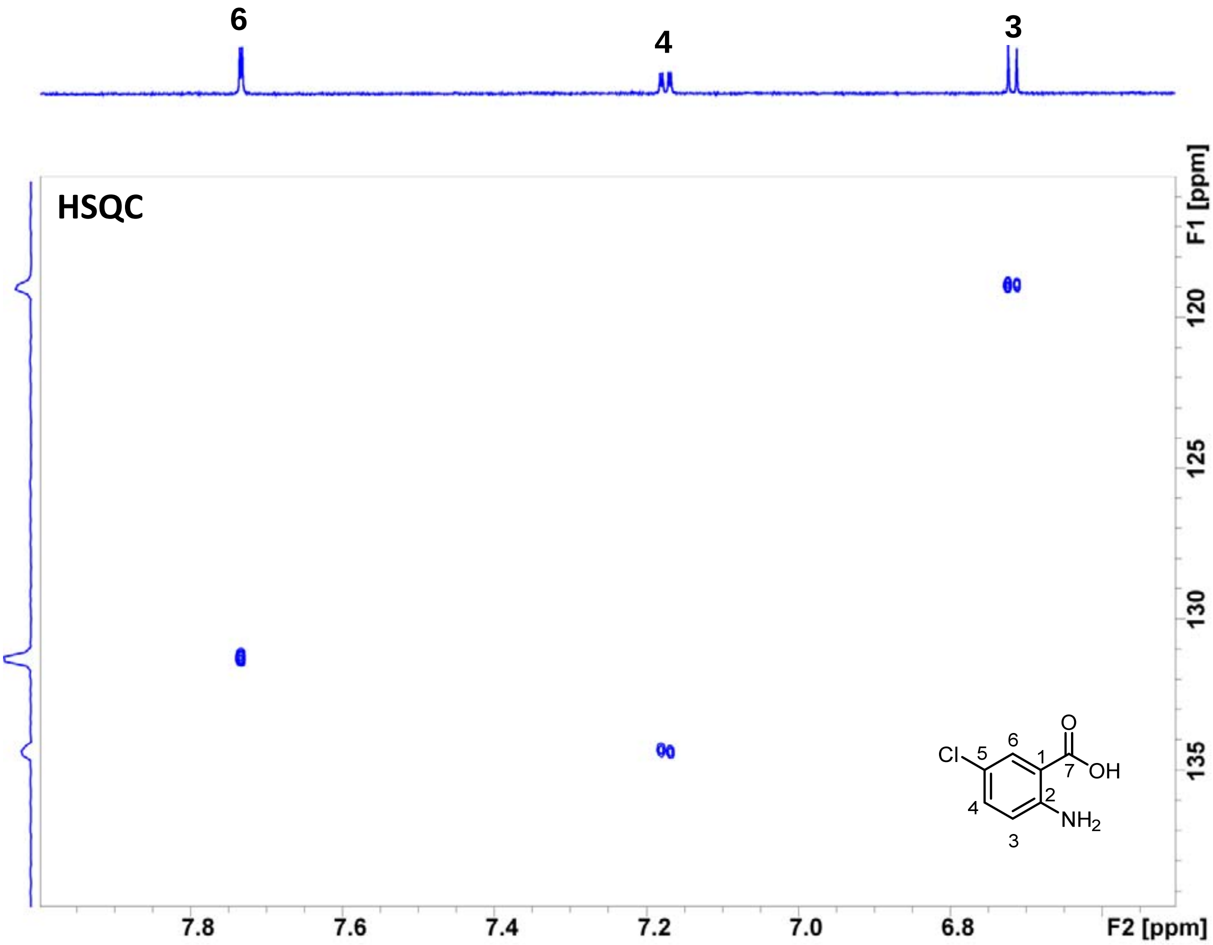

6
4
3
HSQC
F1 [ppm]
120
125
130
135
7.8
7.6
7.4
7.2
7.0
6.8
F2 [ppm]
Cl
5
6
1
O
7
OH
2
NH2
4
3

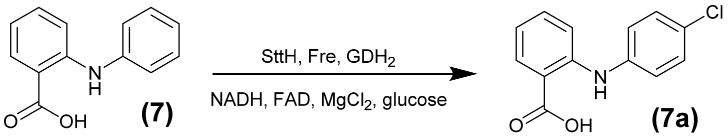
SttH, Fre, GDH2
NADH, FAD, MgCl2, glucose
(7)
(7a)
Cl
N
H
O
OH

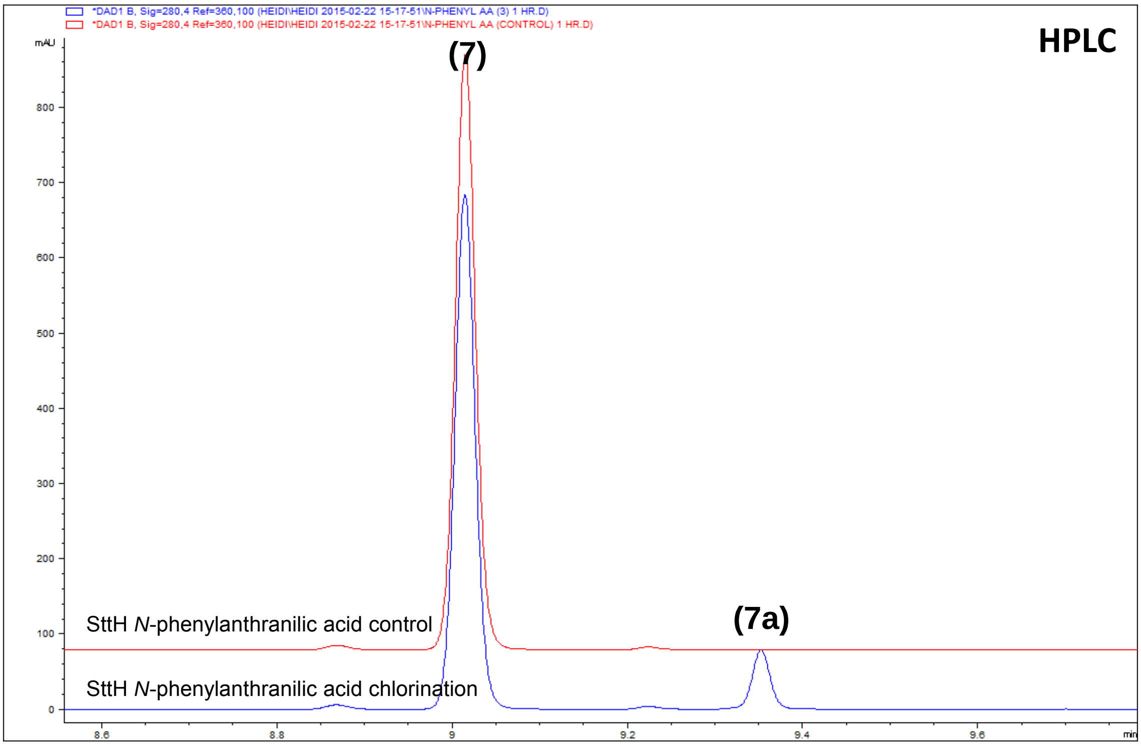
*DAD1 B, Sig=280,4 Ref=360,100 (HEIDI\HEIDI 2015-02-22 15-17-51\N-PHENYL AA (3) 1 HR.D)
*DAD1 B, Sig=280,4 Ref=360,100 (HEIDI\HEIDI 2015-02-22 15-17-51\N-PHENYL AA (CONTROL) 1 HR.D)
HPLC
mAU
(7)
(7a)
SttH N-phenylanthranilic acid control
SttH N-phenylanthranilic acid chlorination
min

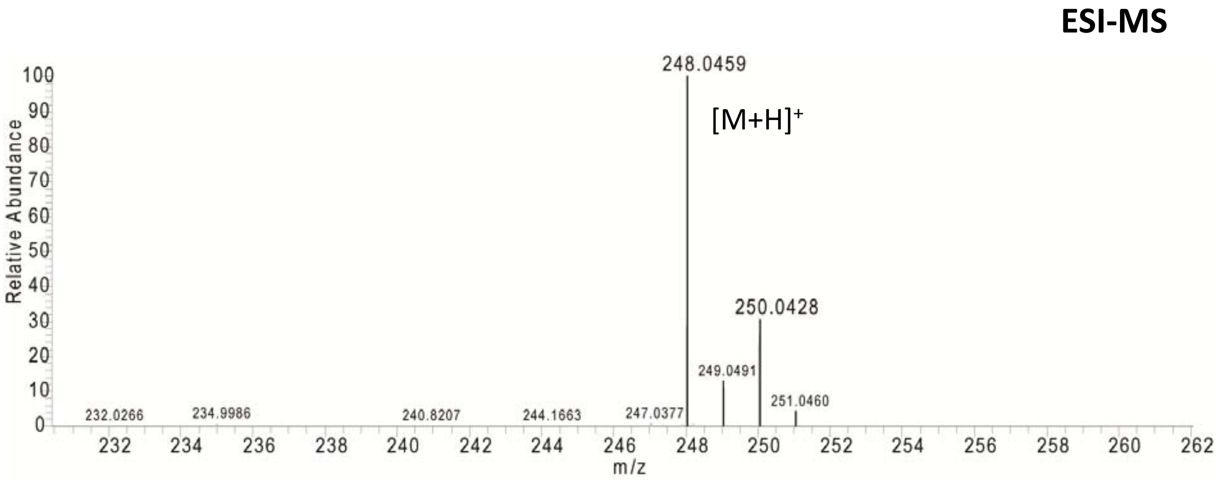
ESI-MS
Relative Abundance
248.0459
[M+H]+
250.0428
249.0491
251.0460
232.0266
234.9986
240.8207
244.1663
247.0377
m/z

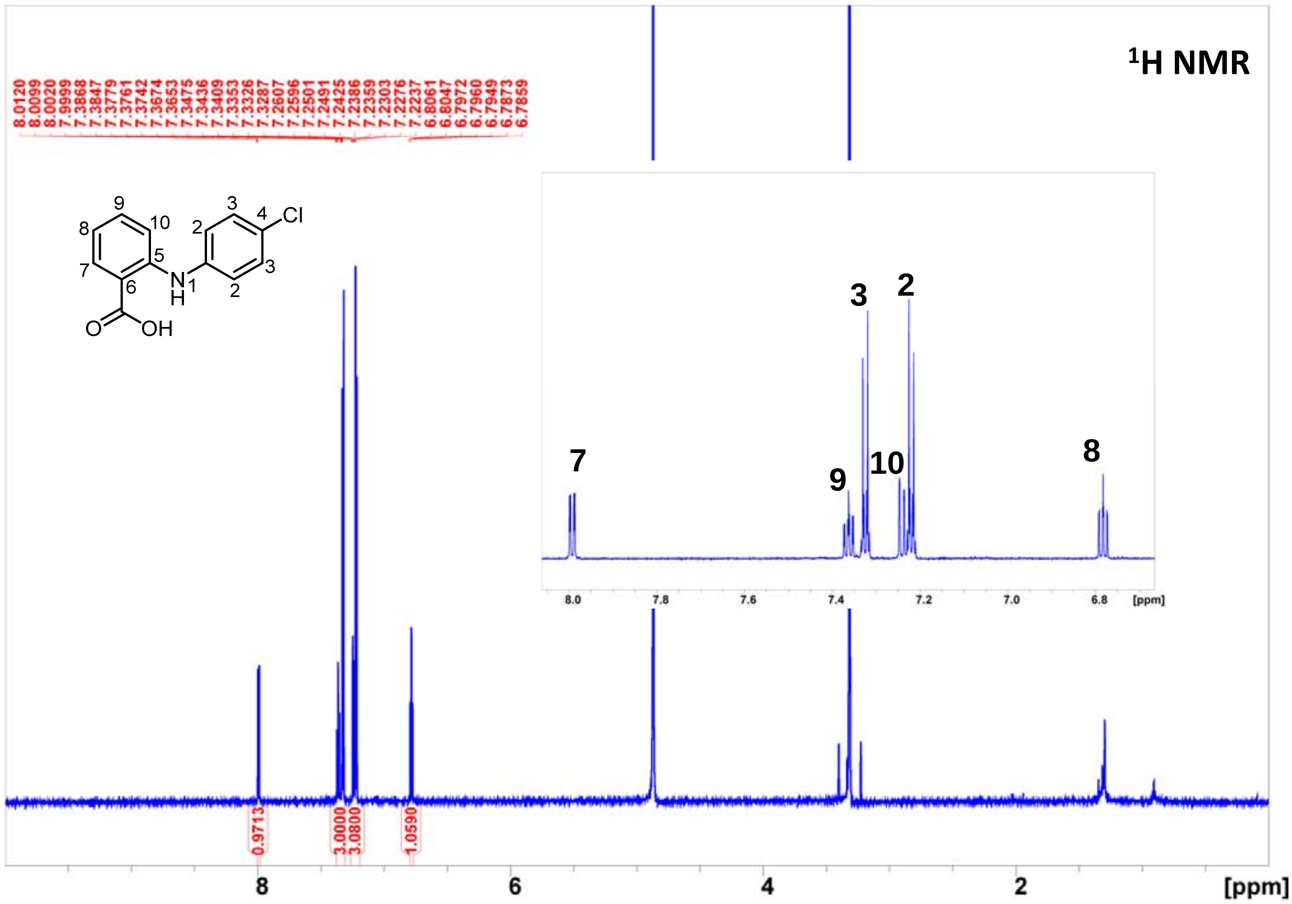
1H NMR
8.0120
8.0099
8.0020
7.9999
7.3868
7.3847
7.3779
7.3761
7.3742
7.3674
7.3653
7.3475
7.3436
7.3409
7.3353
7.3326
7.3287
7.2607
7.2596
7.2501
7.2491
7.2425
7.2386
7.2359
7.2303
7.2276
7.2237
6.8061
6.8047
6.7972
6.7960
6.7949
6.7873
6.7859
0.9713
3.0000
3.0800
1.0590
[ppm]

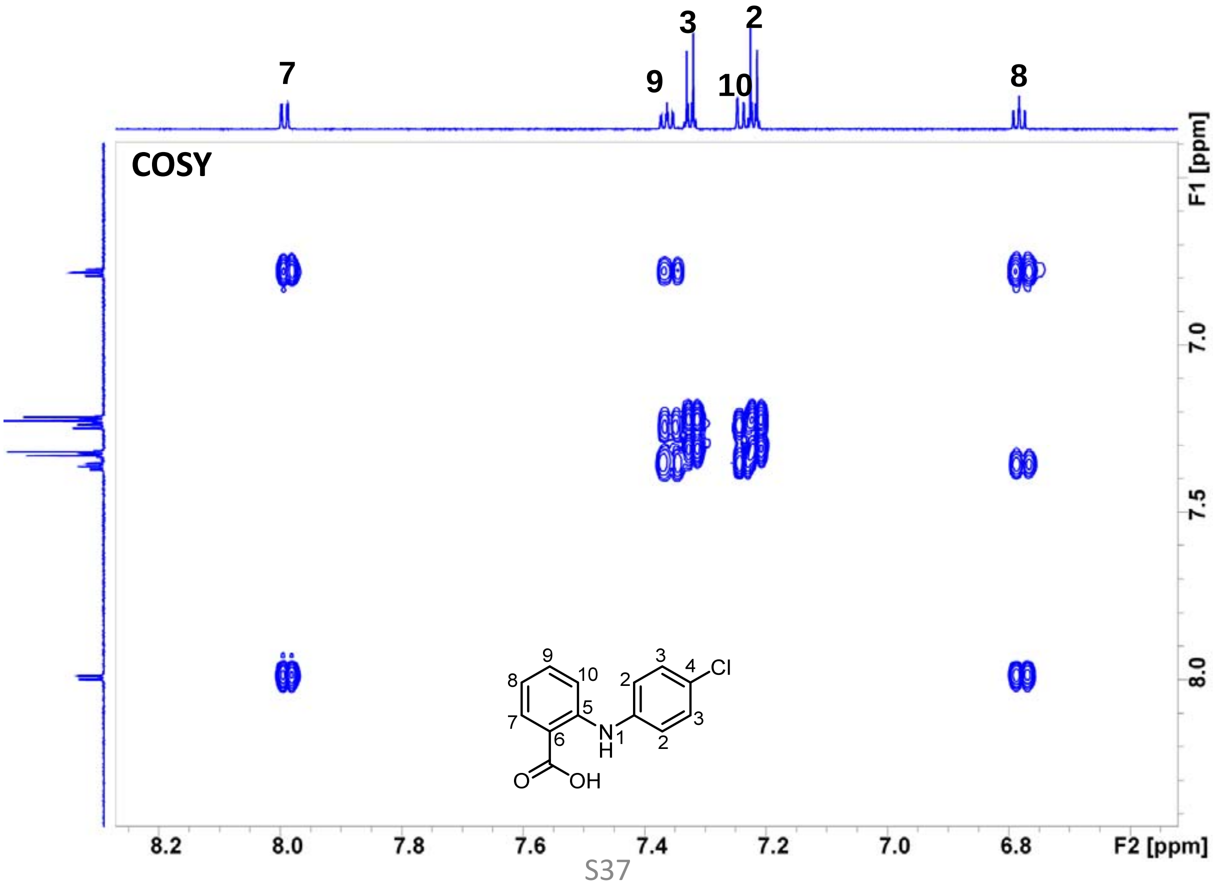
COSY
F1 [ppm]
F2 [ppm]

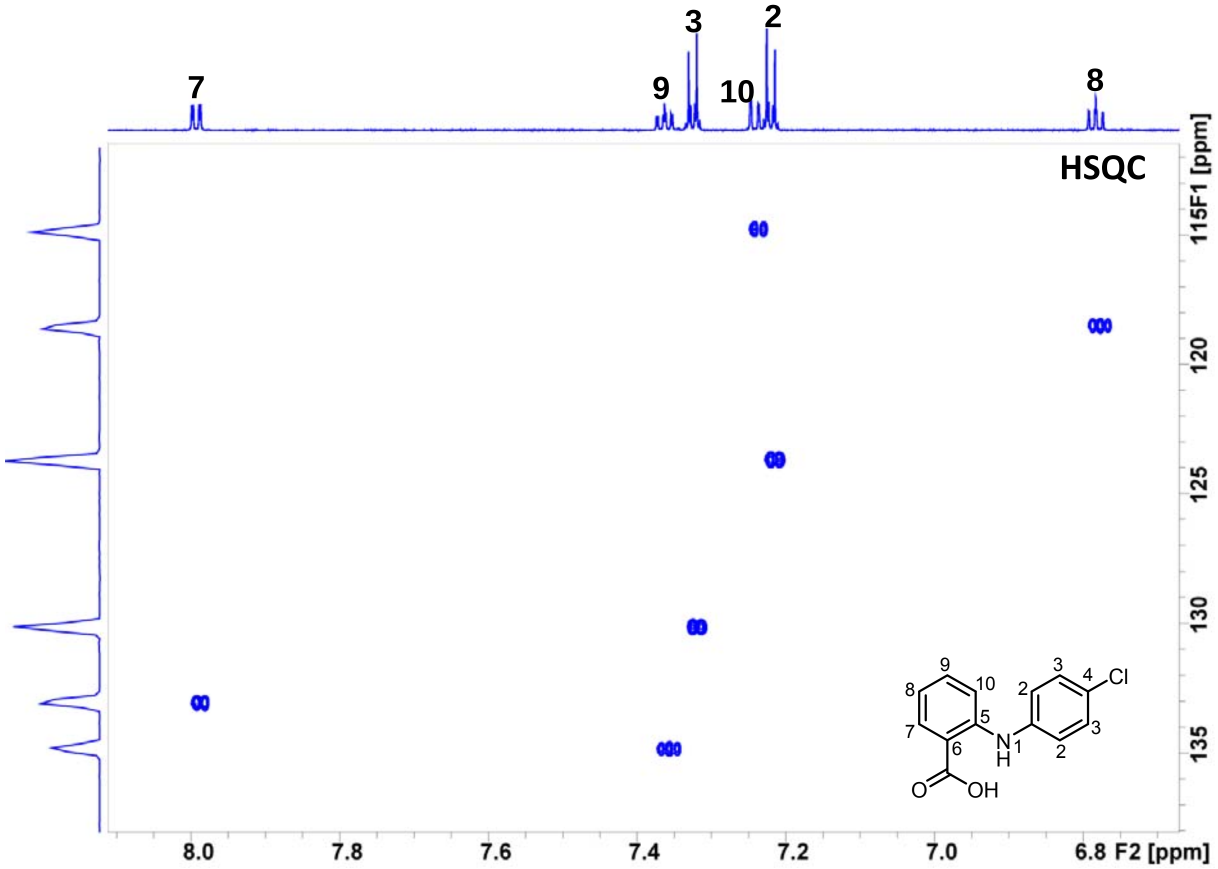
HSQC
7
9
3
10
2
8
F1 [ppm]
115
120
125
130
135
F2 [ppm]
8.0
7.8
7.6
7.4
7.2
7.0
6.8
Cl
OH
O
H
N

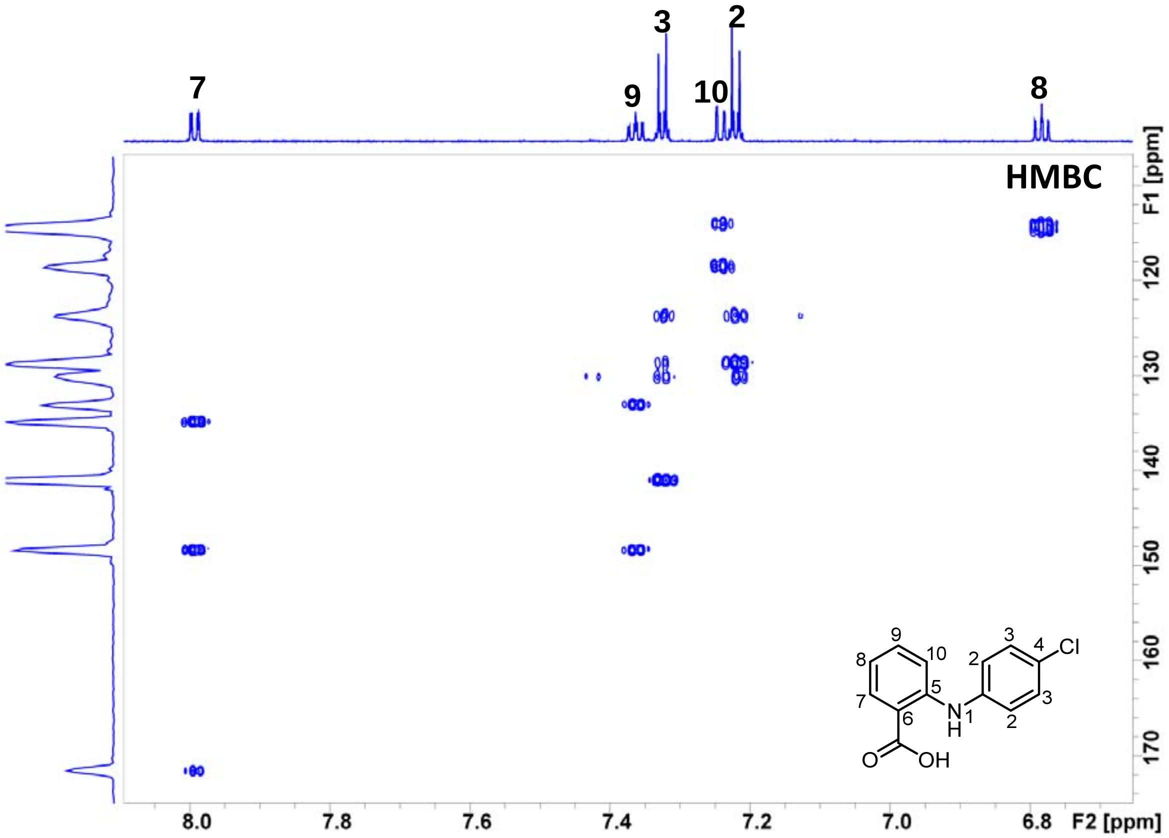
HMBC
7
9
3
10
2
8
F1 [ppm]
120
130
140
150
160
170
F2 [ppm]
8.0
7.8
7.6
7.4
7.2
7.0
6.8
Cl
OH
O
H
N

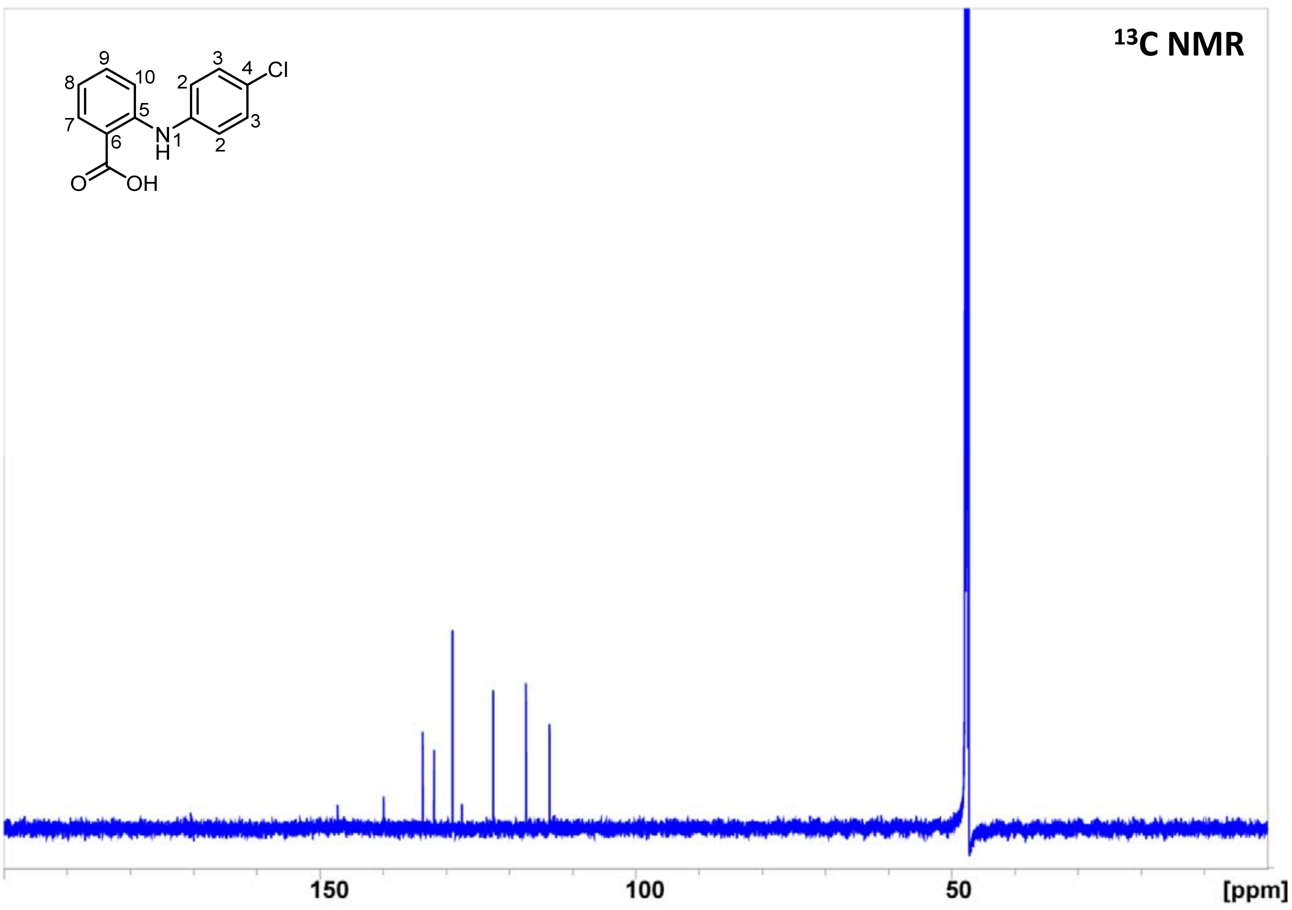
13C NMR
9
10
8
5
7
6
N
1
H
O
OH
3
4
Cl
2
3
2
150
100
50
[ppm]
